# Contagious acquisition of antimicrobial resistance is critical for explaining emergence in western Canadian feedlots—insights from an agent-based modelling tool

**DOI:** 10.3389/fvets.2024.1466986

**Published:** 2025-01-10

**Authors:** Dana Ramsay, Wade McDonald, Michelle Thompson, Nathan Erickson, Sheryl Gow, Nathaniel D. Osgood, Cheryl Waldner

**Affiliations:** ^1^Department of Large Animal Clinical Sciences, Western College of Veterinary Medicine, University of Saskatchewan, Saskatoon, SK, Canada; ^2^Department of Computer Science, University of Saskatchewan, Saskatoon, SK, Canada; ^3^Canadian Integrated Program for Antimicrobial Resistance Surveillance, Public Health Agency of Canada, Saskatoon, SK, Canada

**Keywords:** agent-based model (ABM), simulation model, antimicrobial resistance (AMR), bovine respiratory disease (BRD), antimicrobial use (AMU)

## Abstract

**Introduction:**

Antimicrobial resistance (AMR) is a growing threat to the efficacy of antimicrobials in humans and animals, including those used to control bovine respiratory disease (BRD) in high-risk calves entering western Canadian feedlots. Successful mitigation strategies require an improved understanding of the epidemiology of AMR. Specifically, the relative contributions of antimicrobial use (AMU) and contagious transmission to AMR emergence in animal populations are unknown.

**Materials and methods:**

A stochastic, continuous-time agent-based model (ABM) was developed to explore the dynamics of population-level AMR in *Mannheimia haemolytica* in pens of high-risk cattle on a typical western Canadian feedlot. The model was directly informed and parameterized with proprietary data from partner veterinary practices and AMU/AMR surveillance data where possible. Hypotheses about how AMR emerges in the feedlot environment were represented by model configurations in which detectable AMR was impacted by (1) *only* selection arising from AMU; (2) *only* transmission between animals in the same pen; and (3) both AMU-linked selection and transmission. Automated calibration experiments were used to estimate unknown parameters of interest for select antimicrobial classes. Calibrated parameter values were used in a series of Monte Carlo experiments to generate simulated outputs at both the *pen* and *feedlot* levels. Key model outputs included the prevalence of AMR by class at multiple time points across the feeding period. This study compared the relative performances of these model configurations with respect to reproducing empirical AMR data.

**Results:**

Across all antimicrobial classes of interest, model configurations which included the potential for contagious acquisition of AMR offered stronger fits to the empirical data. Notably, sensitivity analyses demonstrated that model outputs were more robust to changes in the assumptions underscoring AMU than to those affecting the likelihood of transmission.

**Discussion:**

This study establishes a feedlot simulation tool that can be used to explore questions related to antimicrobial stewardship in the context of BRD management. The ABM stands out for its unique hierarchical depiction of AMR in a commercial feedlot and its grounding in robust epidemiological data. Future experiments will allow for both AMU-linked selection and transmission of AMR and can accommodate parameter modifications as required.

## Introduction

The Food and Agriculture Organization of the United Nations (FAO) describes the development of antimicrobial resistance (AMR) as a significant threat of global concern (1), a problem exacerbated by the misuse of antimicrobials in both human healthcare and agricultural settings. Antimicrobials are used in food–animals to control and treat common bacterial diseases; the intensification of livestock production may facilitate the rapid dissemination of infectious agents and is associated with increased antimicrobial use (AMU) (2, 3). Antimicrobial use favours the selection of resistant bacterial pathogens, and international guidelines on prudent AMU in food–animals recommend therapeutic alternatives to antimicrobials (e.g., vaccines) and improvements to animal husbandry that limit the spread of disease (4, 5). The FAO maintains that successful mitigation strategies will crucially depend on ‘an improved understanding of the epidemiology of AMR emergence and spread in animal production’ (3).

A study of over 2.6 million cattle in western Canadian feedlots reported that 97 and 73% of all animals were administered in-feed and individually dosed antimicrobials, respectively (6). The Canadian Feedlot AMU/AMR Surveillance Program (CFAASP) subdivides AMU for bovine respiratory disease (BRD), liver abscesses, and lameness (7, 8). These and other production-limiting diseases are associated with economic costs related to drug and labour expenses, decreases in cattle performance and feed conversion, reduced carcass values, and increased mortalities. BRD is often described as the most common and costly disease affecting North American beef cattle and accounts for 65–80% of mortality in some feedlots (9). The majority (>90%) of individually dosed AMU in the study by Brault et al. (6) was administered to prevent or treat BRD, a finding mirrored in more recent data (2019–2022) published by CFAASP (8). Taken together, these figures underscore the importance of BRD as a determinant of AMU in finishing feedlots.

BRD is a multifactorial disease characterized by the complex interactions between bacterial and viral pathogens, the host’s immune response, and the management and environmental conditions at different phases of the production chain (10). In a recent review, Smith (11) details the ‘accumulation of stress events’ that increase the risk of BRD in post-weaned calves, including the potentially immunosuppressive impacts of repeated handling, long-distance transport, commingling, and processing at feedlot entry (10–12). The FAO notes that ‘times of stress’ (1) are linked to increasing AMU in animal production; similarly, the World Organization for Animal Health (WOAH) emphasizes the role of husbandry practices that reduce stress in farmed animals as central to preserving the efficacy of antimicrobials (13). Owing to the many known risk factors predisposing cattle to acute respiratory disease (11), alternative measures to control BRD are considered complex and costly (14). Consequently, the feedlot industry relies on the continued accessibility and effectiveness of antimicrobials for metaphylaxis, defined here as the mass medication of an entire group of at-risk cattle to control expected outbreaks of BRD (15).

The bacterial pathogens associated with BRD are typically described as commensal in clinically healthy calves (16) and include *Mannheimia haemolytica, Pasteurella multocida, Histophilus somni,* and *Mycoplasma bovis.* The risk of stress-induced susceptibility to opportunistic infection of the respiratory tract with these bacteria varies by type of feeder cattle (10, 11). An assessment of BRD risk informs the selection of on-arrival metaphylaxis (6). It includes factors related to animal age, origin, weather and transport conditions, clinical appearance, and previous vaccination and management history (if known). Younger, lighter and recently weaned calves, and those that are mixed/commingled during procurement (i.e., via the auction market) are generally considered higher risk than their older, heavier, and ranch-direct counterparts (17). In a census of 36 feedlots (6), high-risk cattle were 1.6 times more likely to receive metaphylaxis for BRD than low-risk cattle, and over 100 times more likely to receive a macrolide. The development of resistance to macrolides and other antimicrobial classes of importance to human medicine (18) is of particular concern, given that commensal and environmental bacteria can carry AMR determinants across species. The CFAASP has been monitoring trends in AMR in both bovine respiratory pathogens and enteric bacteria in Canadian feedlot cattle since 2019 (19).

Over the last several decades, little has changed in how the feedlot industry manages BRD in high-risk calves (20), and antimicrobials continue to be efficient tools for disease control (21). In reviewing priority actions to prevent suboptimal AMU in food–animal production, Lhermie et al. (21) advocates that research is needed to assess the impact of alternative strategies using fewer antimicrobials. Gröhn wrote about the complexity of food–animal systems and characterized the relationships between their component parts as interdependent and multidirectional (22). He advocates for using systems science approaches that ‘integrate modelling and mathematics with biological studies’ to build effective policy responses (22). Indeed, mathematical or dynamic models are well suited to represent and study AMR and similarly complex systems characterized by non-linearities, feedback loops, and time-varying variables (23). A review of compartmental and individual or agent-based (ABM) models which investigate the problem of AMR in relation to AMU in human and animal populations was published in 2018 (24); the review noted that models which examined AMR in food–animal settings were critically underrepresented in their dataset.

This work aimed to develop and utilize an ABM to explore the dynamics of population-level AMR emergence in high-risk cattle in a western Canadian feedlot. In particular, this study endeavours to determine the importance of contagious transmission for AMR emergence at the population level relative to AMU-linked selection pressure (3). Careful consideration was given to the clarity and transparency with which the model’s structure, assumptions, and inputs were reported, consistent with best practices (24–26). Furthermore, the model was grounded in robust epidemiological data wherever possible. This study demonstrates the model’s value as a tool for experimenting with strategies that support prudent AMU and limit AMR in the feedlot setting.

## Materials and methods

### Model description

A complete model description is available as a Supplementary file and follows the Overview, Design concepts, and Details (ODD) protocol for detailing agent-based models (ABMs) (27, 28). Key features of the model are briefly described in this section. While the initial model calibrations and subsequent experiments reported here were performed with exclusively higher risk steers entering a small to mid-sized feedlot (6,000–10,000 head, respectively), the following description will note where model parameters can be modified with a user-friendly spreadsheet to customize the output for specific feedlot and cattle characteristics.

The research protocols and procedures for animal data collected for this study at the University of Saskatchewan were approved by the University of Saskatchewan Animal Care Committee (AUP 20190069).

### Purpose

A stochastic, continuous-time ABM was constructed with AnyLogic^®^ 8 simulation software (version 8.8.6) using Java-based code to develop an evidence-based tool that can be used to explore questions related to antimicrobial stewardship in the management of BRD. Model variations in this baseline effort represent hypotheses about how AMR emerges and spreads in the feedlot environment; structural modifications distinguish between (1) a model in which only selection arising from AMU impacts detectable AMR, (2) a model in which only the transmission of resistant bacteria between animals impacts detectable AMR, and (3) a model in which both AMU-linked selection and transmission impacts detectable AMR. A comparison of the relative performance of these models concerning reproducing empirical trends in AMR is a key component of this study.

### Key assumptions underscoring model conceptualization

Representations of this type require that researchers share and scrutinize their assumptions about how the system of interest works (24). The simplifying assumptions defining this model’s scope are first highlighted here and explored in greater detail in the ‘Agents and State Charts’ section. The model is initially described with specific parameter assumptions to facilitate the structural comparisons regarding how AMR emerges in the feedlot. In recognizing the potential for variation in cattle population and management, the model was constructed to readily accommodate these complexities by varying the parameter settings at start-up. These features are explored in sensitivity analyses in the present study, and the ability to modify assumptions via parameter settings is equally available for future experimentation.

#### Feedlot size and population

The particular configuration for this model represented a typical, mid-sized western Canadian feedlot populated by auction-sourced beef steer calves arriving in the fall. The 500–600-pound calves in this model were assumed to represent a moderately high risk of AMR selection and dissemination in the feedlot setting, given the potential for infectious disease spread and exposure to antimicrobials (29). In feedlot research, arrival weight is used as a proxy for BRD risk when other risk factor data are unavailable (29, 30). With the availability of appropriate data, future experiments with the model could incorporate very lightweight calves (i.e., 300–450 pounds) at the highest risk for treatment and morbidity outcomes.

The model simulated the arrival by truck of lightweight steers at random intervals starting 1 October. The cattle deliveries increased in frequency from the start of the ‘fall run’ in October and peaked in November before slowing again until the feedlot reached full capacity in December. To optimize computational efficiency for model construction and calibration, the feedlot consisted of 28 ‘home’ pens of fixed dimensions, one pen for chronically sick animals, and one hospital pen arranged in rows (*n* = 30 total pens); the home pens were filled successively from left to right with 200 calves each. The necessary infrastructure and data are present to incorporate heavier weight and/or heifer calves in different numbers of pens of various sizes in future experiments with the model (31, 32). The default model setting fills individual home pens with calves of the same sex and weight (i.e., BRD risk) category. Pen-level variation in other risk factors, including origin (auction or ranch-direct) or breed (beef or dairy), could similarly be integrated as data becomes available.

#### Calf health and exposure to antimicrobials

Calves were assumed to be healthy at feedlot arrival and were assigned at entry a rate of average daily gain (ADG) drawn from a normal distribution (32, 33). The model simulated the development and management of select syndromes most frequently associated with injectable AMU in the western Canadian feedlot setting, including BRD, bacterial arthritis, and infectious pododermatitis (i.e., foot rot) (6, 8). *M. haemolytica* is one of several bacterial pathogens implicated in the clinical presentation of BRD, particularly in high-risk calves following feedlot arrival (34, 35). For this model, it was assumed that (1) every calf has a population of *M. haemolytica* existing as nasopharyngeal commensals (acknowledging that this organism is not consistently culturable from all calves) (36); and (2) *M. haemolytica* was the causative agent involved in the progression to clinical BRD (37).

Given that the data available to calibrate the model were derived mainly from studies of moderate- to high-risk calves administered macrolides at feedlot entry [e.g., (38, 39)], all animals in the baseline scenarios received metaphylactic tulathromycin by default. However, the choice of metaphylactic antimicrobial (if any) can be probabilistically selected at model initialization (see *Pen* agent).

#### Phenotypic resistance of a representative respiratory pathogen

For each calf in every pen, the resistance status of *M. haemolytica* to select antimicrobial classes (i.e., the presence or absence of detectable AMR in the population of *M. haemolytica*) was monitored throughout the feeding period. The resistance status of *M. haemolytica* was broadly assumed to be representative of the most clinically relevant AMR in the nasopharyngeal microbiome, except *M. bovis*. The selection of *M. haemolytica* as the sentinel pathogen in this study reflects the availability and reliability of temporal resistance prevalence data for this organism. Population-level AMR could evolve due to selection pressure associated with the preventative and therapeutic use of antimicrobials in the model and/or transmission of resistant bacteria among calves.

The probability that the population of *M. haemolytica* in each calf had detectable resistance to each antimicrobial class at feedlot arrival was derived from empirical data in the published literature (38–44). Antimicrobial drugs from the same class (e.g., tetracyclines) or subclass (e.g., 15-membered ring macrolides) were assumed to be equally vulnerable to the relevant resistance mechanism. The model thus simulated the co-selection, co-waning, and co-transmission of detectable resistance for drugs in the same class, with few exceptions. In-feed tylosin use was assumed *not* to co-select for resistance to injectable 16-membered ring macrolides in its subclass, consistent with the finding in Zaheer et al. (45) that subtherapeutic tylosin did not affect the prevalence of resistant *M. haemolytica.*

#### Simulation time and the timing of events

The convenience time unit of the continuous calendar-time model was days. The model was run for 1 year from the entry of the first animals into the feedlot to allow for all pens of cattle to reach finishing weight and to subsequently empty within each cycle. Events in the model occurred at either (1) a fixed time, following the occurrence of another event (e.g., in-feed AMU exposure after a particular number of days on feed [DOF]) or (2) an arbitrary point in time, driven by a daily incidence rate or as a consequence of another event (e.g., receipt of a ‘transmission’ message from a connected calf). In the baseline model, agents were assumed to immediately and perfectly perceive transitions in their health and AMR status that triggered treatment decisions.

### Agents and state charts

The agents in the model were structured hierarchically, with pens filled with calves. Each calf harboured a resistance agent representing a population of *M. haemolytica* with or without detectable resistance to a set of commonly used antimicrobials. All agents and relevant submodels, variable parameters, and data visualizations were contained in the ‘main’ agent, the program entry point and top-level agent for most AnyLogic^®^ models.

#### Feedlot agent

The feedlot consisted of individual pens, each with a cohort of individual calves; the processes of calf arrival and allocating calves to pens were governed at this level. The option to re-sort calves among pens later in the feeding period was disabled for this analysis but is governed by an adjustable parameter. AMU protocols for disease control and treatment were assigned at the feedlot level. They can be specified by the user or randomized across a range of common alternatives developed in consultation with feedlot practitioners. With either option, the selected protocol is applied to the entire feedlot (i.e., all home pens and calves) for one realization of the model. Still, it can be set to vary across multiple iterations within experiments.

#### Pen agent and calf management state chart

The pen agents recorded the filling and emptying of pens by calf agents, and governed the timing and delivery of antimicrobials for disease *control* (Supplementary Figure S1). Management protocols involving antimicrobials—and thus the potential for AMR selection—included each of (1) on-arrival injectable metaphylaxis for BRD management; (2) in-feed prophylaxis for histophilosis and liver abscess prevention; and (3) in-feed prophylaxis for outbreak control when 10% or more calves in a shared pen were diagnosed with foot rot in a single feeding period. The options and likelihoods of available metaphylactic and prophylactic protocols in the model are outlined in Supplementary Figures S3, S4, respectively.

Calves were assumed to be ready for slaughter when the average weight of the animals in a single pen reached the target market weight selected randomly from a uniform distribution (31, 32, 46) (Table 1). Pens were subsequently depopulated to simulate the shipment of finished calves to a processing plant, and pen summary data were exported.

**Table 1:** Values and sources for parameters in the baseline and calibrations versions of the agent-based feedlot model.

Parameter	Condition	Value in baseline model	Value selected for calibration experiments	Source or rationale, if applicable
Feedlot and pen parameters				
Number of cattle per pen		200, user defined	200	Representation of a moderate-sized feedlot; optimization of computational effort
Number of pens across		10, user defined	10	
Number of pens high		5, user defined	3	
Cattle parameters				
Proportion of high-risk animals entering feedlot		0–100%, user defined	100%	
Proportion of steers entering feedlot		0–100%, user defined	100%	
Arrival weight for high-risk animals	Applies to recently weaned and/or lighter-weight calves	Value selected from uniform distribution, range 500–600 pounds	Value selected from uniform distribution, range 500–600 pounds	(31, 32)
Arrival weight for low-risk animals	Applies to backgrounded and/or heavier-weight yearlings	Value selected from uniform distribution, range 800–900 pounds	N/A No low-risk, heavyweight animals in calibration	(31, 32)
Average daily gain (ADG) for healthy steers	Applies to steers with no BRD or arthritis history	Value selected from normal distribution with *μ* = 3.46 and *σ* = 0.46 pounds per day	Value selected from normal distribution with *μ* = 3.46 and *σ* = 0.46 pounds per day	(32, 33)
Average daily gain (ADG) for healthy heifers	Applies to heifers with no BRD or arthritis history	Value selected from normal distribution with *μ* = 3.00 and σ = 0.37 pounds per day	N/A All animals in calibration are steers	(32, 33)
Average daily gain (ADG) for animals of either sex being treated in the hospital pen	Applies to animals temporarily housed in the hospital pen for arthritis treatment	Fixed value of 0.00 pounds per day	Fixed value of 0.00 pounds per day	Model parsimony; consultation with feedlot veterinarians
Percentage change in ADG for animals of either sex with the first case of BRD	Applies for remainder of feeding period to animals with single diagnosis (i.e., first case) of BRD	−0.64%	−0.64%	(48)
Percentage change in ADG for animals of either sex with first or subsequent relapse of BRD	Applies for remainder of feeding period to animals with more than one diagnosis (i.e., one or more relapses) of BRD	−5.77%	−5.77%	(48)
Percentage change in ADG for arthritis-affected animals of either sex before 60 DOF	Applies for remainder of feeding period to animals with first or subsequent arthritis diagnoses before 60 DOF and following treatment in hospital pen	−0.69%	−0.69%	(49)
Percentage change in ADG for arthritis-affected animals of either sex after 60 DOF	Applies for remainder of feeding period to animals with first or subsequent arthritis diagnoses after 60 DOF and following treatment in hospital pen	−1.38%	−1.38%	(49)
Target market weight for slaughter at federal facility, healthy animals	Applies to healthy animals in regular pens; pen is shipped as unit when average weight of animals reaches selected target	Value selected from uniform distribution, range 1,325–1,500 pounds	Value selected from uniform distribution, range 1,325–1,500 pounds	(31, 32, 46)
High target finishing weight for slaughter, chronic pen animals		Fixed value of 1,200 pounds	Fixed value of 1,200 pounds	Consultation with feedlot veterinarians
Low target finishing weight for slaughter, chronic pen animals		Fixed value of 900 pounds	Fixed value of 900 pounds	Consultation with feedlot veterinarians
Disease parameters				
Probability of first BRD relapse in high-risk animals	Conditional on having had a first case of BRD	21.64%	21.64% All animals in calibration are high-risk	Empirical data from approximately 55,000 high-risk calves with first cases of BRD after tulathromycin metaphylaxis (2012–2016)
Probability of second or third BRD relapse in high-risk animals	Conditional on having had a first or second relapse of BRD, respectively	35.80%	35.80% All animals in calibration are high-risk	Empirical data from approximately 12,000 high-risk calves with first relapses of BRD after tulathromycin metaphylaxis (2012–2016)
Probability of first BRD relapse in low-risk animals	Conditional on having had a first case of BRD	26.19%	N/A No low-risk animals in calibration	Empirical data from approximately 5,600 low-risk calves with first cases of BRD after oxytetracycline metaphylaxis (2012–2016)
Probability of second or third BRD relapse in low-risk animals	Conditional on having had a first or second relapse of BRD, respectively	40.45%	N/A No low-risk animals in calibration	Empirical data from approximately 1,450 low-risk calves with first relapses of BRD after oxytetracycline metaphylaxis (2012–2016)
Probability of first arthritis relapse	Conditional on having had a first case of arthritis	11.58%	11.58%	Empirical data from approximately 6,000 fall-placed calves with first cases of arthritis (2007–2020)
Probability of second arthritis relapse	Conditional on having had a first relapse of arthritis	20.46%	20.46%	Empirical data from approximately 700 fall-placed calves with first relapses of arthritis (2007–2020)
Probability of third arthritis relapse	Conditional on having had a second relapse of arthritis	19.58%	19.58%	Empirical data from approximately 145 fall-placed calves with second relapses of arthritis (2007–2020)
Cumulative percentage of footrot-affected animals triggering pen-level ‘outbreak protocol’	Applies when threshold percentage of animals in shared pen are diagnosed with footrot in single feeding period	10%	10%	Consultation with feedlot veterinarians
Chronic pen parameters				
Percentage of animals assigned to ‘high target finishing weight’ group at chronic pen entry		33.3%	33.3%	Model parsimony
Percentage of animals assigned to ‘low target finishing weight’ group at chronic pen entry		33.3%	33.3%	Model parsimony
Percentage of animals assigned to ‘euthanasia’ group at chronic pen entry		33.3%	33.3%	Model parsimony

#### Cattle agent

Each cattle agent characterized the health status of a single calf, and governed the timing of and response to antimicrobials for disease *treatment* (i.e., injectable AMU for therapeutic indications) (Supplementary Figure S2). Healthy calves became sick with BRD, bacterial arthritis, or foot rot, as described below; the options and likelihoods of available therapeutic protocols in the model are outlined in Supplementary Figures S5–S7, respectively. The calf’s location in the feedlot and mortality status were similarly governed at the individual animal level (Supplementary Figure S8).

#### Health status state chart

**Figure 1:**
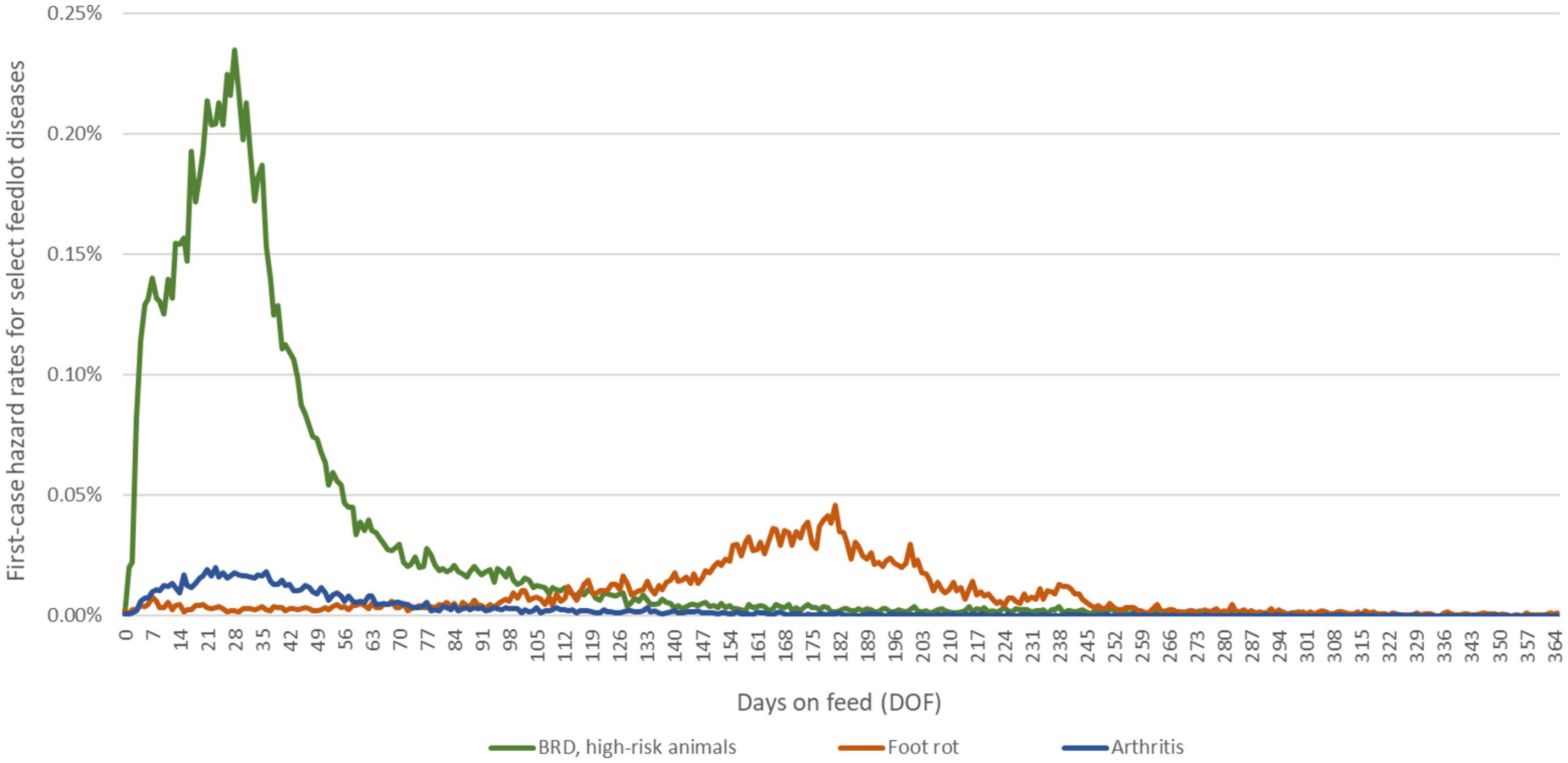
Daily hazard rates for first cases of BRD, foot rot and arthritis over the feeding period. Epidemiological curves are derived from empirical data from large, private veterinary practices in western Canada. The data for BRD represent approximately 590,000 high-risk, fall-placed animals at feedlot entry over 5 years (2012–2016). The first-case hazard rate for BRD used in the calibration experiments was specific to high-risk calves that received metaphylactic tulathromycin at feedlot arrival; in future experiments, the rate can be adjusted for other metaphylaxis protocols by the risk ratios calculated in O’Connor et al. (47). The data for foot rot and arthritis represent approximately 600,000 fall-placed animals over 13 years (2007–2020) provided as anonymized summaries.

Calves became sick at a specified rate per day on feed (i.e., a daily hazard rate dependent on the number of days since feedlot entry). They were immediately administered the prescribed antimicrobial regimen upon transitioning to a disease state. The daily hazard rates for the first case of each disease were drawn from empirical distributions reflecting temporal/seasonal and weight-based changes in regional disease risk shared by large private veterinary practices (Figure 1). The first-case hazard rate for BRD in high-risk calves was specific to animals who received metaphylactic tulathromycin at feedlot entry (overall first treatment rate of 10%); the rate can be adjusted for other metaphylactic protocols, including ‘no metaphylaxis’ by the risk ratios reported in a recent meta-analysis of injectable antimicrobial options for BRD control (47). Historical on-arrival AMR data were assumed to be reflected in the first-case hazard rate for BRD obtained from feedlot operations and used in the calibrations. In subsequent experiments with the model, the first-case hazard rate for BRD for an individual calf could default to the equivalent of that for ‘no metaphylaxis’ *if* the population of *M. haemolytica* for that calf was resistant to the antimicrobial used for metaphylaxis.

BRD and arthritis-affected calves could experience one or more relapses, requiring additional individually dosed treatments after the first case of either disease (Supplementary Figure S2). Calves who received treatment for a first case experienced a first relapse with a fixed probability; second and third relapse probabilities were similarly conditional on the calf having experienced a first and second relapse, respectively. This was possible because the model tracks the individual treatment history of each calf. The probabilities of subsequent relapses for each disease were calculated from the same empirical data used to derive first-case hazard rates (see Table 1). First relapse probabilities were calculated by dividing the cumulative number of first relapses by the cumulative number of first cases over the feeding period (Table 1); subsequent relapse probabilities were calculated the same way. Calves that transitioned to a ‘disease’ state due to BRD or arthritis were assigned a reduced rate of ADG (i.e., gained weight more slowly) for the duration of their time in the feedlot (48, 49); the reductions in ADG were additive for calves affected by both diseases over the feeding period.

To best match empirical data available for model calibration, relapses were characterized as the failure of a previous therapy to adequately treat the underlying infection, prompting the continuation of symptomatic disease (50). It was assumed that relapses due to AMR-linked treatment failure were reflected in the total number of relapses in the historical data used for the calibrations. In subsequent experiments with the model, additional BRD relapses will be possible *if* the current pen-level prevalence of resistance to the administered drug exceeds the baseline probability of treatment failure (see BRD treatment failure loop, Supplementary Figure S2). While not all treatment failures will be due to AMR, the exact probability is unknown; the assumption that AMR above the baseline will result in additional treatment failure, therefore, represents a worst-case scenario. Calves with baseline or resistance-linked treatment failure remain in the ‘disease’ state following the therapeutic interval (see Table 2) and receive the next antimicrobial in the treatment/relapse protocol (Supplementary Figure S5).

**Table 2:** Pharmacokinetic and initialization parameters for probabilistically selected antimicrobials used for prophylaxis, metaphylaxis, or treatment in the feedlot model and for antimicrobials to which acquired resistance over the feeding period is of interest.

Antimicrobial class	Antimicrobial drug (example of active ingredient)	Antimicrobial drug (example of trade name)	Reason for use in feedlot model^a^	Average probability (%) of detectable resistance at arrival^b^	Withdrawal period (days)^c^	Therapeutic interval (days)^d^
Aminoglycosides	Spectinomycin	N/A	N/A	1.7	N/A	N/A
Cephalosporins	Ceftiofur CFA Ceftiofur HCl	Excede 200 Excenel RTU EZ	T T	0.0 0.0	13 3	9.2 1.1
Fluoroquinolones	Enrofloxacin	Baytril 100	T	0.4	36	0.7
Macrolides (15-membered ring)	Gamithromycin Tulathromycin	Zactran Draxxin	M, T M, T	2.4 2.4	49 44	6.9 8.3
Macrolides (16-membered ring)	Tildipirosin Tilmicosin Tylosin^e^	Zuprevo Micotil Tylan 100	M M, T P	4.3 4.3 4.3	42 28 0	26.3 3.6 0.1
Potentiated sulphonamides	Sulphadoxine Trimethoprim	Borgal	T	2.3 0.3	10 10	1.6 0.3
Penicillins	Ampicillin Penicillin	Polyflex Procaine Penicillin G	N/A T	1.7 1.7	6 5	0.8 0.1
Phenicols	Florfenicol Florfenicol/ flunixin	Nuflor Resflor	T T	0.1 0.1	55 60	3.2 3.2
Tetracyclines	Chlortetracycline^f^ Oxytetracycline	Chlor 100 Granular Medicated Premix Liquamycin LA-200	P M, T	4.9 4.9	5 48	2.0 2.6

a Abbreviations indicating reason for use in feedlot model: P, prophylaxis; M, metaphylaxis; T, treatment; N/A, not used in model.

b Probability of detectable phenotypic resistance on feedlot arrival for each antimicrobial was derived from recent studies of healthy feedlot cattle in western Canada (38–44). In the calibration experiments, the probability was a weighted average of the extracted prevalence data; in the Monte Carlo experiments, the probability was randomly drawn from a modified PERT distribution where the 95% CI were the minimum and maximum values and the average value was the most likely value (mode).

c The withdrawal period in days reflects those reported in the Compendium of Veterinary Products—Canada edition (54).

d The therapeutic interval is a crude estimate of the effective duration of selective pressure (i.e., the period over which the drug is active and selection for resistance is possible). The therapeutic interval for each antimicrobial was estimated from its reported serum elimination half-life in cattle (55–64), multiplied by three.

e Prophylactic tylosin use did not co-select for *Mannheimia haemolytica* resistance to the other 16-membered ring macrolides in its subclass, consistent with the observation that ‘the in-feed levels of tylosin (have) no effect on the prevalence of *M. haemolytica*’ (45).

f Chlortetracycline at both the ‘high’ and ‘low’ dosages were fully linked to each other and to oxytetracycline. When the ‘low dose’ of chlortetracycline was used, the calibrated ‘selection probability’ for tetracyclines was adjusted by a multiplier (0.2) that reflects the average concentration of that regimen relative to the ‘high dose’ regimen. The multiplier was estimated from AMU data collected by the Canadian Integrated Program for Antimicrobial Resistance Surveillance [referenced in Hannon et al. (99)] and a series of expert interviews with feedlot veterinarians.

Calves treated for foot rot in the empirical data available for model parameterization were assumed to have uncomplicated cases of infectious pododermatitis that responded to therapy. However, some of these calves would have been retreated if they were misdiagnosed, treated too late, or if the disease appeared in another foot. The model accounted for this possibility as calves re-entered the total population at risk of infection after receiving individually dosed treatment (i.e., calves returned to a ‘healthy’ state by default after the therapeutic interval had elapsed for the administered antimicrobial, see Table 2). Long-term ADG was not substantially affected in calves with foot rot in a previous report (51); thus, the growth rate did not change for these animals.

#### Location and life state charts

All animals in the same home pen were connected via a transmission network or ‘spatial neighborhood’ configured at model initialization. The user can reconfigure networks in future experiments to include distance-based connections and/or connections between animals in adjacent pens. Calves being treated for first or subsequent cases of arthritis were moved to a specialty pen for acutely sick animals requiring multiday therapeutic regimens, designated the ‘hospital pen’ (see Supplementary Figure S6); the calf’s connections were reconfigured to include their temporary pen-mates during their hospital stay. These animals returned to their home pen or chronic pen after their final doses were administered. They were the only potential vectors for the interpen spread of resistance in the baseline model where both cattle re-sorting and connections between pens are disabled. Calves did not gain weight while housed in the hospital pen and were temporarily assigned an ADG equal to zero (i.e., their weights remained constant); once they returned to their home pens, the animals resumed weight gain at the reduced rate as described in Table 1. If the animal’s home pen had been depopulated (i.e., sent for slaughter) before the calf returned, the animal was instead transferred to the ‘chronic pen’, as there was no ‘rail pen’ specified in the model.

Chronically sick animals, including a proportion of heavyweight calves with arthritis or calves experiencing a third relapse from BRD or arthritis, were similarly moved to this second specialty pen, designated the ‘chronic pen’. Calves housed in this pen did not receive prophylactic (i.e., in-feed) or therapeutic antimicrobials to prevent or treat disease, given that they had not and were not expected to respond to established treatment protocols; these animals continued to gain weight at the reduced rate of ADG for their diagnosis (48, 49). As in the hospital pen, the connections of calves in the chronic pen were reconfigured to include their new pen-mates.

At chronic pen entry, calves were probabilistically assigned to one of three ultimate destinations consistent with what might occur in large feedlot operations. One-third of the calves who entered the chronic pen were ultimately euthanized. The remainder were evenly split between (1) those who were eventually shipped to a slaughter plant (as with healthy calves), as they achieved near-to-target weights in a reasonable time frame; and (2) those who were slaughtered at a reduced final weight (see Table 1). The disposition and weight of chronically sick animals were evaluated every 7 days. The animals were sent for slaughter when they reached the minimum finishing weights specified in Table 1. Animals in the chronic pen which failed to reach their minimum target weight before the end of the feeding period were also euthanized.

**Figure 2:**
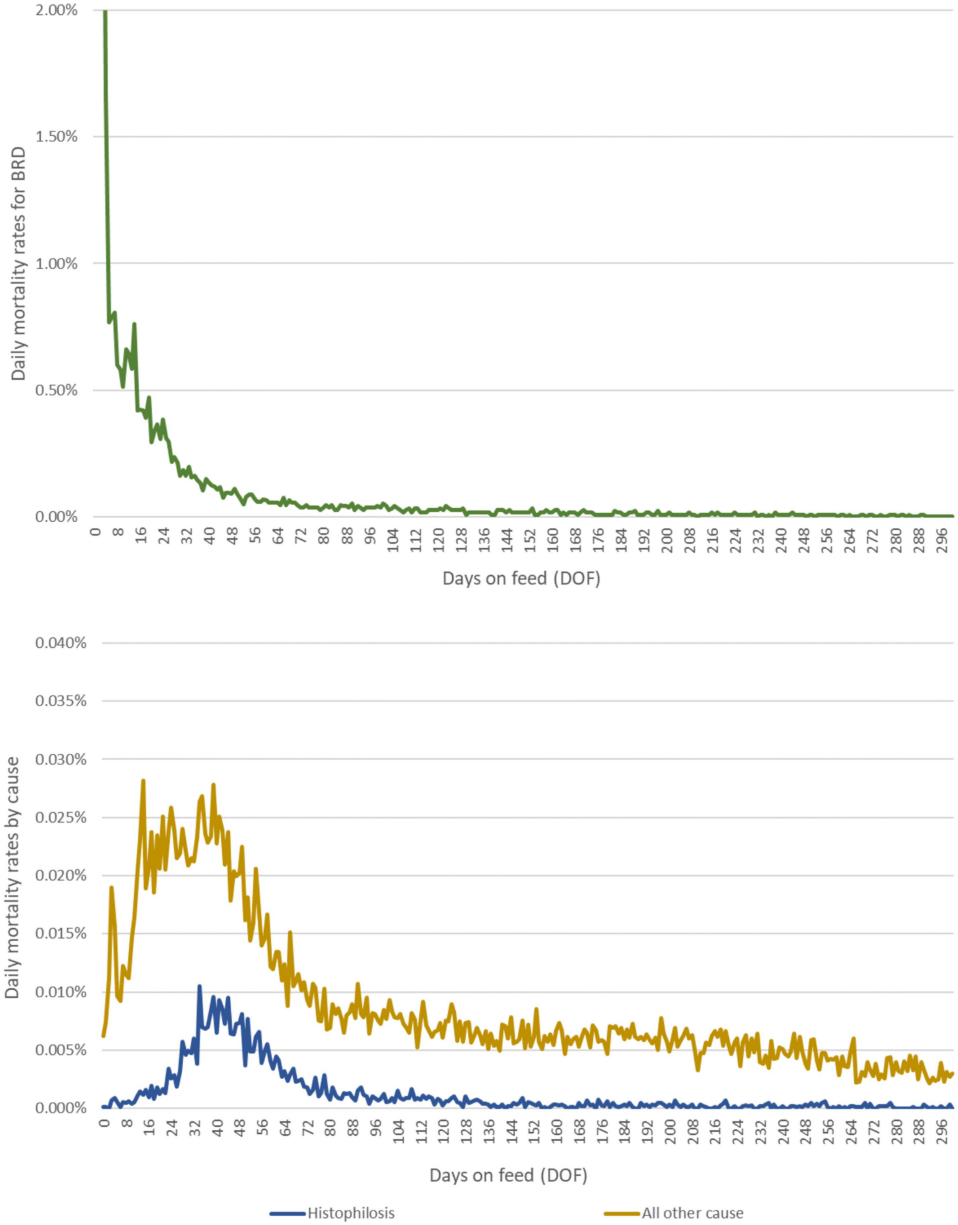
Daily mortality rates due to **(A)** BRD and **(B)** histophilosis and other causes over the feeding period. Epidemiological curves are derived from empirical data from a large, private veterinary practice in western Canada. These data represent approximately 700,000 high-risk, fall-placed animals at feedlot entry over 5 years (2012–2016).

Calves in any pen type could die of disease before reaching their target weight, and this was captured in the model by a daily mortality rate dependent on the number of days since feedlot entry. Daily mortality rates subdivided by cause were drawn from empirical distributions reflecting temporal/seasonal and ecological changes in risk (see Figures 2A,B). It was assumed that death due to BRD was conditional on having experienced at least a first case of BRD requiring treatment with antimicrobials (overall BRD mortality rate of ~3%). While animals die from BRD without being diagnosed and treated, empirical data attributing death loss to treated vs. untreated animals were not available. This assumption does not impact total death losses in the model.

#### Resistance agent

**Figure 3:**
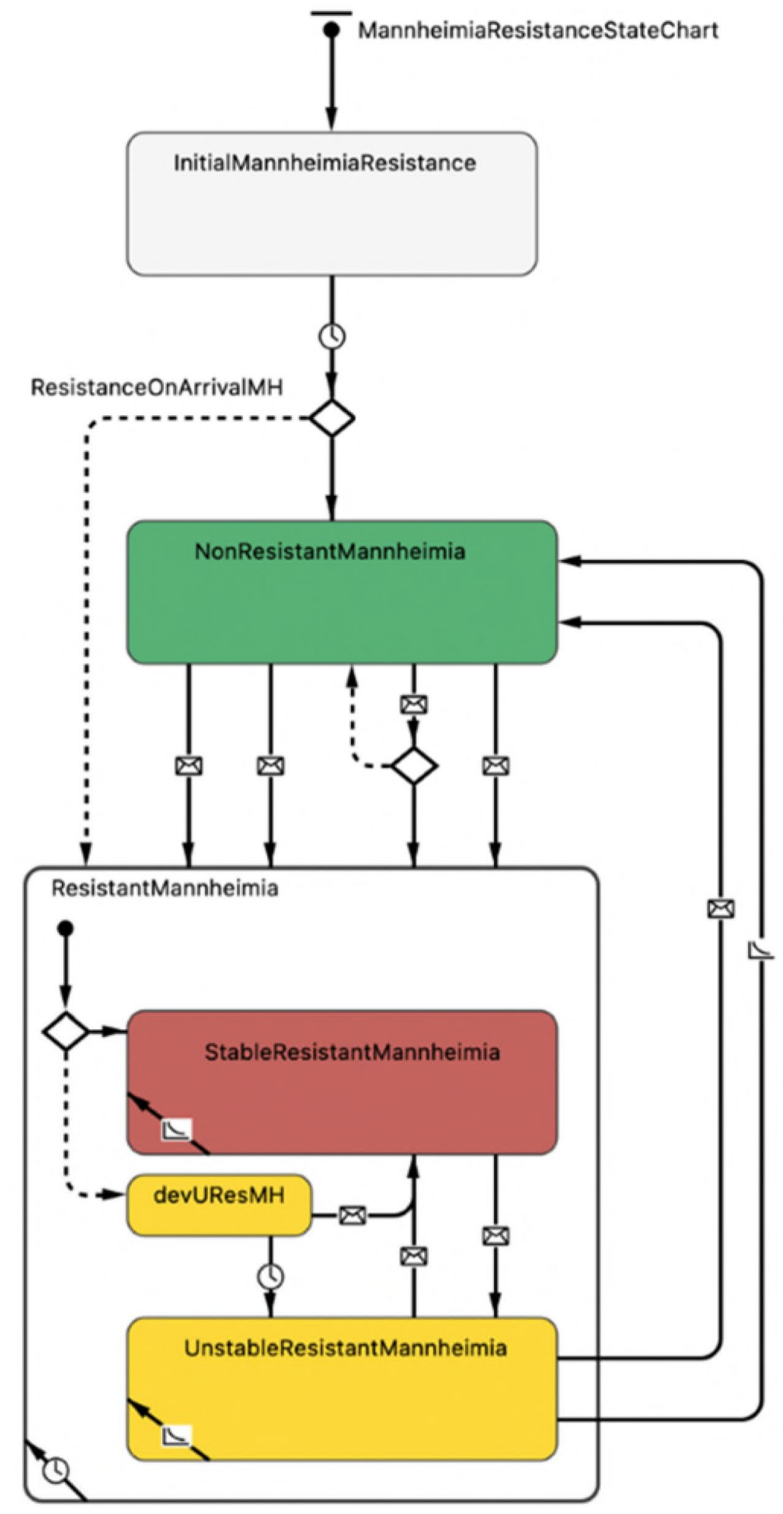
Representation of AMR status for the population of *Mannheimia haemolytica* in the nasopharynx of each calf in the agent-based model. The first branch, demarcated by a ‘diamond’ symbol, indicates the possibility of two outcomes, where (1) resistance is present on arrival (default, dotted line, to a composite ‘resistant’ state with substates) or (2) resistance is not present on arrival (solid line, to a ‘non-resistant’ state) as derived from empirical data. Transitions between the ‘non-resistant’ and ‘resistant’ states demarcated by ‘envelope’ symbols depend on (1) the receipt of ‘selection’ or ‘transmission’ messages or (2) the receipt of ‘co-selection’, ‘co-transmission’ or ‘co-waning’ messages from linked antimicrobial drugs belonging to the same class or subclass and undergoing the same transition. Within the composite ‘resistant’ state, transition arrows demarcated by ‘envelope’ symbols reflect messages related to changing antimicrobial exposure and dictate whether resistance in the *M. haemolytica* population can wane (i.e., is ‘unstably resistant’). *The ‘devUResMH’ (developing unstable resistance) substrate is a temporary delay state within which resistance present on arrival or resistance acquired contagiously cannot wane nor be transmitted to pen-mates. Resistance can become ‘stable’ if the relevant antimicrobial drug is used, or else it becomes unstable with the potential to wane after 48 h (see ‘*M. haemolytica* Resistance State Chart’ section).

The emergent AMR status for the population of *M. haemolytica* in the nasopharynx of each calf was governed at the level of resistance agent. The resistance selection probabilities, resistance waning rates, and baseline contact rates were unknown values expected to differ by drug class. The dynamics of resistance acquisition and loss for each antimicrobial class are thus directed by separate and mutually exclusive state charts (Figure 3).

**Figure 4:**
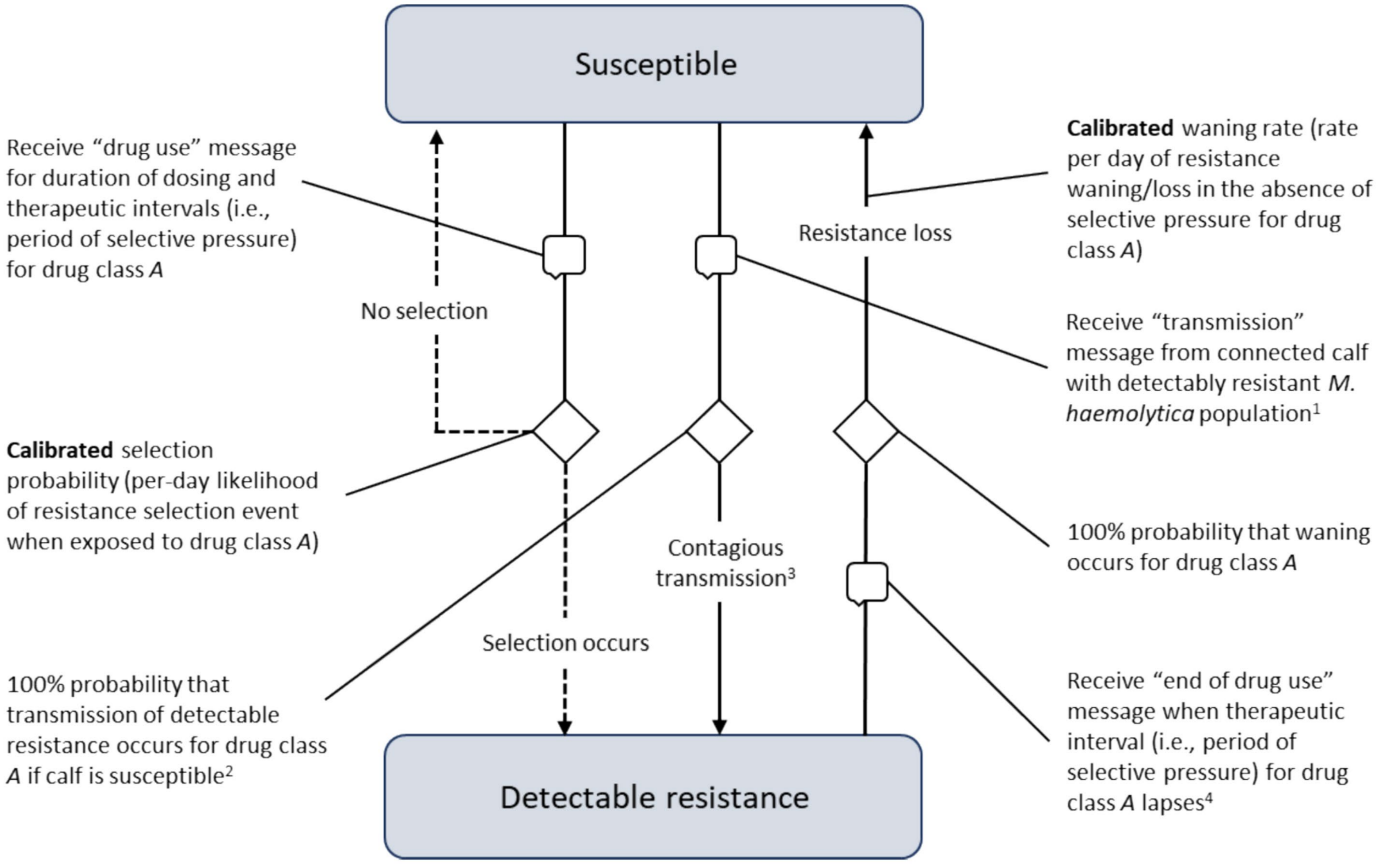
Processes underlying the acquisition and waning of phenotypic resistance for the sentinel nasopharyngeal organisms unique to each calf. Transitions demarcated by ‘speech bubble’ symbols are triggered by receiving a message from another agent in the model (i.e., the calf’s home pen or a connected calf in the same pen). ^1^Transmission messages were sent from animals with detectable resistance to a randomly selected pen-mate at the calibrated contact rate, and the calibrated stress multiplier adjusted the baseline contact rate. There was a 48-h delay before on-arrival resistance could be transmitted to pen-mates to establish the appropriate population-level resistance at the simulation start. ^2^When a calf received a transmission message was received with a susceptible *Mannheimia haemolytica* population, the contact was always ‘effective’ (i.e., resulted in contagious transmission). ^3^There was a 48-h delay before contagiously acquired resistance could wane and/or be transmitted to pen-mates to permit the resistant population to become established in the affected animal. ^4^Resistance acquired selectively or contagiously could not wane if the animal was being actively exposed to (i.e., within the therapeutic interval of) the drug class of relevance; further, there was a 48-h delay before on-arrival resistance could wane to establish the appropriate population-level resistance at simulation start.

#### *M. haemolytica* resistance state chart

Local *M. haemolytica* populations acquired detectable resistance at the calibrated ‘selection probability’, the per-day likelihood of developing a resistant phenotype in response to antimicrobial drug exposure (Figure 4). The selection probability was active during therapy (i.e., the dosing period; see ‘Treatment Agent and State Chart’ section) and for the period of selective pressure following the final—often the only—dose (i.e., the therapeutic interval, see Table 2). Resistant *M. haemolytica* during active exposure to the relevant drug was considered ‘stable’ and could not wane. After the therapeutic interval had elapsed and in the absence of selective pressure for a particular antimicrobial, the population of *M. haemolytica* could then lose its detectable resistance at the calibrated ‘waning rate’ (i.e., was ‘unstably’ resistant). Resistance present on arrival was assumed to be temporarily stable in a 48-h ‘delay’ state, such that it could not transmit or wane *even in the absence of* selective AMU pressure. This lag was included to establish the expected probabilities of population-level resistance during the period the pen was filling.

In addition to selection, local *M. haemolytica* populations acquired detectable resistance at the calibrated ‘contact rate’ through contagious spread from a connected calf in a shared pen (Figure 4). The calibrated parameter was the daily rate at which an animal with detectable resistance sent a ‘transmission’ message to a randomly selected pen-mate. When the message was received by a calf with a susceptible *M. haemolytica* population, it was assumed to be a transmitting contact. As with on-arrival resistance, detectable resistance acquired contagiously was subject to a 48-h lag before it could (1) wane at the calibrated rate, given the absence of selective pressure for the relevant drug; or (2) transmit at the calibrated contact rate to unaffected pen-mates. This lag was included to permit the resistant population to establish and to facilitate calibrated parameter estimation. A similar lag between exposure and transmission would be expected in a true biological infection.

**Figure 5:**
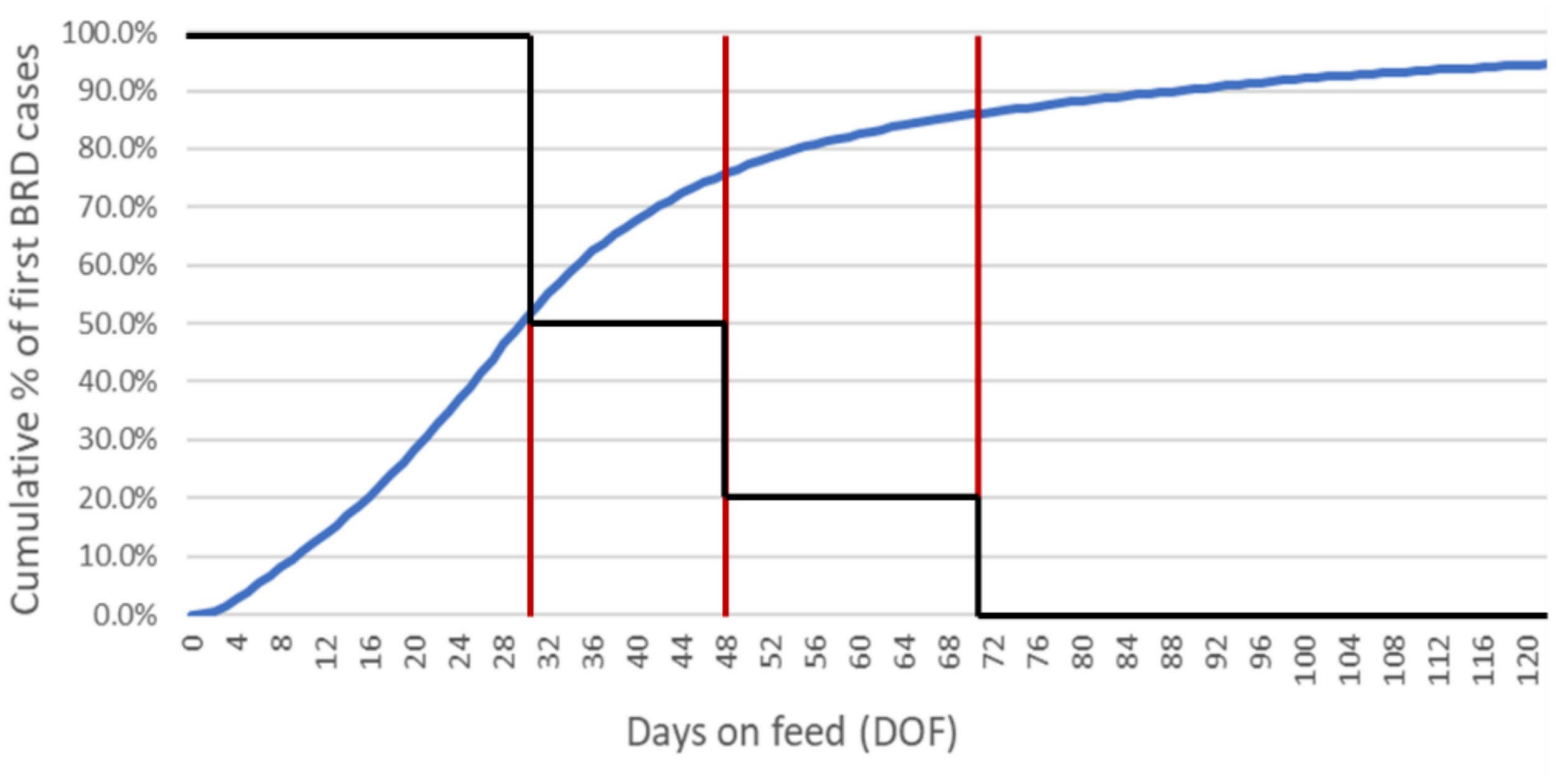
Cumulative incidence of first-case BRD diagnoses in high-risk calves through 120 DOF. The epidemiological curve (blue) is derived from empirical data from a large, private veterinary practice in western Canada. These data represent approximately 590,000 fall-placed animals over 5 years (2012–2016). A step function (black) is superimposed over the cumulative incidence curve; the red vertical lines delimit where unique regions of the curve (i.e., distinct periods of physiological stress/pathogen shedding) correspond to DOF. By 30 DOF, 50% of the high-risk animals who will get sick have a first case of BRD; by 48 DOF, 75% will have a first case of BRD; by 70 DOF, 85% will have a first case of BRD. The stress effect multiplier adjusts the baseline contact rate to account for changing transmission potential over the feeding period as determined by the step function (e.g., after 70 DOF when the step function reduces to zero, the multiplier has no impact on the baseline contact rate). The impact of the calibrated stress multiplier on the contact rate in the ‘baseline’ scenario is reduced by half (from 10 to 5) between 30 and 48 DOF, reduced to 2 between 48 and 70 DOF, and reduced to 0 after 70 DOF. ^*^In the sensitivity analyses where the effect of the calibrated stress multiplier on the contact rate is *minimized*, the step function is reduced from 10 to 0 after 21 DOF. ^**^In the sensitivity analyses where the effect of the calibrated stress multiplier on the contact rate is *maximized*, the step function remains at 10 until 70 DOF and then goes to 0.

The potential for transmission was allowed to vary during the feeding period. It was mediated by a calibrated ‘stress multiplier’, a dynamic parameter responsive to the temporal distribution of BRD events at the population level. The per-day contact rate was adjusted to reflect the increased likelihood of contagious transmission when animals were physiologically stressed and shedding higher numbers of respiratory pathogens (52). The multiplier’s effect was governed by a step function derived from empirical cumulative incidence data (Figure 5) and the cluster analysis in Babcock et al. (53). The combined impact of the calibrated stress multiplier and step function could vary from zero (i.e., no incremental effect on the per-day contact rate) to some positive increment corresponding to the product of the stress multiplier and DOF-determined step function value, added to the per-day contact rate.

#### Treatment agent and state chart

Each treatment agent governed the delivery of the appropriate AMU protocol to an individual animal (Supplementary Figure S9). For all metaphylactic and majority of therapeutic indications, the antimicrobial was administered in a single injectable dose. For all prophylactic (i.e., in-feed) indications and treatments for arthritis, the antimicrobial was administered in a multiday regimen (Supplementary Figures S4, S6). During therapy (i.e., the ‘dosing period’) and the subsequent therapeutic interval (see Table 2), the selection probability was active. It could lead to detectable resistance in the population of *M. haemolytica* for the treated animal. After the therapeutic interval had elapsed following a final dose in a regimen, an ‘end treatment’ message was sent to the resistance agent for the treated calf. The message triggered the transition from ‘stable’ to ‘unstable’ resistance (see ‘Resistance Agent’ section), such that detectable resistance was eligible to wane. Treatment agents were deleted after the withdrawal period (54) for the associated antimicrobial had expired for that animal (Table 2).

### Randomness and stochasticity

Cattle entered the feedlot as part of a stochastic process, with a variable number of animals arriving and filling each pen within 2 days at varying intervals between October and December. Other examples of randomness and stochastics at model initialization included the random assignment of (1) arrival weight (from a uniform distribution); (2) rate of ADG (from a normal distribution); and (3) probability of detectable resistance to each antimicrobial class (from a project evaluation and review techniques [PERT] distribution) to incoming lightweight steers. At initialization, the target market weight for healthy animals (i.e., calves not in the chronic pen) was also randomly selected from a uniform distribution. The prophylactic and therapeutic AMU protocols for a single realization of the model were randomized across a range of plausible alternatives at the start of the simulation.

The calves in the model became sick with the first occurrences of select diseases at stochastic rates drawn from exponential distributions informed by the empirical data summarized in Figure 1. Treatment failures and associated relapses of BRD and arthritis were similarly random events that occurred with fixed probabilities. BRD treatment failures over the baseline probability are responsive to emergent pen-level AMR and are, therefore, stochastic processes (Table 1). AMR acquisition and loss events were simulated stochastically per the calibrated selection and waning rates, respectively (Table 3), and depended partly on emergent patterns of prophylactic and therapeutic AMU. Contagious transmission events were stochastic processes arising from (1) the calibrated contact rates (Table 3) and (2) random contacts between calves in the same pen with discordant resistance status.

**Table 3:** Best objective and calibrated parameter values from individual calibration experiments for each of the antimicrobial class and configuration combinations.

Antimicrobial class	Reference drug belonging to class^a^	Configuration	Best objective value^b^	Selection probability, if applicable	Waning rate	Contact rate, if applicable	Stress effect multiplier, if applicable
				Mean probability per day	Mean rate per day	Mean rate per day	Unitless
15-membered ring macrolides	Tulathromycin	Drug use only	0.213	0.593	0.008	–	–
		Transmission only	**0.157**	**–**	**0.173**	**0.130**	**0.438**
		Both drug use/ transmission	0.186	0.625	1.118	1.163	0.119
16-membered ring macrolides	Tilmicosin	Drug use only	0.592	0.763	0.001	–	–
		Transmission only	0.391	–	1.449	1.600	0.026
		Both drug use/ transmission	**0.382**	**1.000**	**0.825**	**0.915**	**0.026**
Sulphonamides	Sulphadimethoxine	Drug use only	1.725	0.955	0.001	–	–
		Transmission only	0.154	–	2.113	1.299	0.685
		Both drug use/ transmission	**0.153**	**0.452**	**3.393**	**1.982**	**0.741**
Trimethoprim	Trimethoprim	Drug use only	0.039	1	0.013	–	–
		Transmission only	0.018	–	0.035	0.004	3.150
		Both drug use/ transmission	**0.017**	**0.829**	**0.058**	**0.026**	**0.459**
Tetracyclines	Oxytetracycline	Drug use only	0.337	0.134	0.005	–	–
		Transmission only	**0.291**	**–**	**0.011**	**0.005**	**0.623**
		Both drug use/ transmission	0.299	0.011	0.007	0.002	0.940
Cephalosporins	Ceftiofur	Both drug use/ transmission	0.001	0.001	2.763	0.379	0.667
Fluoroquinolones	Enrofloxacin	Both drug use/ transmission	0.002	0.025	0.610	0.001	0.046
Phenicols	Florfenicol	Both drug use/ transmission	0.007	0.002	0.014	0.001	3.038
Fixed minimum value for calibration search function				0.001	0.001/day	0.001/day	0
Fixed maximum value for calibration search function				1	5/day	2/day	10

The configuration with the lowest best objective value for each antimicrobial class (shaded blue) offers the best fit to the empirical data. The calibrated parameter values associated with that configuration are in bold text.

a The antimicrobial drug with the most complete dataset (i.e., one or more raw data points for each time point) was selected to represent the entire class.

b The best (i.e., smallest) objective value was an average of the objective values from each realization (*n* = 30) in the best iteration.

### Input data

Model inputs are displayed in Table 1. Parameter inputs were partly informed by the peer-reviewed literature, market analyses, and expert opinion via consultations with feedlot veterinarians; in particular, the disease and mortality hazard rates were informed by proprietary data from private feedlot operations representing over 1.5 million animals. In the absence of a relevant source, inputs deriving from simplifying assumptions were favoured for the baseline and calibration experiments (identified as ‘model parsimony’ in Table 1). The inputs in Table 1 are organized by subheading to better highlight the agent or state chart where the value is used; the condition(s) precipitating the use of particular values are similarly detailed in the table.

The antimicrobial drugs listed in Table 2 include those available in the model and those to which acquired resistance is of particular interest. Where relevant, the table notes if the drug was used for prophylaxis, metaphylaxis, or therapy in the model, and applications are fully detailed in Supplementary Figures S3–S7. The AMU options are common in western Canadian feedlot medicine, and were developed in consultation with feedlot experts that included the teams which provided the model’s treatment rate data.

Peer-reviewed or other reliable data were unavailable to estimate the duration of selective pressure following treatment with an antimicrobial (i.e., the ‘therapeutic interval’ in Table 2). Elimination half-lives were therefore used to estimate the time required for the drug’s activity to be limited by its decreasing concentration (55–64). Three half-lives (i.e., when 88% of the drug was expected to be eliminated from the animal) were used as a crude estimate of the therapeutic interval. The interval for tulathromycin in the baseline model (8.3 days) corresponds to the midpoint of the plausible values proposed by Brault et al. (65) for that drug’s duration of effect. This assumption was subject to a sensitivity analysis as part of model validation.

Incidence time series for first cases of BRD, arthritis, and foot rot, as well as mortality due to BRD, histophilosis, and all other causes, are loaded from an external file. These are represented as daily time series for a single 1-year feeding cycle, the simulation time for all scenarios examining AMR in *M. haemolytica.*

### Key model outputs

The primary emergent model outputs were the simulated prevalences of detectable resistance to select classes of antimicrobial drugs, arising from the combination of initial conditions, temporal trends in feedlot disease, and prophylactic and therapeutic treatment selections. The resistance prevalence for each antimicrobial class was reported for several time points over the feeding period selected to coincide with those in the reference dataset (see ‘Reference Data Extraction’ section). Related outputs emerging from the model *for each antimicrobial class* and at each time point included (1) the cumulative number of uses of antimicrobials belonging to that class; (2) the cumulative number of acquired resistance events due to selection; and (3) the cumulative number of acquired resistance events due to transmission. For configurations of the model where both AMU-linked selection and transmission could impact detectable AMR, the percentages of resistance acquisition events owing to each route were calculated from these values.

### Model verification

Opportunities to verify that the model was performing as expected were built into both its single- and multirun configurations (26). In the single-run configuration, the model opens a graphical representation of the feedlot with colours and patterns that indicate the state of the pen- and calf-level agents (see ‘Agents and State Charts’ section); this depiction enables the user to visually confirm that the agents are behaving as intended during the simulation. Similarly, the model displays dynamic graphs for single runs that update each model ‘day’ and permit observing how variables of interest are advancing in real time.

Metrics were output to an MS Excel workbook at the end of a model run (i.e., after the simulated feeding cycle). In addition to the *Key model outputs* described above, these included summaries of animal growth (e.g., mean DOF, mean arrival and finishing weights), AMU (e.g., counts of prophylactic regimen applications), disease and mortality (e.g., counts of first and subsequent instances of disease), and specialty pen use (e.g., calf-days in hospital and chronic pens). Together with the visualizations, these outputs were critical for checking and/or troubleshooting the model’s logic during its step-wise construction. A reduced set of summary outputs is generated for multirun configurations, including calibration and Monte Carlo simulations; depending on the desired unit of analysis, each row in the summary table represents a single *pen* or *feedlot* from one model realization.

Expert input and feedback were acquired from feedlot veterinarians over several phases of model development. Long-term engagement with the anticipated users of this model was important for building stakeholder confidence in the model’s utility as a reasonable approximation of a typical feedlot.

### Model calibration

The unknown parameters were estimated by calibrating the model to previously reported resistance data for each antimicrobial class (see ‘Model Calibration’ section and Figure 4 for an overview of calibrated parameters). Calibration refers to the systematic estimation of static input values that minimize the dissimilarity between the model’s emergent behaviour (i.e., the time-varying prevalence of AMR across the feeding period) and the observed or empirical data that serve as calibration ‘targets’ (25). Automated calibration experiments using the widely implemented OptQuest global optimization routine were created in the commercial software program (AnyLogic version 8.8.6: AnyLogic North America, LLC, Chicago, Illinois, USA, release date December 18, 2023) (66–68). Key parameters linked to the emergence and interanimal spread of resistance on feedlots were estimated for distinct configurations of the baseline model.

#### Reference data extraction

A rapid literature search was performed to identify relevant sources of resistance prevalence data for *M. haemolytica* isolates from healthy feedlot calves at various points across the feeding period. Studies concerning samples from primarily sick or dead cattle were excluded, given that these animals were more likely to have been treated with multiple classes or courses of antimicrobials and were not representative of the general feedlot population. Raw data from pertinent studies of western Canadian feedlot cattle treated metaphylactically per industry practice (38–44) were extracted to a spreadsheet in MS Excel. They included the average DOF at sample time, the total number of tested isolates, and the percentage of phenotypically resistant isolates for each antimicrobial of interest.

Data points clustered closely in time were grouped into DOF ranges that best defined the unique phases of the feeding period. Prevalence data from isolates collected at feedlot arrival were classified as occurring at 1 DOF; the DOF for subsequent time points were selected to (1) coincide with a historical reference dataset (40) and DOF relevant to management events in the feeding period or (2) reflect the midpoint of the DOF range in the reported data.

#### Reference data synthesis

**Figure 6:**
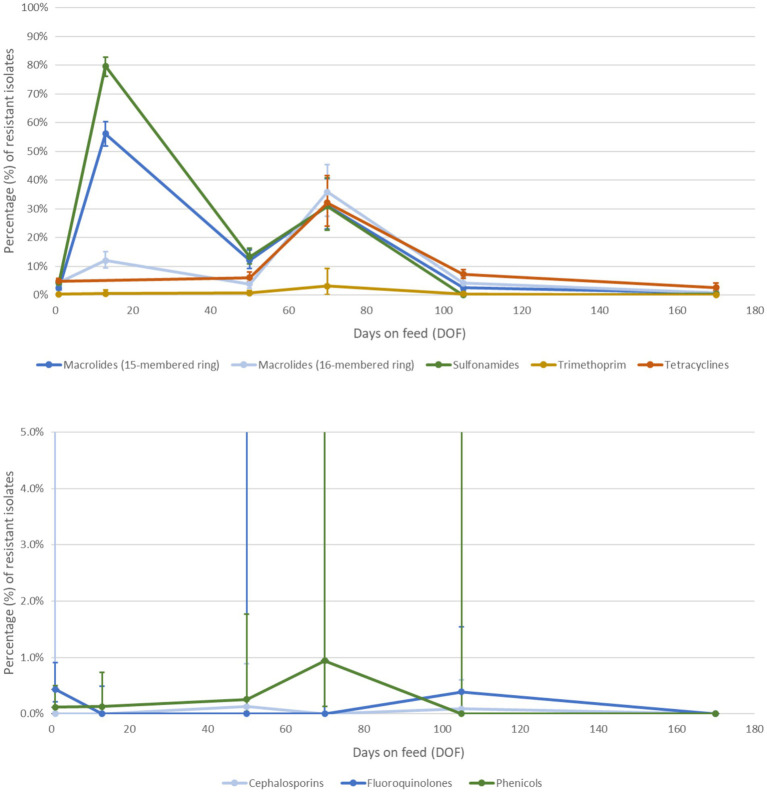
Percentage of *Mannheimia haemolytica* isolates with detectable phenotypic resistance to antimicrobial classes of interest **(A)** selected as external targets against which to calibrate the model and **(B)** not selected for calibration over the feeding period. Antimicrobial classes were selected for calibration if the prevalence of detectable resistance was >1% at any time point and the class was relevant for antimicrobial use in feedlot medicine (70). Each class was represented by AMR to one drug (see Table 3) with the most complete reference dataset. The custom longitudinal datasets were synthesized from recent empirical studies of antimicrobial resistance prevalence in western Canadian feedlot cattle from mixed origins. The percentage of resistant isolates at each time point is a weighted average of prevalence values extracted from recent studies of healthy feedlot cattle in Western Canada (38–44).

A custom longitudinal dataset with updated phenotypic resistance prevalence values was synthesized from the extracted data from all sources for *M. haemolytica* (Figures 6A,B). Because antimicrobial drugs from the same class or subclass were assumed to be equally vulnerable to the relevant resistance mechanism, the drug with the most complete dataset (i.e., one or more data points for each time point) was selected to represent all others in its class. Prevalence and exact 95% confidence intervals were estimated using an intercept-only (or null) generalized estimating equations model in SAS version 9.4 (69) with a binary outcome, binomial distribution and logit link function for each antimicrobial class of interest at each time point (1, 13, 50, 70, 105, and 170 DOF), accounting for clustering by study with a repeated term and exchangeable covariance structure. If the model did not converge, exact confidence intervals were determined using the Clopper-Pearson estimation method. The percentage of resistant isolates at each time point was an average of the raw data values available for that range, weighted by the total number of isolates tested. A prevalence estimate was unavailable for the tetracycline class at 13 DOF; in-feed chlortetracycline was not provided to the animals in the only study with data from that time point (38), and the level of detectable tetracycline resistance (<1%) was an outlier among comparable studies.

#### Calibration configurations

Three model variants with unique structural configurations were each calibrated to the reference data for *M. haemolytica* and subsequently compared. In the first ‘antimicrobial use only’ configuration, the potential for transmission between animals was disabled and could not affect the spread of detectable resistance; selection pressure from AMU was therefore the only driver for AMR in this scenario. The selection probability and waning rate parameters were varied in this configuration to find the best fit to the reference data. In the second ‘transmission only’ configuration, the potential for selection due to AMU was disabled and could not affect the emergence of detectable resistance; contagious transmission was therefore the only driver for AMR in this scenario. The waning rate, respiratory contact rate and stress effect multiplier parameters were varied in this configuration to find the best fit. In the final ‘both antimicrobial use and transmission’ configuration, both drivers were enabled and could jointly affect the emergence of detectable resistance. The selection probability, waning rate, contact rate, and stress effect multiplier parameters were varied in this configuration to find the best fit.

Individual calibration experiments were performed for each configuration and antimicrobial class combination. Antimicrobial classes were selected for calibration if (1) the prevalence of detectable resistance was >1% at any of the time points and (2) the class was relevant to products used in feedlot medicine (e.g., tetracyclines) (70). The 1% threshold was used to distinguish antimicrobial classes where the change in detectable resistance over time was sufficient to model an association with AMU or contagious transmission. The 15- and 16-membered ring macrolides were treated as distinct/independent subclasses given that their reference datasets were sufficiently dissimilar. Resistance breakpoints approved by the Clinical and Laboratory Standards Institute (71) (Wayne, Pennsylvania, USA) did not exist for two of the representative drugs selected for calibration, trimethoprim and sulphadimethoxine. ‘Resistance’, as defined for these drugs, refers to the percentage of isolates that grow at (i.e., are not inhibited by or are not susceptible to) the only tested concentrations (2 and 256 μg/mL, respectively) on the commercially available Bovine BOPO7F AST plate: SensititreTM Vet Bovine BOPO7F Plate, Thermo Fisher Scientific TM, Waltham, Massachusetts, USA (ThermoFisher Scientific™).

#### Calibration settings and objective function

The initialization and simulation settings for each calibration are detailed in Supplementary Table S1. Automated calibration experiments using OptQuest’s optimization tool were run in AnyLogic^®^ 8 for *n* = 2,500 iterations (model runs with unique combinations of target parameters) with *n* = 30 realizations (model runs that explore the extent of stochastic variation within each parameter set) per iteration. The number of iterations and realizations per iteration were selected to balance the competing priorities of methodological rigour (i.e., adequate assessment of stochastic variation influencing the objective function for a specific vector of parameters) and computational efficiency to allow acceptable exploration of the parameter space.

Each calibration experiment (*n* = 15 initial combinations of 5 antimicrobial classes by 3 configurations) searched the parameter space defined by the minimum and maximum values in Table 3 for the inputs that best reproduced the reference data. The best fit was achieved when the average of the objective functions across the realizations within an iteration returned its lowest non-negative value (i.e., when the optimizer minimized the difference between the simulated and empirical data). The compound objective function employed in this calibration comprised two components: (1) the curve fitness component, which applied AnyLogic’s difference function and (2) the point fitness component, which assigned greater weight or importance to reference points with stronger empirical data. The resulting fit at 1, 13 (if available), 70, and 170 DOF was assigned additional weight relative to 50 or 105 DOF given the recency and relevance of the data informing reference estimates for those time points.

#### Monte Carlo experiments

Using the calibrated parameter values from the best iteration for each configuration/drug class combination (see Table 3), Monte Carlo experiments were performed to obtain simulated outputs for the expected prevalence of detectable resistance at time points that matched those from the reference dataset. Each experiment was run for *n* = 5,000 iterations over 1 year; the settings for these simulations were identical to those described in Supplementary Table S1 for the calibration experiments, except that the number of home pens was increased to 48 (*n* = 50 total pens). In particular, all baseline experiments assumed that every calf received tulathromycin at feedlot entry and that metaphylactic and therapeutic success over the feeding period was not impacted by resistance at the calf and pen levels, respectively. Historical first-case hazard and subsequent retreatment rates were expected to reflect the prevalence of AMR in the empirical data used for calibration. Simulated outputs were generated for the *pen* and *feedlot* levels (i.e., agents). Subsequent model sensitivity analyses were built on the foundation of these initial experiments.

### Analysis of model output

#### Pairwise comparisons of best objective values

The objective value for each iteration is an average of the objective values from each realization in the iteration (*n* = 30). The calibrated parameters associated with the best iteration (i.e., the iteration with the smallest objective value) for each configuration/drug class combination are reported in Table 3. The relative fits of candidate configurations (antimicrobial use only vs. transmission only vs. both antimicrobial use and transmission) for each antimicrobial class were evaluated by dividing the absolute differences between the objective values for each pair by the average for each pair. Percentage differences over 20% indicated a substantial difference in model fit per the assessment criteria (72). The results are reported in a difference matrix.

#### Comparison of model fit from simulated outputs

For each configuration/drug class combination, the simulated prevalence of detectable resistance at select time points across the feeding period was summarized with the median and 95% prediction interval (2.5th and 97.5th percentiles) of 5,000 iterations and graphed in R version 4.3.2: The R Foundation for Statistical Computing, Vienna, Austria, release date October 31, 2023 (73). These figures facilitated the relative comparisons of candidate model configurations (i.e., hypotheses about how AMR emerges and spreads in the feedlot environment) against the reference dataset. Simulated data for the *pen* and *feedlot* levels were displayed in the same figure to highlight potential differences in output and model fit by hierarchical unit.

#### Sensitivity analyses

Additional scenarios were examined as part of a sensitivity analysis to evaluate the impact of key assumptions on the simulated prevalences of detectable resistance and relative fit of candidate configurations. The first experiment assessed the sensitivity of model outputs to variation in the therapeutic interval (i.e., the duration of selective pressure following antimicrobial treatment), a parameter estimated to equal three elimination half-lives in the absence of empirical data (55–64). For each of the ‘antimicrobial use only’ and ‘both’ configurations, outputs were simulated for scenarios in which (1) the therapeutic interval for each antimicrobial class in Table 2 was reduced by half; *or* (2) the therapeutic interval for each antimicrobial class was doubled.

A subsequent analysis assessed the impact on the outputs of assumptions in the baseline (calibration) version necessitated by the availability of empirical data for parameterization. Outputs for the ‘antimicrobial use only’ and ‘both’ configurations were simulated for scenarios in which (1) the choice of metaphylactic drug was permitted to vary per the probabilities in Supplementary Figure S3; or (2) the presence of detectable AMR at the calf and pen levels *dynamically* impacted metaphylactic and therapeutic success, respectively, resulting in first treatment and re-treatment rates for BRD over baseline (see ‘Health Status State Chart’ section for details on how *AMR responsiveness* is operationalized for individual calves in the model). A closer investigation of AMR responsiveness was pursued in a follow-up thought experiment that simulated an ‘extreme 15-membered ring macrolide use’ scenario for the same configurations; in this scenario, calves received tulathromycin and gamithromycin for metaphylaxis and all BRD treatments, respectively. These conditions (i.e., repeated 15-ring macrolide exposures in calves with BRD) were intentionally selected to highlight the effect of AMR responsiveness on the likelihood of treatment failure in this model, which might differ from observations in experimental trials (74).

A final analysis assessed the sensitivity of model outputs to variation in the form of step function that directed the impact of the stress multiplier on the baseline contact rate (Figure 5). For each of the ‘transmission only’ and ‘both’ configurations in this analysis, outputs were simulated for scenarios in which (1) the impact of the multiplier on the contact rate was *minimized* by constraining the full strength of the function (=10) to only 21 DOF (11, 75) or (2) the impact of the multiplier on the contact rate was *maximized* by maintaining the full strength of the function through to 70 DOF (76). All sensitivity experiments were run for 5,000 iterations with the calibrated parameter values, and the simulated prevalences of resistance for both *pen* and *feedlot* levels were reported with medians and 95% prediction intervals to demonstrate any differences in the trends across the baseline configurations under modified assumptions.

## Results

### Model calibration

Based on their detectable prevalence in the reference data (>1%) (Figures 6A,B), the antimicrobial classes chosen for the initial calibration experiments were macrolides (15- and 16-membered ring), sulphonamides, trimethoprim, and tetracyclines. The best objective values from the three configurations for the selected drug classes were compared in Table 4. For each of the antimicrobial classes, the ‘transmission only’ and ‘both’ configurations offer a substantially better fit to the empirical data than the ‘antimicrobial use only’ variation. The percentage differences were > 20% in almost every case, with few exceptions; differences for ‘both’ vs. ‘drug use only’ for 15-membered ring macrolides and for ‘both’ and ‘transmission only’ vs. ‘drug use only’ for tetracyclines did not reach the 20% threshold. The differences between the best objective values for the ‘transmission only’ and ‘both’ configurations were less than substantial (<20%) for all classes.

**Table 4:** Pairwise comparisons of best objective values from candidate configurations for each antimicrobial class.

Antimicrobial class	Configuration	15-membered ring macrolides			16-membered ring macrolides			Sulphonamides			Trimethoprim			Tetracyclines		
		Drug use	Transmission	Both	Drug use	Transmission	Both	Drug use	Transmission	Both	Drug use	Transmission	Both	Drug use	Transmission	Both
15-membered ring macrolides	Drug use		**30.3%**	13.5%												
	Transmission	**−30.3%**		−16.9%												
	Both	−13.5%	16.9%													
16-membered ring macrolides	Drug use					**40.9%**	**43.1%**									
	Transmission				**−40.9%**		2.3%									
	Both				**−43.1%**	−2.3%										
Sulphonamides	Drug use								**167%**	**167%**						
	Transmission						**−167%**			0.6%						
	Both						**−167%**		−0.6%							
Trimethoprim	Drug use											**72.4%**	**78.6%**			
	Transmission									**−72.4%**			5.6%			
	Both									**−78.6%**		−5.6%				
Tetracyclines	Drug use														14.6%	11.9%
	Transmission													−14.6%		−2.7%
	Both													−11.9%	2.7%	

The absolute difference between two objective values is divided by the average of those values to compare the ability of candidate configurations to reproduce the reference data. Percentage differences exceeding 20% (shaded blue and in bold text) indicate a substantial difference in model fit.

Based on these findings, single calibration experiments for the ‘both’ configuration were performed to estimate unknown parameters for antimicrobial classes with prevalences of resistance that did not exceed 1% in the empirical data (Figure 6B), but are nevertheless important in feedlot medicine. Parameters for cephalosporins (ceftiofur), fluoroquinolones (enrofloxacin), and phenicols (florfenicol) are reported in Table 3.

### Monte Carlo experiments

**Figure 7:**
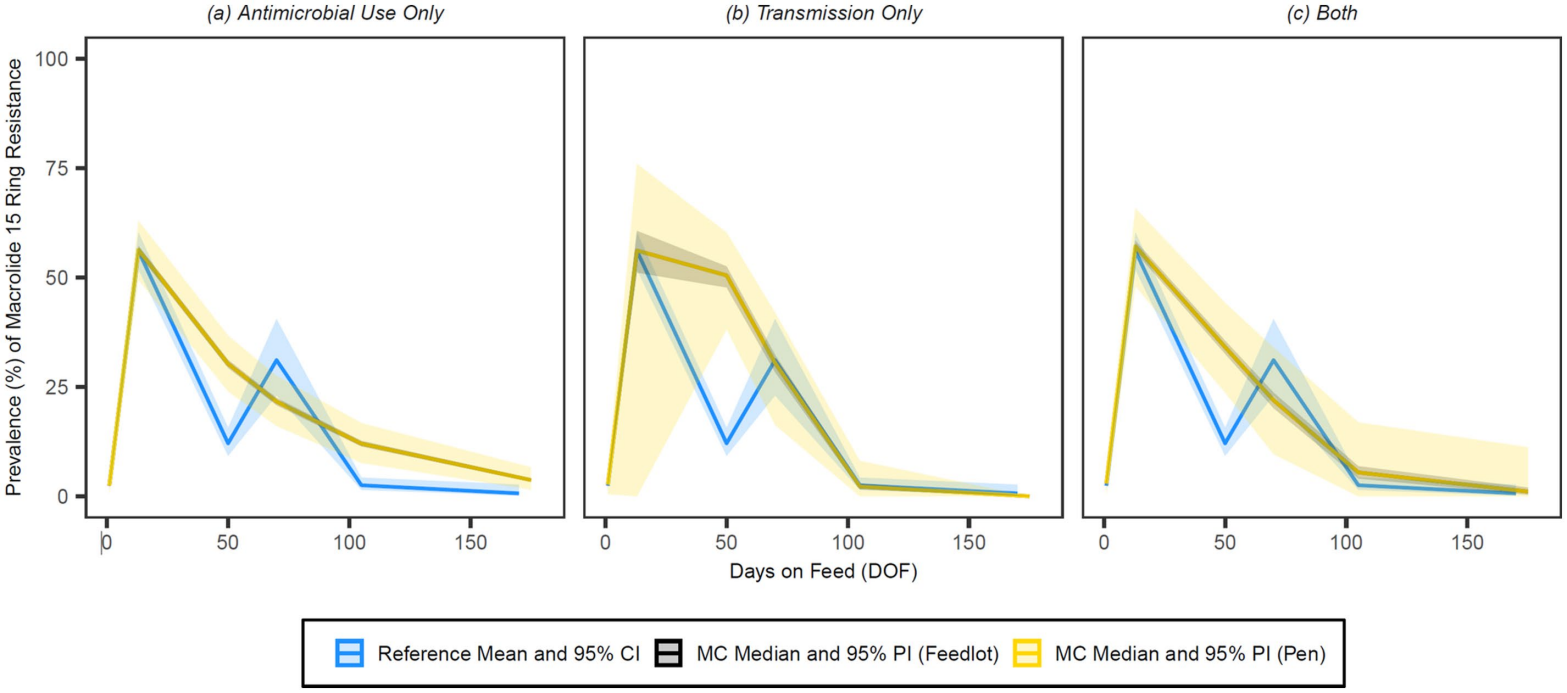
Comparison of model fit for each of the **(A)** antimicrobial use only, **(B)** transmission only, and **(C)** both configurations for 15-membered ring macrolides. Figures depict the range of likely outcomes at the pen (yellow) and feedlot (grey) levels derived from the repeated random sampling of model inputs across 5,000 Monte Carlo simulations. The best objective values for the ‘antimicrobial use only’, ‘transmission only’, and ‘both’ configurations for 15-membered ring macrolides are 0.213, 0.157, and 0.186, respectively. Per the calibration algorithm, a 15-membered ring macrolide (i.e., tulathromycin) is administered to all high-risk cattle entering the feedlot. By 170 DOF, the median number of uses of 15-membered ring macrolide class drugs in the ‘antimicrobial use only’ configuration is,9,600 (range 9,596–9,600); the median cumulative percentage (%) of resistance acquisition events in the ‘both’ configuration attributed to (1) antimicrobial use and (2) transmission is 4.6 and 95.4%, respectively.

**Figure 8:**
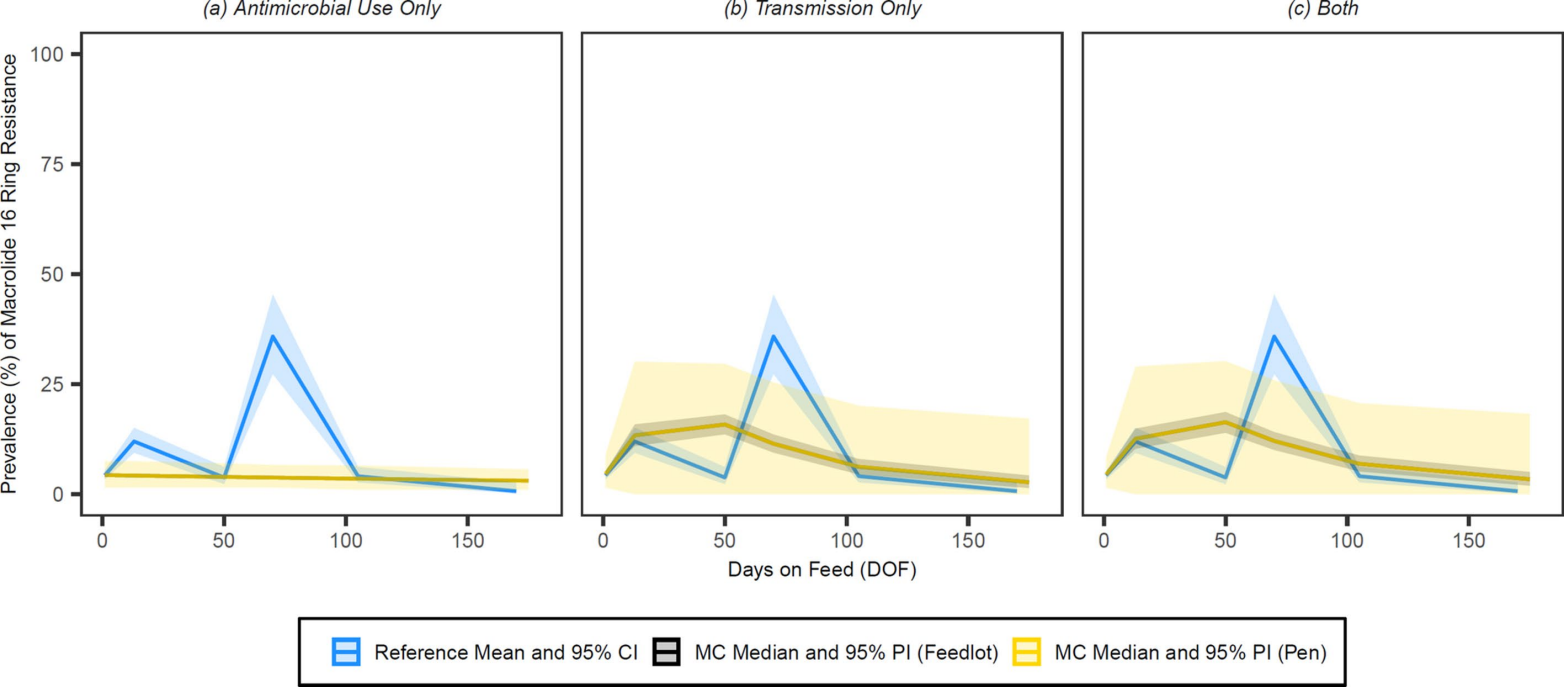
Comparison of model fit for each of the **(A)** antimicrobial use only, **(B)** transmission only, and **(C)** both configurations for 16-membered ring macrolides. Figures depict the range of likely outcomes at the pen (yellow) and feedlot (grey) levels derived from the repeated random sampling of model inputs across 5,000 Monte Carlo simulations. The best objective values for the ‘antimicrobial use only’, ‘transmission only’, and ‘both’ configurations for 16-membered ring macrolides are 0.592, 0.391, and 0.382, respectively. Per the calibration algorithm, 16-membered ring macrolides are not administered metaphylactically or therapeutically to high-risk cattle entering the feedlot. By 170 DOF, the median number of uses of 16-membered ring macrolide class drugs in the ‘antimicrobial use only’ scenario is 0.

**Figure 9:**
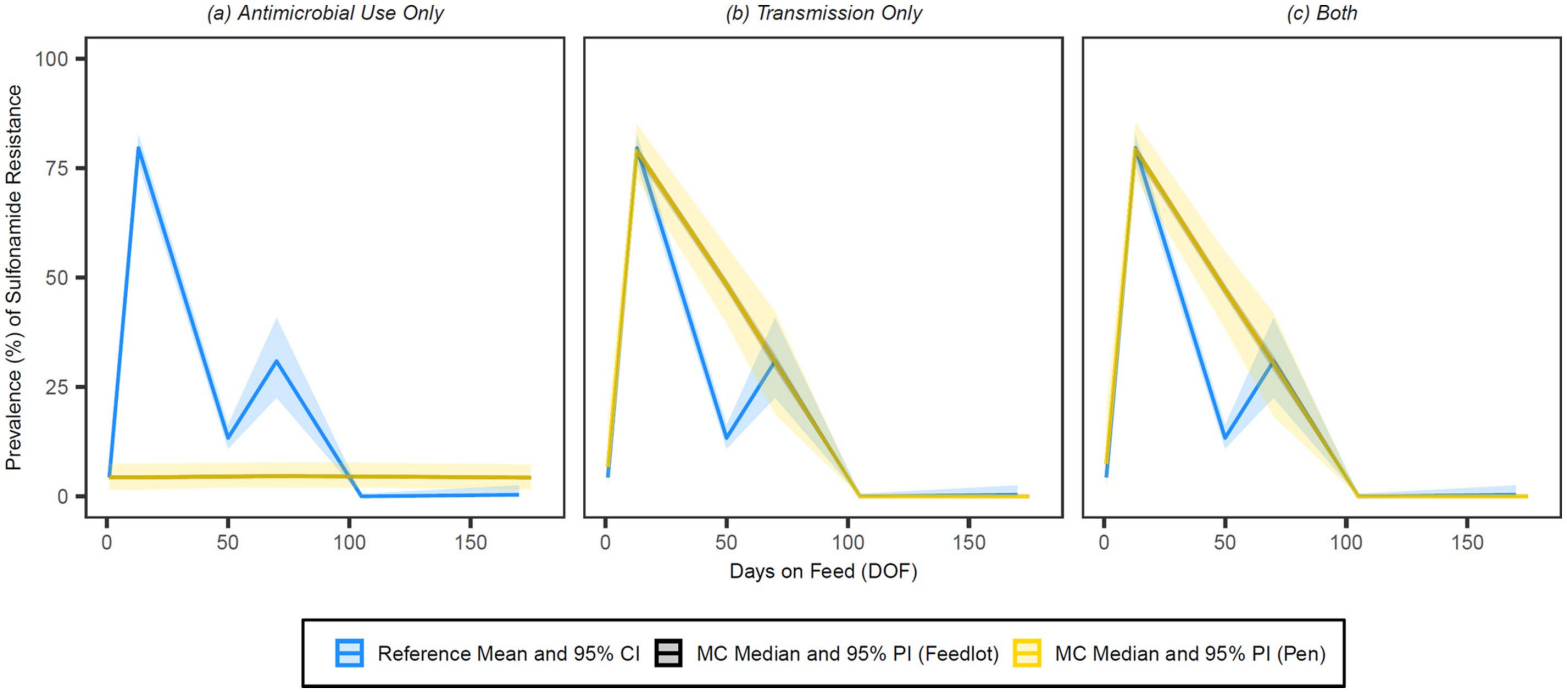
Comparison of model fit for each of the **(A)** antimicrobial use only, **(B)** transmission only, and **(C)** both configurations for sulphonamides. Figures depict the range of likely outcomes at the pen (yellow) and feedlot (grey) levels derived from the repeated random sampling of model inputs across 5,000 Monte Carlo simulations. The best objective values for the ‘antimicrobial use only’, ‘transmission only’, and ‘both’ configurations for sulphonamides are 1.725, 0.154 and 0.153, respectively. Per the calibration algorithm, sulphonamides are administered therapeutically in combination with trimethoprim both (1) to treat second relapses of BRD in cattle under 1,200 pounds and (2) to treat first relapses of arthritis in cattle under 1,000 pounds. By 170 DOF, the median number of uses of sulphonamide class drugs in the ‘antimicrobial use only’ configuration is 103 (range 68–147); the median cumulative percentage (%) of resistance acquisition events in the ‘both’ configuration attributed to (1) antimicrobial use and (2) transmission is 0.02 and 99.98%, respectively.

**Figure 10:**
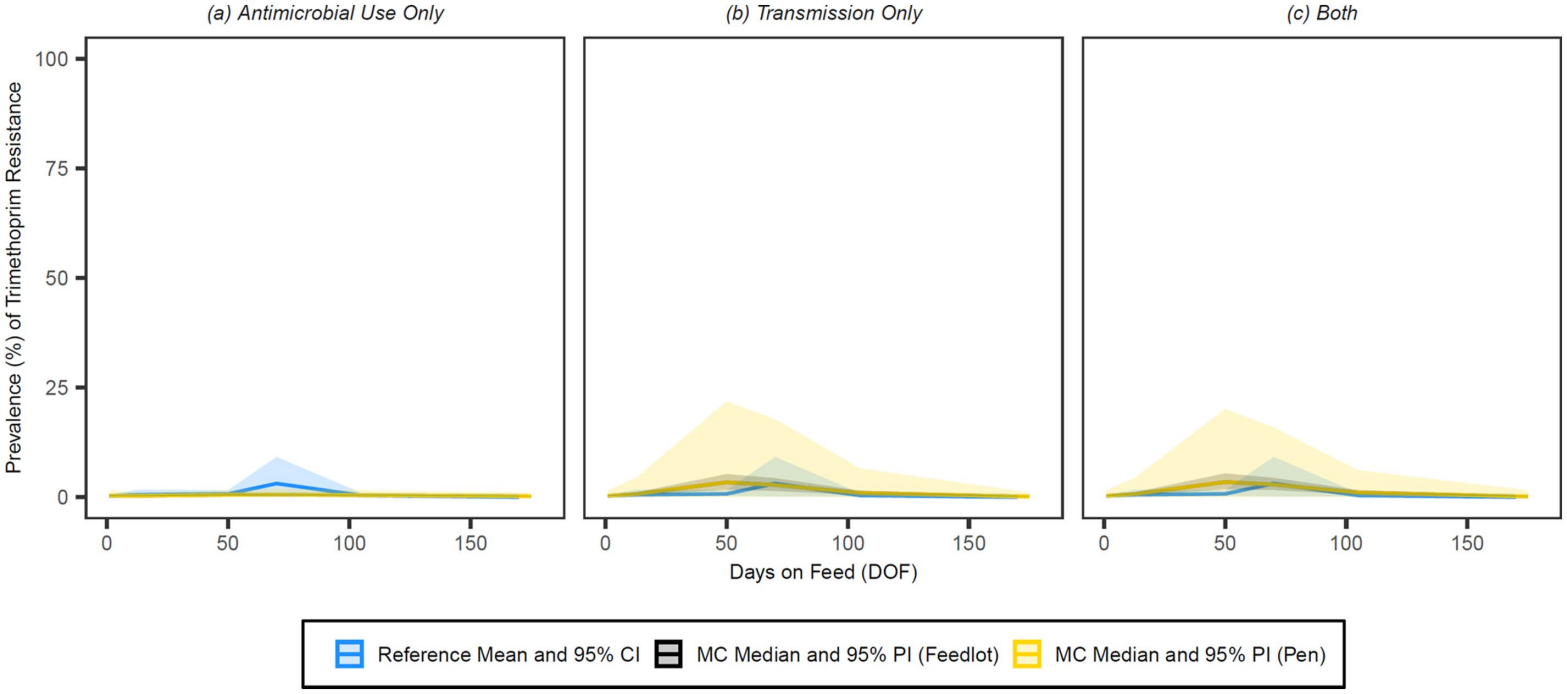
Comparison of model fit for each of the **(A)** antimicrobial use only, **(B)** transmission only, and **(C)** both configurations for trimethoprim. Figures depict the range of likely outcomes at the pen (yellow) and feedlot (grey) levels derived from the repeated random sampling of model inputs across 5,000 Monte Carlo simulations. The best objective values for the ‘antimicrobial use only’, ‘transmission only’, and ‘both’ configurations for trimethoprim are 0.039, 0.018, and 0.017, respectively. Per the calibration algorithm, trimethoprim is administered therapeutically in combination with sulphadoxine both (1) to treat second relapses of BRD in cattle under 1,200 pounds and (2) to treat first relapses of arthritis in cattle under 1,000 pounds. By 170 DOF, the median number of uses of trimethoprim in the ‘antimicrobial use only’ configuration is 103 (range 68–147); the median cumulative percentage (%) of resistance acquisition Events in the ‘both’ configuration attributed to (1) antimicrobial use and (2) transmission is 5.2 and 94.8%, respectively.

**Figure 11:**
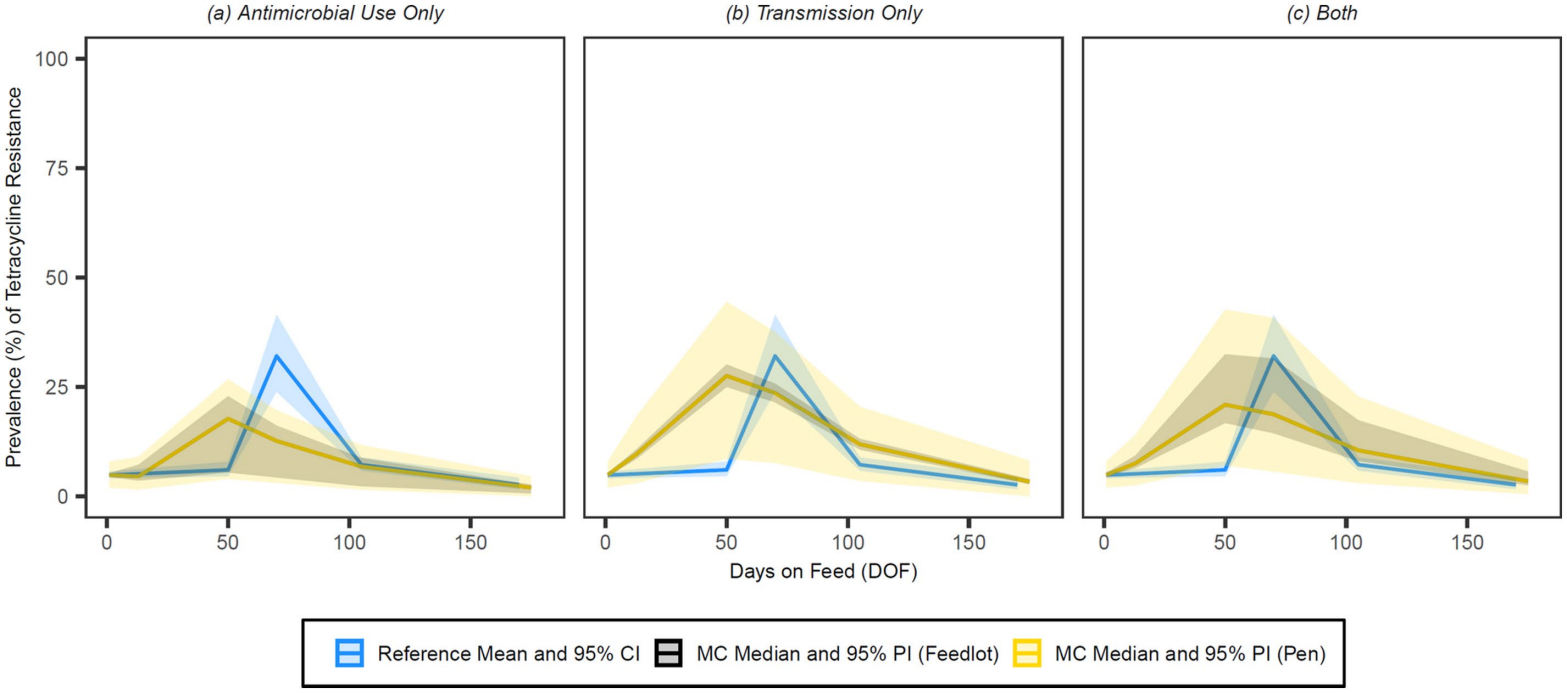
Comparison of model fit for each of the **(A)** antimicrobial use only, **(B)** transmission only and **(C)** both configurations for tetracyclines. Figures depict the range of likely outcomes at the pen (yellow) and feedlot (grey) levels derived from the repeated random sampling of model inputs across 5,000 Monte Carlo simulations. The best objective values for the ‘antimicrobial use only’, ‘transmission only’, and ‘both’ configurations for tetracyclines are 0.337, 0.291, and 0.299, respectively. Per the calibration algorithm, tetracyclines are administered prophylactically (i.e., in-feed) both (1) as a pulse or long-term regimen to prevent histophilosis; and (2) as part of a regimen to prevent liver abscesses in 30% of animals. Tetracyclines are similarly administered therapeutically both (1) to treat foot rot in 50% of cases in cattle under 1,200 pounds and (2) to treat first cases of arthritis in cattle under 1,000 pounds. By 170 DOF, the median number of uses of tetracycline class drugs in the ‘antimicrobial use only’ configuration is 19,292 (range 9,654–28,900); the median cumulative percentage (%) of resistance acquisition events in the ‘both’ configuration attributed to (1) antimicrobial use and (2) transmission is 5.7 and 94.3%, respectively.

The simulated prevalences of detectable resistance across the feeding period were graphed against the reference data for 15-membered ring macrolides (Figure 7), 16-membered ring macrolides (Figure 8), sulphonamides (Figure 9), trimethoprim (Figure 10) and tetracyclines (Figure 11) for each of the three model configurations (labelled A through C in the above).

Visual inspection of the figures confirms that the ‘transmission only’ and ‘both’ configurations have a better fit to the reference data than the ‘antimicrobial use only’ configuration across all classes of interest. Given the weighting scheme applied by the point fitness component of the objective function, model fit at 50 DOF was expectedly poor for the majority of the class/configuration combinations. Injectable 16-membered ring macrolides were not used prophylactically or therapeutically in the baseline version of this model, and thus the ‘antimicrobial use only’ configuration for this class offers a predictably poor fit to the reference data (Figure 8); this is mirrored in the comparatively high objective value for this combination.

All configurations for the 15-membered ring macrolides achieve a similar peak prevalence of resistance (56–57%) and offer a good fit to the reference data at 13 DOF (Figure 7). The prevalence of resistance for the ‘transmission only’ configuration remains high through 50 DOF and declines rapidly after that; the fit of this configuration at the heavily weighted 70 and 105 DOF time points is particularly strong. The ‘antimicrobial use only’ and ‘both’ configurations for this class have more gradual declines than their ‘transmission only’ counterpart, accounting for their better fit at 50 DOF but poorer fits at subsequent time points. At the pen level, the 95% prediction interval (i.e., the variability in the prevalence of resistance) is substantially wider for the ‘transmission only’ than the ‘both’ configuration up to 50 DOF. Conversely, pen-level variation is noticeably wider for the ‘both’ variation after 70 DOF through the end of the feeding period.

The relative fit of the ‘antimicrobial use only’ configuration to the empirical data is stronger for tetracyclines than other antimicrobial classes (Table 4, Figure 11). The simulated outputs for this and the ‘both’ configuration are characterized by wide feedlot-level prediction intervals through 105 DOF; this variability reflects the considerable range (9,654–28,900) in the number of tetracycline uses by 170 DOF due to reported variation in prophylactic protocols for histophilosis and liver abscess prevention (see Supplementary Figure S4). The ‘transmission only’ and ‘both’ configurations for tetracyclines follow similar curves with comparable pen-level variation in outcome reflected in the width of their prediction intervals. Because the ‘transmission only’ configuration for this class reaches a higher peak prevalence of resistance at 50 DOF (27.6%), the model fit is stronger at 70 DOF than for the ‘both’ variation.

By 50 DOF, the median cumulative percentage of resistance acquisition events attributed to transmission in the ‘both’ configuration was >90% for all classes; the percentage of resistance acquisition events attributed to AMU was 6.5% for 15-membered ring macrolides, 4.7% for trimethoprim, 7.7% for tetracyclines, and nil/negligible for sulphonamides. Similarly, the percentage of resistance acquisition events attributed to AMU by 170 DOF was 4.6% for 15-membered ring macrolides, 5.2% for trimethoprim, 5.7% for tetracyclines, and nil/negligible for sulphonamides. Both sets of observations highlight the importance of contagious acquisition of resistance in achieving a good fit to the observed data.

### Sensitivity analyses

The simulated prevalences of resistance at 13, 50, and 70 DOF for all classes/configurations under modified assumptions (i.e., ‘scenarios’) were reported in Supplementary Table S2 (*pen* level) and Supplementary Table S3 (*feedlot* level). In general, the model outputs were more robust to changes in the assumptions underscoring AMU (i.e., therapeutic interval length, variation in metaphylactic drug exposure) and the responsiveness of BRD treatment failure to AMR than to changes in duration of the effect of the stress multiplier on transmission potential.

For 15-membered ring macrolides, outputs for the ‘both’ configuration at 13 DOF were uniquely sensitive to changes in therapeutic interval length; at this time point, doubling the therapeutic interval substantially increased the median prevalence of resistance from 57 to 100% at both the *pen* and *feedlot* levels (Supplementary Tables S2, S3). The increases in prevalence owing to interval length were more modest (3–4%) for the ‘antimicrobial use only’ configuration, highlighting how transmission amplified selective pressure for resistance to macrolides in the early feeding period. When the choice of metaphylactic drug was permitted to vary, the variation in outcome (i.e., the width of the 95% prediction intervals) increased considerably relative to baseline for both configurations and at most time points (Supplementary Tables S2, S3). At the *pen* level, the lower bound of the interval reduced to <1% for all permutations, implying that more simulations generate nil/low prevalences of resistance through 170 DOF when metaphylaxis was selected probabilistically. At the *feedlot* level, increased variation in outcome due to metaphylactic variability had largely disappeared by 50 DOF in the ‘both’ configuration.

For the tetracycline class configurations, absolute differences in the median resistance prevalence in response to AMU variations did not exceed 2% at either level or any time point (Supplementary Tables S2, S3). The variation in outcome for tetracyclines was similarly robust to changes in the choice of product for metaphylactic use, a likely consequence of the existing variability in prophylactic and therapeutic use across simulations in the baseline configurations. Outputs for all the sulphonamide class and trimethoprim configurations were similarly insensitive to changes in the AMU assumptions (no absolute differences >0.5% in the median prevalence of resistance at either level or any time point). These drug classes were only used together therapeutically as potentiated sulphonamide injectables for previous BRD treatment failures and, therefore, infrequently in the configurations where AMU impacts detectable AMR (103 median uses by 170 DOF).

The impact of making BRD treatment failures responsive to AMR was nil/negligible under the baseline treatment protocols (Supplementary Tables S2, S3), suggesting that the emergent prevalence of pen-level resistance to therapeutic drugs rarely exceeds the fixed treatment failure rate derived from historical data. In the ‘extreme macrolide use’ counterfactual scenario, the median prevalence of resistance to 15-membered ring macrolides was reasonably robust to AMR responsiveness for both configurations where AMU could impact detectable resistance and at both *pen* and *feedlot* levels (Supplementary Table S4). However, observed increases in the number of 15-membered ring macrolide uses (Supplementary Table S4) and the number of first and subsequent BRD cases due to AMR-associated failure of metaphylaxis or treatment (Supplementary Table S5) demonstrate the functionality of the AMR responsiveness mechanism.

In the scenarios with modified step functions that either constrained or maximized the impact of stress on the effective contact rate, there were substantial changes in the median prevalences of resistance for the majority of the antimicrobial classes/configurations where transmission impacts detectable AMR (Supplementary Tables S2, S3). For both 15-membered ring macrolides and tetracyclines, the absolute differences in median resistance were greatest for the ‘transmission only’ configuration at 50 and 70 DOF for both *pen* and *feedlot* levels; when the stress multiplier’s effect was limited to the first 21 DOF, resistance at the *feedlot* level by 50 DOF dropped to 5 and 9%, respectively. Conversely, when the stress multiplier’s full effect was maintained to 70 DOF, resistance increased to 74 and 43% by 50 DOF for 15-membered ring macrolides and tetracyclines, respectively, and remained high through 70 DOF. Outputs for the sulphonamide configurations were especially responsive to changes in transmission potential, given the primary role of transmission in AMR emergence for this class.

Changes to the step function had related impacts on the median percentage of resistance acquisition events attributed to AMU at 50 and 170 DOF in the ‘both’ configuration for all classes. When the contact rate multiplier effect is constrained to 21 DOF, there were fewer total acquisition events, and AMU accounts for a greater percentage of these than in the baseline scenario. For 15-membered ring macrolides and tetracyclines, the percentage of resistance acquisition events attributed to AMU by 50 DOF increased to 10.2 and 16.5%, respectively.

## Discussion

While it is well-established that the development of AMR in animal production and elsewhere is accelerated by ‘selection pressure placed on susceptible microbes by the use of antimicrobial agents’ (3), a comprehensive understanding of the factors that cause the spread and persistence of AMR bacteria is limited by the currently available data (77). Knight et al. (78) describe how mathematical models can inform policies for managing AMR but acknowledge that the major drivers of the spread of resistance at the population level have not been ‘convincingly identified’. This study aimed to explore hypotheses about how population-level AMR emerges in a typical, small- to mid-sized Canadian feedlot with calves at increased risk for BRD. The baseline version of the model was exclusively populated by fall-placed steer calves sourced via auction; calves with this risk profile are most often recently weaned and lighter weight animals, and are more likely than heavier and older animals to be in the early stages of respiratory disease (29) and to receive metaphylactic antimicrobials at feedlot entry (6).

Mathematical or dynamic models are well-suited to the representation and investigation of AMR and other complex, adaptive systems (23). Still, there are few examples of this approach in agriculture and food systems settings (24). Available studies are most often of the aggregate or compartmental type and employed to examine the within-host dynamics of AMR in single food animals (79–82); a notable exception extends a compartmental model to study the within-herd spread of resistant *Escherichia coli* in pigs (83). Aggregate models are comparatively easy to parameterize but are limited in their ability to account for individual heterogeneity or the substantial impact of chance events in closed systems (84), including feedlot pens. This novel study draws on the many strengths of agent-based modelling techniques (85), including (1) the explicit modelling of individual animals with unique risk profiles, disease histories, and exposures to AMU; (2) the flexibility to incorporate behavioural units at multiple scales in a nested structure (e.g., each of animal, pen, and feedlot ‘agents’); and (3) the ability to capture emergent system phenomena (e.g., AMR prevalence) arising from the rule-based interactions of agents with each other and their co-evolving environments. This approach has been used effectively elsewhere in research concerning BRD dynamics in the context of French fattening farms. Picault and colleagues investigated the impact of farming practices, including pen size and metaphylaxis, on BRD outcomes and antimicrobial usage in calves of varying risk with a stochastic, individual-based model (86).

Birkegård et al. (87) argue that to extract useful conclusions from advanced mechanistic (i.e., agent-based) models depicting AMR, related research should focus on providing data to parameterize and validate these tools. Notable strengths of this work are its use of diverse, multilevel datasets to explain population-level resistance trends (78) and the transparency with which the inputs are reported (24, 26). This model integrates current and emerging research with publicly available surveillance data, market analysis data (e.g., weight and ADG metrics), and expert opinion informed by feedlot veterinarians (e.g., likelihood of AMU treatment protocols). Exogenous (i.e., externally specified) variables that precipitate exposures to antimicrobials, including the first-case hazard rates for select feedlot diseases (Figure 1), the disease-specific and all-cause mortality rates (Figures 2A,B), and related disease parameters (Table 1), are derived from proprietary data from partner veterinary practices representing over 1.5 million animals from 2007 to 2020. The ‘huge work to parameterize these models’ (87) was undertaken here in a singular effort to solicit setting- and context-specific inputs with which to ground the model in robust epidemiological data.

Martínez and Baquero (88) propose that the ‘emergence and spread of antibiotic resistance can only be understood in a multi-parameter space’ including ecological *selection* and *contact rates*, among others. Calibration is a useful approach to generate parameter values for which estimates are unavailable or otherwise unobservable, as with those that govern changes in detectable AMR (Figure 4). Furthermore, to evaluate the relative importance of plausible hypotheses contributing to resistance spread, it is necessary to systematically calibrate models to empirical data (namely, the time-varying proportion of resistant isolates across the feeding period) (78). Suitable ‘calibration targets’ (25) in the required format were unavailable, and the synthesis of a custom, longitudinal dataset for *M. haemolytica* was a critical component of this study (Figures 6A,B). Access to the dataset described by Noyes et al. (40) was granted by P. Morley (personal communication, 2018), and established a historical baseline prevalence of resistance. In addition to more recent (2019–2021) data from CFAASP, the reference dataset is also populated with empirical resistance data from approximately 1,600 recently weaned fall-placed feedlot calves at several points across the early feeding period (89). The model could theoretically be re-calibrated for other BRD-associated pathogens (16, 34) if suitable longitudinal reference datasets could be curated. The potential to model AMR transmission between bacterial species of interest is a logical extension of this study.

Birkegård et al. (87) note that all the models of AMR development and spread included in their review assert that ‘an increase in antimicrobial use increases AMR’. The results of our study convincingly demonstrate that the emergence of population-level AMR cannot be fully understood without also accounting for transmission in the model’s structure, jointly operationalized here by the calibrated contact rate and stress multiplier parameters. Across all antimicrobial classes of interest, the model performed worse when the impact of transmission on the spread of detectable resistance was ignored (i.e., in the ‘antimicrobial use only’ configurations). Furthermore, the large majority (>90%) of the resistance acquisition events at 50 DOF and through 170 DOF in the ‘both’ configuration was a consequence of *contagious* rather than *selective* acquisition for all antimicrobial classes. Consistent with this finding, Abi Younes et al. (38) report evidence of the rapid interpen spread of a macrolide-resistant *M. haemolytica* clone by 13 DOF in healthy feedlot calves that received metaphylactic tulathromycin. Snyder et al. (90) similarly describe the ‘contagious spread’ of *M. haemolytica* between stocker calves after metaphylaxis as demonstrated by the genetic relatedness (i.e., clonality) of multidrug-resistant isolates collected at revaccination (10–14 days after arrival).

The extent to which ABM permits the strategic incorporation of stochastic elements to describe real-world entities and phenomena was among the many motivations for pursuing this type of representation (91). Indeed, individual models are the preferred tool to account for (1) the biological variability of agents across one or more dimensions and (2) uncertainties deriving from the data used as model inputs. Stochastic processes are a key feature of the model (see *‘*Randomness and Stochasticity’ section), but were constrained in this baseline exploration by assumptions that reflected the availability and generalizability of empirical data for parameterization and calibration. For example, while a probabilistic selection mechanism exists for metaphylactic drug selection (Supplementary Figure S3), this choice was *fixed* in the baseline version (i.e., every calf received tulathromycin at entry, consistent with the AMU history of calves in the reference and BRD incidence data). This and other key assumptions imposed on the system, including those that defined the risk status of incoming animals, effectively limited a more extensive exploration of stochasticity in the model’s outputs. At the *feedlot* level, these restrictions were reflected in the narrow prediction intervals for the majority of the antimicrobial class/configuration combinations in the baseline scenario (Figures 7–11).

The wider prediction intervals for the *pen* level outputs in Figures 7–11 highlight the increased variability in the prevalence of resistance at this unit of analysis. Smaller or subpopulations (e.g., calves from a single ‘home’ pen) are more vulnerable to the impacts of chance events than their superset counterparts (e.g., calves from all pens in the feedlot). Furthermore, there were more repetitions and, therefore, opportunities to explore stochastic combinations at the *pen* level (*n* = 240,000 pens over 5,000 iterations). Increased variability in outcome was similarly observed for select antimicrobial classes when the assumption restricting drug choice was relaxed in the scenario with metaphylactic variation; the outputs for 15-membered ring macrolides were especially sensitive to this change (Supplementary Tables S2, S3), given that the proportion of simulations with universal tulathromycin metaphylaxis use was reduced by over 40% (per the probabilities in Supplementary Figure S3). In general, varying the choice of metaphylactic drug had a greater impact than modifying the stress multiplier’s effect on AMR transmissibility on the variability in outcome (i.e., the *range* of resistance prevalences across realizations of the model) for all classes.

Conversely, the *median* prevalences of resistance for most antimicrobial classes were markedly more sensitive to changes in the step function and adjusted contact rate than to the assumptions related to AMU and AMR responsiveness. The effect of the stress multiplier is constrained to 21 DOF in the minimized function, consistent with the observation that the peak incidence of BRD associated with *M. haemolytica* generally occurs within 2–3 weeks of feedlot arrival (11, 30, 75). In the maximized function, the effect of the stress multiplier is maintained at full strength to 70 DOF to account for more delayed patterns of morbidity timing in some cohorts (92). The extreme variations of the step function explored in the sensitivity analyses are useful experiments but biologically improbable (i.e., the increased likelihood of pathogen transfer owing to physiological stress is unlikely to stop at 21 DOF or to continue without abating to 70 DOF). Furthermore, BRD cases occurring beyond the early feeding period are more likely to be associated with other or mixed BRD pathogens (e.g., *M. bovis* and *H. somni*) than *M. haemolytica* (93, 94). The function in the baseline scenario reflects empirical data for first-case BRD diagnoses in high-risk calves (Figure 5) and the finding elsewhere (53) that ‘75% of BRD cases are reported to occur within the first 40–55 days after arrival’ (76).

Despite having minimal impact on the outputs of interest in this study, the AMR responsiveness mechanism is nevertheless a critical feature of the feedlot model. The conditions required for maximum responsiveness (i.e., high levels of detectable resistance to the therapeutic options) were simulated in the ‘extreme 15-membered ring macrolide use’ scenario, a thought experiment that (1) confirmed the emergent stochastic element was working as intended; and (2) identified how related outputs (e.g., BRD cases and AMU counts) were affected by responsiveness to pen-level AMR. There were 10% more first cases of BRD (owing to metaphylactic failure) and 110% more relapses of BRD (owing to therapeutic failure) in the responsive vs. non-responsive versions of the ‘both’ configuration (Supplementary Table S5). Resistance-linked treatment failure could not be decoupled from other-cause treatment failure in the empirical data available for parameterization, and was, therefore, externally specified in the calibration version of this model. In future experiments, model responsiveness to detectable resistance (informed by testing or otherwise) will allow for increasingly complex feedback between AMU and AMR and more meaningful analysis of truly emergent model properties.

The 15- and 16-membered ring macrolides were treated as distinct subclasses and simulated independently in all model versions reported here. There were several practical and biological reasons for this choice, including that there was nil or negligible *injectable* 16-membered ring macrolide (i.e., tilmicosin and tildipirosin) use in the studies from which the reference data were synthesized (38–44). The confidence in the outputs informed by the data for this subclass was consequently lower than for their 15-membered ring counterparts. Furthermore, substantially dissimilar prevalences of phenotypic resistance to tulathromycin and tilmicosin were observed for isolates across the early feeding period in (38), highlighting the previously described potential for distinct genetic pathways to resistance by macrolide subclass (95, 96). Notably, the newly discovered resistance gene *estT* has been detected in *M. haemolytica* isolates and encodes an enzyme that hydrolyzes 16- but not 14- or 15-membered ring macrolides (97). A series of calibrations in which detectable resistance to 15- and 16-membered ring macrolides is linked with a conditional probability is planned but beyond the scope of this paper. Importantly, *in-feed* tylosin use did not co-select for resistance to macrolides in either subclass in these experiments, given that orally administered macrolides did not impact the prevalence or susceptibility of respiratory microbes in a relevant study from Zaheer and colleagues (45).

The outputs from these simulations cannot be generalized to feedlot systems outside of the western Canadian context or feeding operations with different management practices or risk groups. The parameters governing the acquisition and loss of detectable resistance are calibrated to the unique population of cattle and the set of assumptions adopted here (see ‘Key Assumptions Underscoring Model Conceptualization’ section and Supplementary Table S1). Importantly, these and other parameters can be modified to reflect alternate feedlot and animal characteristics, changing expert opinion, and the conditions specific to *other* reference datasets as they become available. Related limitations of this study similarly deserve consideration. This model assumes that every calf has a commensal population of *M. haemolytica*, and that treatment failures over historical retreatment rates in the ‘AMR-responsive’ scenarios depend on the emergent prevalence of pen-level phenotypic resistance in this organism. While *M. haemolytica* is often regarded as the primary bacterial pathogen associated with acute BRD (37), the presence and impact of AMR in other BRD-associated bacteria, including *P. multocida*, *H. somni*, *and M. bovis* (16, 34), were not considered here. This necessary simplification nevertheless provides an empirical rather than theoretical basis for examining the dynamics of AMR in a specific pathogen [i.e., in contrast to an ‘average’ pathogen, as is sometimes modelled in other studies (e.g., (86))]. Indeed, a previous review (24) noted that resistance was modelled for a generic bacterial organism and/or a generic antimicrobial in 40 and 74% of the models, respectively, that investigated the relationship between AMU and AMR.

This study establishes the feedlot model as a tool that can be used to explore questions related to AMR and antimicrobial stewardship in the context of BRD management. Model variants which included the impact of *contagious* acquisition on population-level AMR offered a stronger fit to empirical data and will be used in forthcoming experiments. This study offers a preliminary quantitative assessment of the relative contributions of AMU-linked selection and transmission to AMR emergence in this setting. It might reasonably inform interventional studies that investigate BRD management strategies in feedlots. The agent-based modelling framework described here is sufficiently flexible to accommodate updates or modifications to the infrastructure as required. Recent additions include laboratory testing that will facilitate the comparison of different pen sampling strategies and AMR diagnostic techniques consistent with the broader goals of this project. The emergent behaviour of AMR across the feeding period may be impacted by the introduction of testing-informed treatment decisions at the pen level (98) and is the focus of future analyses.

## Data Availability

The original contributions presented in the study are included in the article/Supplementary material, further inquiries can be directed to the corresponding author.
