# Supplementary material for: Contagious acquisition of antimicrobial resistance is critical for explaining emergence in western Canadian feedlots—insights from an agent-based modelling tool
Source: Front Vet Sci. 2025 Jan 10;11:1466986. doi: 10.3389/fvets.2024.1466986 (PMC11758982; doi:10.3389/fvets.2024.1466986)
Supplement: Supplementary file 1 [file Data_Sheet_1.pdf]

Supplemental file 1

ODD Protocol: Virtual Feedlot Model

An Agent-Based Simulation Model of AMR Emergence in a Western Canadian Feedlot

Contagious acquisition of antimicrobial resistance is critical for explaining emergence in western Canadian feedlots: Insights from an agent-based modelling tool

Dana Ramsay, Department of Large Animal Clinical Sciences, Western College of Veterinary Medicine, University of Saskatchewan, Saskatoon, SK, Canada

Wade McDonald, Department of Computer Science, University of Saskatchewan, Saskatoon, SK, Canada

Michelle Thompson, Department of Large Animal Clinical Sciences, Western College of Veterinary Medicine, University of Saskatchewan, Saskatoon, SK, Canada

Nathan Erickson, Department of Large Animal Clinical Sciences, Western College of Veterinary Medicine, University of Saskatchewan, Saskatoon, SK, Canada

Sheryl Gow, Canadian Integrated Program for Antimicrobial Resistance Surveillance, Public Health Agency of Canada, Saskatoon, SK, Canada

Nathaniel Osgood, Department of Computer Science, University of Saskatchewan, Saskatoon, SK, Canada

Cheryl Waldner, Department of Large Animal Clinical Sciences, Western College of Veterinary Medicine, University of Saskatchewan, Saskatoon, SK, Canada (cheryl.waldner@usask.ca)

ODD Protocol: Virtual Feedlot Model

An Agent-Based Simulation Model of AMR Emergence in a Western Canadian Feedlot

A stochastic, continuous-time, agent-based model (ABM) was constructed with AnyLogic® 8 simulation software (version 8.8.6) using Java-based code, with the goal of developing a tool that can be used to explore questions related to antimicrobial stewardship in the management of bovine respiratory disease (BRD) in finishing feedlots. This model description follows the ODD (Overview, Design concepts, Details) protocol for describing agent-based models [1], as updated by Grimm *et al.* [2]. The following description will note where the assumptions and/or model parameters can be modified to customize the output for different feedlot sizes, management practices, and cattle characteristics.

Overview

Purpose

The purpose of this work was to explore the dynamics of population-level antimicrobial resistance (AMR) emergence in a typical, small to mid-sized Canadian feedlot (6,000 to 10,000 animals) with calves at increased risk for BRD. While the model framework can be easily modified for feedlots of any size, this configuration was chosen to provide a balance between the size of many commercial feedlots in Canada and computational efficiency for the ABM. The lighter-weight calves in the baseline model are assumed to represent a moderately high risk of AMR selection and dissemination in the feedlot setting, given the potential for infectious disease spread and exposure to antimicrobials [3].

Hypotheses about how AMR emerges and spreads in the feedlot environment are represented by model variations in this baseline effort; structural modifications distinguish between 1) a model in which only selection arising from antimicrobial use (AMU) impacts detectable AMR, 2) a model in which only transmission between animals impacts detectable AMR, and 3) a model in which both AMU-linked selection and transmission impacts detectable AMR. A comparison of the relative performances of these models with respect to reproducing empirical trends in AMR is a key component of this work.

Mannheimia haemolytica is one of several bacterial pathogens implicated in the clinical presentation of BRD, particularly in high-risk calves following feedlot arrival [4, 5]. *M.*

haemolytica was selected as the sentinel organism for AMR in respiratory pathogens, given 1) the broad assumption that the resistance status for *M. haemolytica* is representative of clinically relevant AMR in the nasopharyngeal microbiome, and 2) the availability and reliability of temporal resistance prevalence data for this organism. For the purposes of this model, it is assumed that every calf has a population of *M. haemolytica* existing as nasopharyngeal commensals. For each calf in every pen, the resistance status of *M. haemolytica* to select antimicrobial classes (i.e., the presence or absence of detectable AMR in the population of *M. haemolytica*) is monitored over the course of the feeding period. Population-level AMR can evolve due to selection pressure associated with preventative and therapeutic AMU in the model and/or direct transmission of resistant bacteria between calves in the same pen. The model simulates the development and management of select syndromes that most often trigger injectable (i.e., individually-dosed) AMU in the western Canadian feedlot setting, including BRD, bacterial arthritis and infectious pododermatitis (i.e., foot rot) [6, 7].

Entities, State Variables, and Scales

The system is modeled as a collection of autonomous entities called *agents*. The baseline model describes six types of agents. The agents in the model are structured hierarchically, with pens filled with cattle. Each calf harbours a resistance agent that represents the detectable susceptibility status of their population of *M. haemolytica* to a set of commonly used antimicrobials. The treatment agent represents specific occurrences of AMU in each calf and has the potential to impact resistance status. Each agent is assigned internal states and behaviours encoded by rules which govern the transitions between states over time. Interactions between agents produce emergent, system-level dynamics.

Main

The **Main agent** represents the program entry point and top-level agent for most AnyLogic models; it contains all other agents, any sub-models, any parameters that can be varied for scenarios, and live data visualizations. This model is set up to load parameters and other exogenous data tables from externally editable spreadsheet files. All external data files are called within the Main agent.

Feedlot

The **Feedlot agent** governs the processes of calf arrival, the allocation of calves to pens, and the re-sorting of calves among pens later in the feeding period (if enabled through a modifiable

parameter). The feedlot consists of individual pens each with a cohort of individual calves. The Feedlot agent is responsible for aggregating pen-level statistics and assigning AMU protocols for disease control (**Figures S1-S2**) and treatment (**Figures S3-S5**). AMU protocols can be specified by the user or randomized across a range of common alternatives [6-8] developed in consultation with feedlot practitioners. With either option, the selected protocols are applied to the entire feedlot for one realization of the model.

Pen

The **Pen agents** record the filling and emptying of pens by calf agents, and govern the timing and delivery of antimicrobials for disease *control* (i.e., prophylaxis and metaphylaxis, see **Figure 1**). Management protocols involving antimicrobials and thus the potential for AMR selection included each of 1) on-arrival injectable metaphylaxis for BRD management; 2) in-feed prophylaxis for histophilosis and liver abscess prevention; and 3) in-feed prophylaxis for foot rot outbreak control, when 10% or more calves in a shared pen were diagnosed with foot rot in a single feeding period. The options and/or likelihoods of available metaphylactic and prophylactic protocols in the model are outlined in **Figures S1** and **S2**, respectively. Given that the data available to calibrate the model are largely derived from empirical studies of high-risk calves administered macrolides at feedlot entry [e.g., 9-10], all animals in the baseline scenarios are directed to receive metaphylactic tulathromycin by default.

When the average weight of the animals in a single pen reaches the target finishing weight, calves are assumed to be ready for slaughter. Pens are subsequently de-populated to simulate the shipment of finished calves to a processing plant, and pen summary data are exported. The Pen agent gathers statistics on cattle shipped for processing, including the cumulative incidence of disease and the prevalence of detectable AMR to selected antimicrobial classes at specified time points.

Cattle

Each **Cattle agent** characterizes the health status for a single calf and governs the timing of and response to antimicrobials for disease *treatment*. Healthy calves can become sick with BRD, bacterial arthritis, or foot rot (**Figure 2**), syndromes that are most frequently associated with injectable AMU in the western Canadian feedlot setting [6]. The options and likelihoods of available therapeutic protocols in the model are outlined in **Figures S3-S5**. The number of

treatments for each calf agent is tracked and reported, as is their health status-dependent average daily gain (ADG) and resultant weight.

Figure 1. Process of calf management for each home pen in the agent-based model. Transition arrows demarcated by "clock" symbols are processes linked to a particular length of time (e.g., the number of days on a certain feed type). Transition arrows demarcated by "question mark" symbols are processes triggered when a specific condition is met (e.g., the emptying of occupied pens when the average target weight is reached). The branch demarcated by a "diamond" symbol indicates the possibility of two outcomes, where 1) no metaphylaxis (default, dotted line) or 2) injectable metaphylaxis (solid line) as assigned at the feedlot level at model initialization.

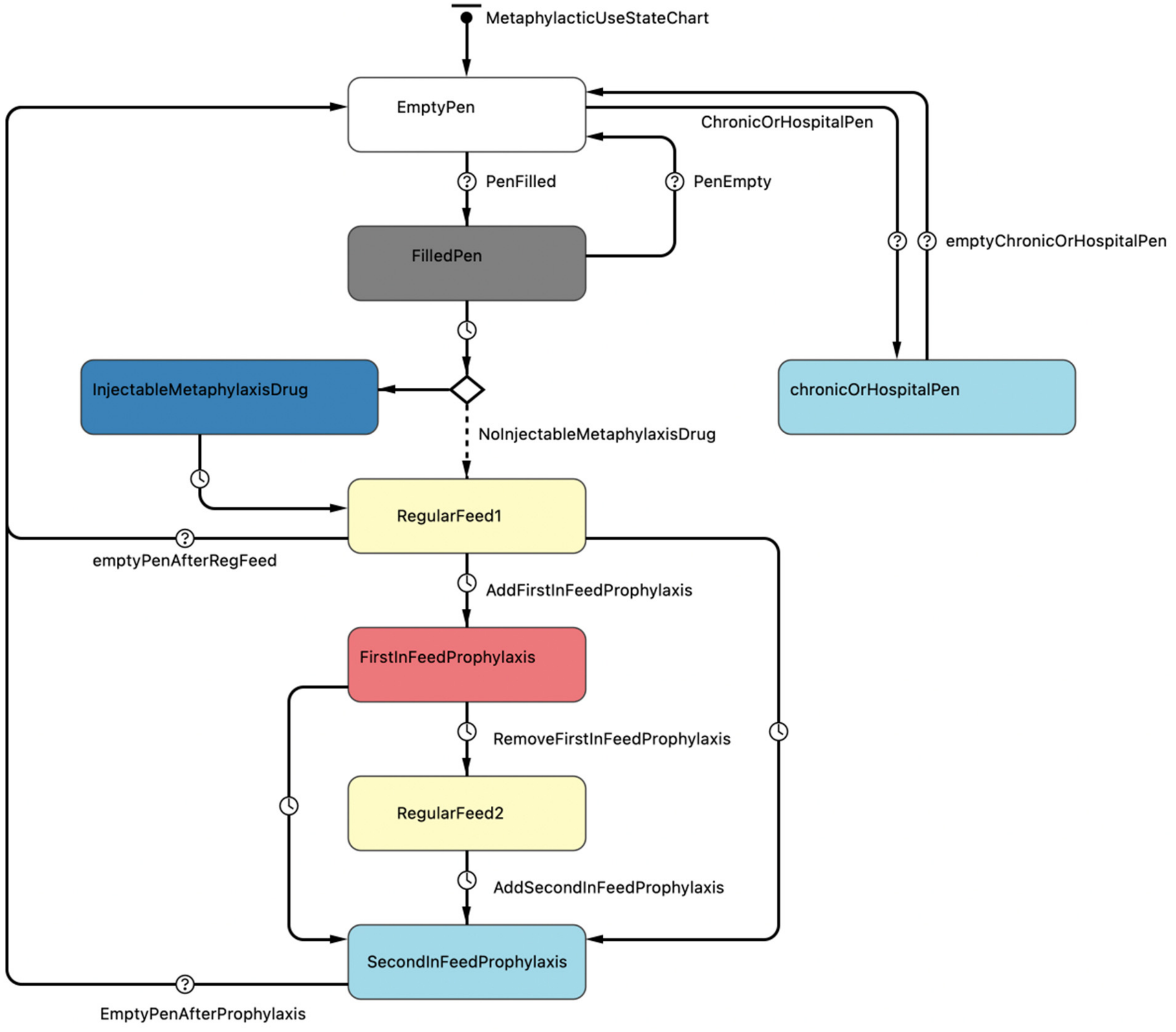

Figure 2. Representation of animal health status for each calf in the agent-based model. The "disease" states are mutually exclusive, and an animal can only receive treatment for one disease at any given time. Transition arrows demarcated by "graph" symbols are those governed by daily hazard rates derived from empirical data. Transition arrows demarcated by "envelope" symbols depend on the receipt of an "end treatment" message. The length of treatment refers to the dosing interval (for multi-day regimens) and therapeutic interval (estimated period of selective pressure following dosing interval). Branches demarcated by "diamond" symbols indicate the possibility of two outcomes, including 1) treatment success and return to healthy state (default) or 2) baseline (fixed probability for BRD, arthritis) or resistance-linked (BRD only, when enabled) treatment failure and return to disease state (termed a "relapse" for the purposes of this description).

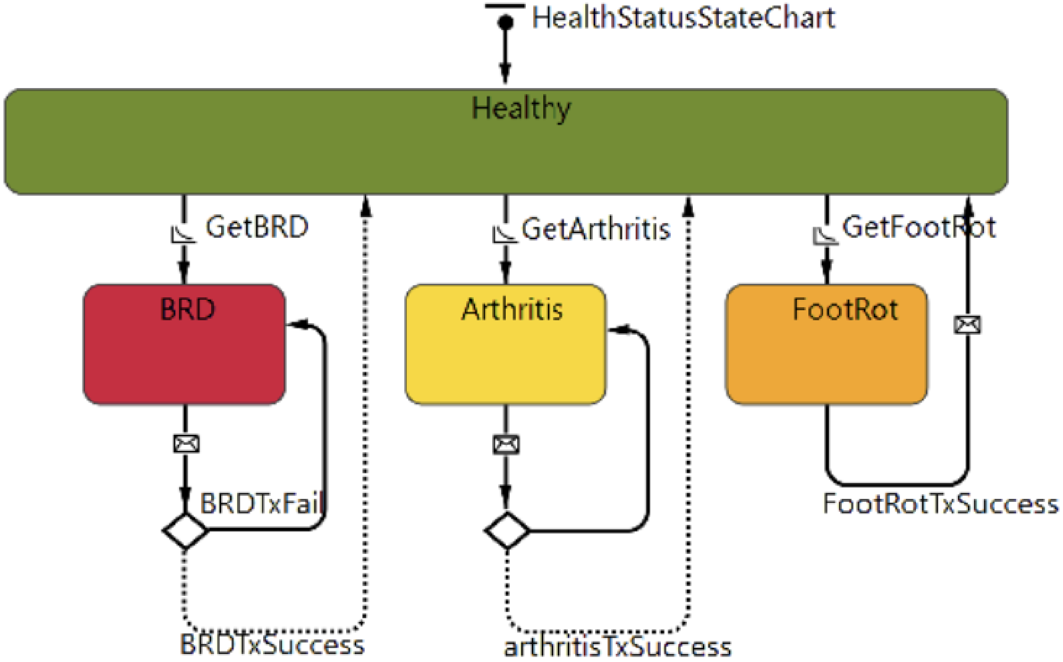

Calves become sick at a specified rate per day on feed (i.e., a daily hazard rate dependent on the number of days since feedlot entry) and are administered the prescribed antimicrobial regimen immediately upon their transition to a disease state. The daily hazard rates for the first case of each disease are drawn from empirical distributions reflecting temporal/seasonal and weight-based changes in regional disease risk shared by large private veterinary practices (see **Figure 3**). The first-case hazard rate for BRD in high-risk calves is specific to animals who receive metaphylactic tulathromycin at feedlot entry; the rate can be adjusted for other metaphylactic protocols, including "no metaphylaxis", by the risk ratios reported in a recent meta-analysis of injectable antibiotic options for BRD control [11]. Historical on-arrival AMR data are assumed to be reflected in the first-case hazard rate for BRD obtained from feedlot operations and used in the calibrations. In subsequent experiments with the model, the first-case hazard rate for BRD for an individual calf

can default to the equivalent of that for “no metaphylaxis” *if* the population of *M. haemolytica* for that calf is resistant to the antimicrobial used for metaphylaxis.

Figure 3. **Daily hazard rates for first cases of BRD, foot rot and arthritis over the feeding period**. Epidemiological curves are derived from empirical data provided by large, private veterinary practices in western Canada. The data for BRD represent approximately 590,000 high-risk, fall-placed animals at feedlot entry over 5 years (2012-2016)[1]; the data for foot rot and arthritis represent approximately 600,000 fall-placed animals over 13 years (2007-2020) provided as anonymized summaries.

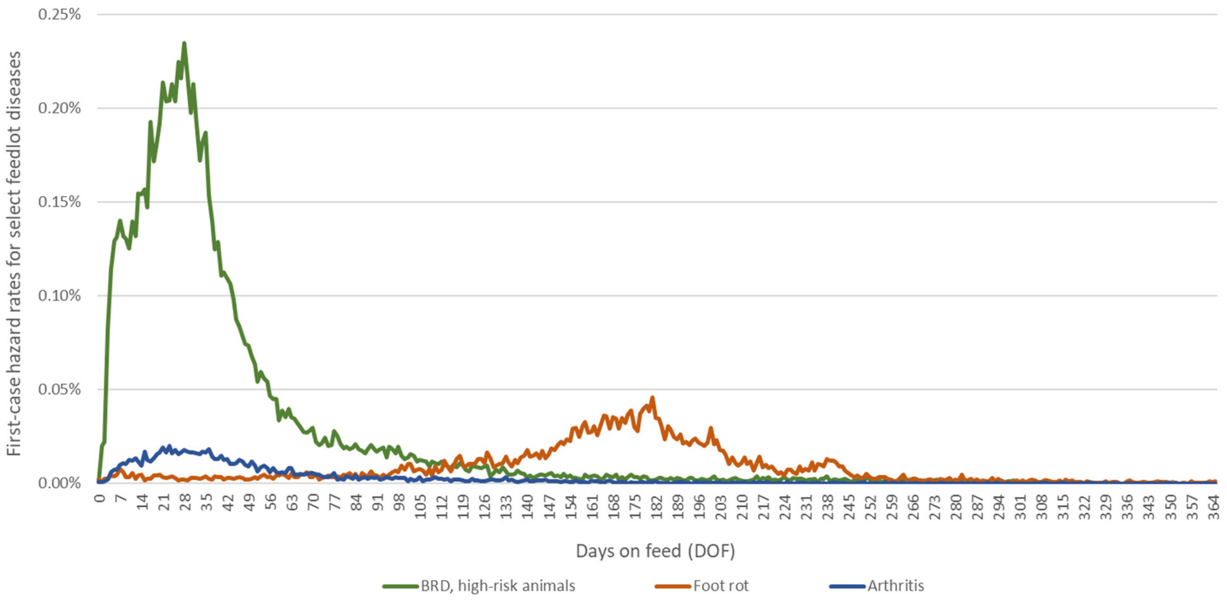

[1]The first-case hazard rate used in the calibration experiments was specific to high-risk calves that received metaphylactic tulathromycin at feedlot arrival; in future experiments, the BRD rate can be adjusted for other metaphylaxis protocols by the risk ratios calculated in O’Connor et al. [11].

BRD- and arthritis-affected calves can experience one or more relapses requiring additional individually-dosed treatments after a first case of either disease (**Figure 2**, **Table 1**). Calves who received treatment for a first case experience a first relapse with a fixed probability; second and third relapse probabilities are similarly conditional on the calf having had a first and second relapse, respectively. This is possible because the model tracks the individual treatment history of each calf. The probabilities of subsequent relapses for each disease are derived from regional data reflecting temporal/seasonal and weight-based changes in disease risk (see **Table 1**).

Calves treated for foot rot in the empirical records available for model parameterization were assumed to have uncomplicated cases of infectious pododermatitis that responded to therapy. However, some of these calves would have been retreated if they were misdiagnosed, treated too late, or if the disease appeared in another foot. The model accounts for this possibility as calves

re-enter the total population at risk of infection after receiving individually-dosed treatment (i.e., calves return to a "healthy" state by default after the therapeutic interval had elapsed for the administered antimicrobial, see **Table 2**).

Table 1. Values and sources for parameters in the baseline and calibrations versions of the agent-based feedlot model

**Parameter**	**Condition**	**Value in baseline model**	**Value selected for calibration experiments**	**Source or rationale, if applicable**
Feedlot and pen parameters				
Number of cattle per pen		**200**, user defined	200	Representation of a moderate-sized feedlot; optimization of computational effort
Number of pens across		**10**, user defined	10	
Number of pens high		**5**, user defined	3	
Cattle parameters				
Proportion of high-risk animals entering feedlot		**0-100%**, user defined	100%	
Proportion of steers entering feedlot		**0-100%**, user defined	100%	
Arrival weight for high-risk animals	*Applies to recently weaned and/or lighter-weight calves*	Value selected from uniform distribution, range **500-600 pounds**	Value selected from uniform distribution, range **500-600 pounds**	(12, 13)
Arrival weight for low-risk animals	*Applies to backgrounded and/or heavier-weight yearlings*	Value selected from uniform distribution , range **800-900 pounds**	N/A<br><br>No low-risk, heavyweight animals in calibration	(12, 13)
Average daily gain (ADG) for healthy steers	*Applies to steers with no BRD or arthritis history*	Value selected from normal distribution with **μ = 3.46** and **σ = 0.46 pounds per day**	Value selected from normal distribution with **μ = 3.46** and **σ = 0.46 pounds per day**	(13, 14)
Average daily gain (ADG) for healthy heifers	*Applies to heifers with no BRD or arthritis history*	Value selected from normal distribution with **μ = 3.00** and **σ = 0.37 pounds per day**	N/A<br><br>All animals in calibration are steers	(13, 14)
Average daily gain (ADG) for animals of either sex being treated in the hospital pen	*Applies to animals temporarily housed in the hospital pen for arthritis treatment*	Fixed value of **0.00 pounds per day**	Fixed value of **0.00 pounds per day**	Model parsimony; consultation with feedlot veterinarians
Percentage change in ADG for animals of either sex with first case of BRD	*Applies for remainder of feeding period to animals with single diagnosis (i.e., first case) of BRD*	**-0.64%**	**-0.64%**	(15)

Parameter	Condition	Value in baseline model	Value selected for calibration experiments	Source or rationale, if applicable
Percentage change in ADG for animals of either sex with first or subsequent relapse of BRD	*Applies for remainder of feeding period to animals with more than one diagnosis (i.e., one or more relapses) of BRD*	**-5.77%**	**-5.77%**	(15)
Percentage change in ADG for arthritis-affected animals of either sex before 60 DOF	*Applies for remainder of feeding period to animals with first or subsequent arthritis diagnoses before 60 DOF and following treatment in hospital pen*	**-0.69%**	**-0.69%**	(16)
Percentage change in ADG for arthritis-affected animals of either sex after 60 DOF	*Applies for remainder of feeding period to animals with first or subsequent arthritis diagnoses after 60 DOF and following treatment in hospital pen*	**-1.38%**	**-1.38%**	(16)
Target market weight for slaughter, healthy animals	*Applies to healthy animals in regular pens; pen is shipped as unit when average weight of animals reaches selected target*	Value selected from uniform distribution, range **1325-1500 pounds**	Value selected from uniform distribution, range **1325-1500 pounds**	(12, 13, 17)
High target finishing weight for slaughter, chronic pen animals		**1200 pounds**	**1200 pounds**	Consultation with feedlot veterinarians
Low target finishing weight for slaughter, chronic pen animals		**900 pounds**	**900 pounds**	Consultation with feedlot veterinarians
Disease parameters				
Probability of first BRD relapse in high-risk animals	*Conditional on having had a first case of BRD*	**21.64%**	**21.64%**<br><br>All animals in calibration are high-risk	Empirical data from approximately 55,000 high-risk calves with first cases of BRD after tulathromycin metaphylaxis (2012-2016)
Probability of second or third BRD relapse in high-risk animals	*Conditional on having had a first or second relapse of BRD, respectively*	**35.80%**	**35.80%**<br><br>All animals in calibration are high-risk	Empirical data from approximately 12,000 high-risk calves with first relapses of BRD after tulathromycin metaphylaxis (2012-2016)
Probability of first BRD relapse in low-risk animals	*Conditional on having had a first case of BRD*	**26.19%**	N/A<br><br>No low-risk animals in calibration	Empirical data from approximately 5600 low-risk calves with first cases of BRD after oxytetracycline metaphylaxis (2012-2016)

Parameter	Condition	Value in baseline model	Value selected for calibration experiments	Source or rationale, if applicable
Probability of second or third BRD relapse in low-risk animals	*Conditional on having had a first or second relapse of BRD, respectively*	**40.45%**	N/A<br><br>No low-risk animals in calibration	Empirical data from approximately 1450 low-risk calves with first relapses of BRD after oxytetracycline metaphylaxis (2012-2016)
Probability of first arthritis relapse	*Conditional on having had a first case of arthritis*	**11.58%**	**11.58%**	Empirical data from approximately 6000 fall-placed calves with first cases of arthritis (2007-2020)
Probability of second arthritis relapse	*Conditional on having had a first relapse of arthritis*	**20.46%**	**20.46%**	Empirical data from approximately 700 fall-placed calves with first relapses of arthritis (2007-2020)
Probability of third arthritis relapse	*Conditional on having had a second relapse of arthritis*	**19.58%**	**19.58%**	Empirical data from approximately 145 fall-placed calves with second relapses of arthritis (2007-2020)
Chronic pen parameters				
Percentage of animals assigned to "high target finishing weight" group at chronic pen entry		**33.3%**	**33.3%**	Model parsimony
Percentage of animals assigned to "low target finishing weight" group at chronic pen entry		**33.3%**	**33.3%**	Model parsimony
Percentage of animals assigned to "euthanasia" group at chronic pen entry		**33.3%**	**33.3%**	Model parsimony
Number of days between chronic pen weight checks		**7 days**	**7 days**	Model parsimony

To best match empirical data available for calibration, relapses are characterized as the failure of a previous therapy to adequately treat the underlying infection, prompting the continuation of symptomatic disease. Historical AMR data are assumed to be reflected in the rates of treatment failure/disease relapse obtained from feedlot operations and used in the calibrations. In subsequent experiments with the model, antimicrobial treatments delivered to BRD-affected calves will fail (i.e., animals will relapse) at a probability equivalent to the pen-level prevalence of AMR, if the

pen-level prevalence of resistance to the administered drug exceeds historical rates of retreatments (see BRD treatment failure loop, **Figure 2**). Calves with baseline or resistance-linked treatment failure remain in the "disease" state following the therapeutic interval (see **Table 2**) and receive the next antimicrobial in the treatment/relapse protocol.

Resistance

The emergent AMR status for the population of *M. haemolytica* in the nasopharynx of each calf is governed at the level of **Resistance agent**. The dynamics of resistance acquisition and loss for each antimicrobial drug are directed by separate and mutually exclusive state charts (described in detail under **Submodels**). All animals are assumed to have a population of *M. haemolytica* existing as nasopharyngeal commensals, acknowledging that this organism is not consistently culturable from all calves. Further, *M. haemolytica* is assumed to be one of the causative agents involved in the progression to clinical BRD.

Treatment

Each **Treatment agent** governs the delivery of the appropriate prophylactic, metaphylactic or therapeutic AMU protocol to an individual animal (described in detail under **Submodels**).

Table 2. Pharmacokinetic and initialization parameters for probabilistically selected antimicrobials used for prophylaxis, metaphylaxis or treatment in the feedlot model, and for antimicrobials to which acquired resistance over the feeding period is of interest

Antimicrobial class	Antimicrobial drug (example of active ingredient)	Antimicrobial drug (example of trade name)	Reason for use in feedlot model[1]	Average probability (%) of detectable resistance at arrival[2]	Withdrawal period (days)[3]	Therapeutic interval (days)[4]
**Aminoglycosides**	Spectinomycin	N/A	N/A	1.7%	N/A	N/A
**Cephalosporins**	Ceftiofur CFA	Excede 200	T	0.0%	13	9.2
	Ceftiofur HCl	Excenel RTU EZ	T	0.0%	3	1.1
**Fluoroquinolones**	Enrofloxacin	Baytril 100	T	0.4%	36	0.7
**Macrolides (15-membered ring)**	Gamithromycin	Zactran	M, T	2.4%	49	6.9
	Tulathromycin	Draxxin	M, T	2.4%	44	8.3
**Macrolides (16-membered ring)**	Tildipirosin	Zuprevo	M	4.3%	42	26.3
	Tilmicosin	Micotil	M, T	4.3%	28	3.6
	Tylosin[5]	Tylan 100	P	4.3%	0	0.1
**Potentiated sulfonamides**	Sulfadoxine	Borgal	T	2.3%	10	1.6
	Trimethoprim			0.3%	10	0.3
**Penicillins**	Ampicillin	Polyflex	N/A	1.7%	6	0.8
	Penicillin	Procaine Penicillin G	T	1.7%	5	0.1
**Phenicols**	Florfenicol	Nuflor	T	0.1%	55	3.2
	Florfenicol/ flunixin	Resflor	T	0.1%	60	3.2
**Tetracyclines**	Chlortetracycline[6]	Chlor 100 Granular Medicated Premix	P	4.9%	5-28 (dose dependent)	2.0
	Oxytetracycline	Liquamycin LA-200	M, T	4.9%	28(IM)-48(SC)	2.6

[1]Abbreviations indicating reason for use in feedlot model: P = prophylaxis, M = metaphylaxis, T = treatment, N/A = not used in model.

[2]Probability of detectable phenotypic resistance on feedlot arrival for each antimicrobial was derived from recent studies of healthy feedlot cattle in western Canada (36-42). In the calibration experiments, the probability was a weighted average of the extracted prevalence data; in the Monte Carlo experiments, the probability was randomly drawn from a modified PERT distribution considering the 95%CI as min and max values and used the average value as the most likely value (mode).

[3]The withdrawal period in days reflects those reported in the Compendium of Veterinary Products – Canada edition [41].

[4]The therapeutic interval is a crude estimate of the effective duration of selective pressure (i.e., period over which the drug is active and selection for resistance is possible). The therapeutic interval for each antimicrobial was estimated from its reported serum elimination half-life in cattle [27-36], multiplied by three.

[5]Prophylactic tylosin use did not co-select for *M. haemolytica* resistance to the other 16-membered ring macrolides in its sub-class, consistent with the observation that "the in-feed levels of tylosin [have] no effect on the prevalence of *M. haemolytica"* [38].

[6]Chlortetracycline at both the "high" and "low" dosages were fully linked to each other and to oxytetracycline. When the "low dose" of chlortetracycline was used, the calibrated "selection probability" for tetracyclines was adjusted by a multiplier (0.2) that reflects the average concentration of that regimen relative to the "high dose" regimen. The multiplier was estimated from AMU data collected by the Canadian Integrated Program for Antimicrobial Resistance Surveillance [referenced in Hannon et al. [58]] and a series of expert interviews with feedlot veterinarians.

Process Overview and Scheduling

The baseline model describes a typical, small (6,000 animals) to mid-sized (10,000 animals) western Canadian feedlot, populated by auction-sourced beef steer calves arriving in the fall. Calves with this risk profile are most often recently weaned and lighter-weight animals, and are more likely than heavier and older animals to be in the early stages of respiratory disease [3], and to receive metaphylactic antimicrobials at feedlot entry [6]. The model operates on continuous calendar time and the convenience time unit is days. The simulation is run for a period of one year. Events in the model occur at either 1) a fixed time, following the occurrence of another event (e.g., in-feed AMU exposure after a particular number of days on feed (DOF)); or 2) an arbitrary point in time, driven by a daily incidence rate or as a consequence of another event (e.g., receipt of a "transmission" message from a connected calf). In the baseline model, agents are assumed to immediately and perfectly perceive transitions in their health and AMR status that trigger treatment decisions.

The model simulates the arrival by truck of lightweight steers at random intervals starting October 1st (see **Table 1** for weight parameters [12-13]). The cattle deliveries increase in frequency from the start of the "fall run" in October and peak in November before slowing again until the feedlot reaches full capacity in December. The size of the feedlot is specified by parameters governing the number of pens and number of cattle per pen. For the calibration experiments, the feedlot consisted of 28 home pens, one chronic sick pen, and one hospital pen arranged in rows (n=30 total pens); the home pens are filled successively from left to right with 200 calves each. The necessary infrastructure and data are present to incorporate heavier-weight and/or heifer calves in pens and feedlots of different sizes in future experiments with the model. The default model setting fills individual home pens with calves of the same sex and weight (i.e., BRD risk) category. Pen-level variation in other risk factors including origin (auction or ranch-direct) and vaccination status (yes or no) could likewise be integrated as data become available.

All animals in the baseline configurations receive metaphylactic tulathromycin (i.e., Draxxin) by default. However, the choice of metaphylactic antimicrobial (if any) can be probabilistically selected at model initialization if desired (see **Figure S1**). In subsequent experiments with the model, calves with on-arrival resistance to the antimicrobial drug used for metaphylaxis will be subject to a first-case hazard rate for BRD equivalent to those who receive "no metaphylaxis". This adjustment changes the size but not timing of the peaks in **Figure 3**. The probability that the

population of *M. haemolytica* in each calf has detectable resistance on arrival is derived from empirical data in the published literature [9-10, 18-22] (see **Table 2**).

Prophylactic antimicrobials to prevent histophilosis and liver abscesses are delivered at the pen level to all calves in the feedlot via medicated feed. The prophylactic regimen (drug choice, start date, duration, and dosage with or without pulse delivery regime) for each indication is probabilistically selected at model initialization (see **Figure S2**). The selected protocol applies to all home pens in the feedlot for a unique model run (i.e., one replication in a calibration experiment), but can be set to vary across multiple iterations within experiments. The variation described in the literature is expected to best reflect the AMU conditions that generated the AMR data used in calibration experiments.

Calves are assumed to be healthy at feedlot arrival, and are assigned at entry a rate of average daily gain (ADG) drawn from a normal distribution (see **Table 1** for weight gain parameters [13-16]). Calves become sick at a daily hazard rate dependent on the number of days since feedlot entry (**Figure 3**), and are administered the prescribed antimicrobial regimen immediately upon their transition to a disease state. The therapeutic regimen for each indication is probabilistically selected at model initialization (see **Figures S3-S5**) and varies by animal weight at infection (all diseases) and risk at feedlot entry (BRD only). Calves that transition to a "disease" state due to BRD or arthritis are assigned a reduced rate of ADG (i.e., gain weight more slowly) for the duration of their time in the feedlot (see **Table 1)**; the reductions in ADG are additive for calves affected by both diseases over the feeding period. Long-term ADG was not substantially affected in calves with foot rot in a previous report [23], and thus the rate of growth does not change for these animals.

Therapy is assumed to be effective for uncomplicated foot rot, and calves return to a "healthy" state by default after the therapeutic interval has elapsed for the administered antimicrobial (see **Table 2**). If 10% or more of the calves in a shared pen are diagnosed with foot rot in a single feeding period, a course of in-feed antimicrobials is administered at the pen level as part of a preventative "outbreak protocol" (see **Figure S5**).

BRD- and arthritis-affected calves can experience one or more relapses requiring additional individually-dosed treatments after a first case of either disease. Calves who received treatment

for a first case experience a first relapse with a fixed probability; second and third relapse probabilities are similarly conditional on the calf having experienced a first and second relapse, respectively. The probabilities of subsequent relapses for each disease are derived from regional data reflecting temporal/seasonal and weight-based changes in disease risk (**see Table 1**).

All animals in the same pen are connected to each other via a transmission network or "spatial neighbourhood" configured by the user at model initialization. Networks can be reconfigured by the user in future experiments to include distance-based connections and/or connections between animals in adjacent pens. Calves being treated for first or subsequent cases of arthritis are moved to a specialty pen for acutely sick animals requiring multi-day therapeutic regimens, designated the "hospital pen" (see **Figures S4, S6**); the calf's connections are reconfigured to include their temporary pen-mates during their hospital stay. These animals return to their home pens after their final doses are administered, and are the only potential vectors for the inter-pen spread of resistance in the baseline model where both cattle re-sorting and inter-pen connections are disabled. Calves do not gain weight while they are housed in the hospital pen and are temporarily assigned an ADG equal to zero; once they return to their home pens, the animals resume weight gain at the reduced rate described in **Table 1**. If the home pen has been depopulated (i.e., sent for slaughter) before the calf returns, the animal is instead transferred to the "chronic pen" as there is no "rail pen" specified in the model.

Chronically sick animals, including a proportion of heavyweight calves with arthritis and/or calves experiencing a third relapse from either BRD or arthritis, are permanently moved to the second specialty pen designated the "chronic pen" (**Figures S3-S4, S6**). Calves housed in this pen do not receive prophylactic (i.e., in-feed) or therapeutic antimicrobials to prevent or treat illness, given that they have not and are not expected to respond to established treatment protocols; these animals continue to gain weight at the reduced rate of ADG in accordance with their diagnosis. As in the hospital pen, the connections of calves in the chronic pen are reconfigured to include their new pen-mates.

At chronic pen entry, calves are probabilistically assigned to one of three ultimate destinations consistent with what might occur for large feedlot operations. One-third of the calves who enter the chronic pen are euthanized, and the remainder are evenly split between 1) those who are

eventually shipped to a slaughter plant (as with healthy calves), as they achieve near-to-target weights in a reasonable time frame; and 2) those who are slaughtered at a reduced final weight (see **Table 1** for chronic pen parameters). The disposition and/or weight of chronically sick animals was evaluated every seven days. The animals are sent for slaughter when they reach the minimum finishing weights specified in **Table 1**. Animals in the chronic pen which fail to reach their minimum target weight before the end of the feeding period are euthanized.

The market or target animal weight for a unique model run is randomly selected at initialization (see **Table 1** for target weight parameters [17]). When the average weight of the animals in a particular pen reaches the selected target, the pen is emptied and the calves are shipped for slaughter and processing. Calves in any pen type can die of disease prior to reaching their target weight, and this is captured in the model by a daily mortality rate dependent on the number of days since feedlot entry (see **Figure S6**). Daily mortality rates subdivided by cause are drawn from empirical distributions reflecting temporal/seasonal environmental changes in risk (see **Figures 4 and 5**). For the purposes of the model, it is assumed that death due to BRD is conditional on having experienced at least a first case of BRD requiring treatment with antimicrobials. While it is possible for animals to die from BRD without being diagnosed and treated, empirical data attributing death loss to treated *versus* untreated animals was not available. This assumption does not impact total death losses in the model.

Figure 4. Daily mortality rates due to BRD over the feeding period. Epidemiological curve is derived from empirical data provided by a large, private veterinary practice in western Canada. These data represent approximately 700,000 high-risk, fall-placed animals at feedlot entry over 5 years (2012-2016).

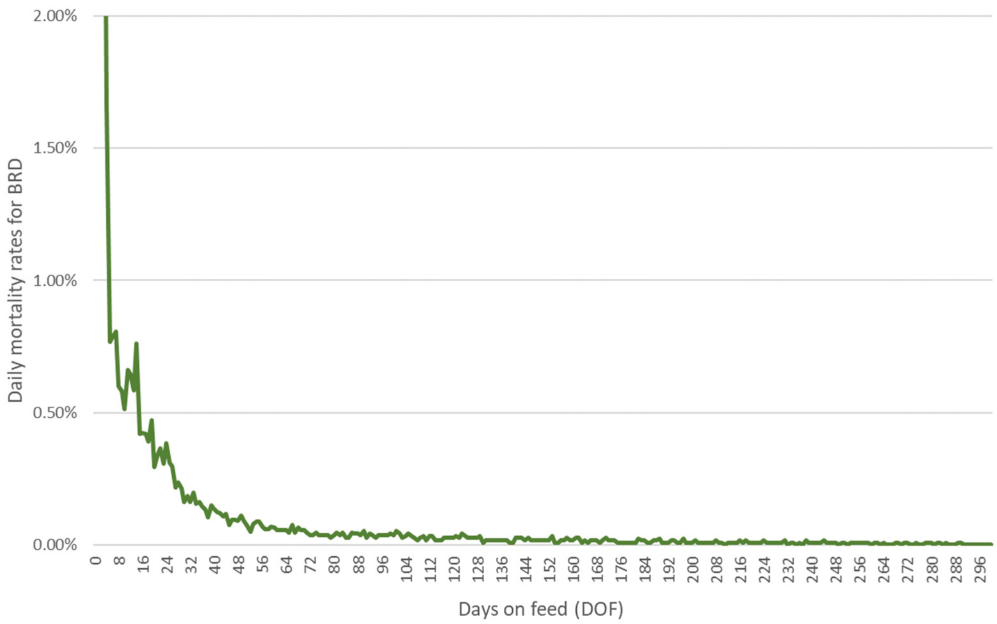

Figure 5. Daily mortality rates due to histophilosis and other causes over the feeding period. Epidemiological curves are derived from empirical data provided by a large, private veterinary practice in western Canada. These data represent approximately 700,000 high-risk, fall-placed animals at feedlot entry over 5 years (2012-2016).

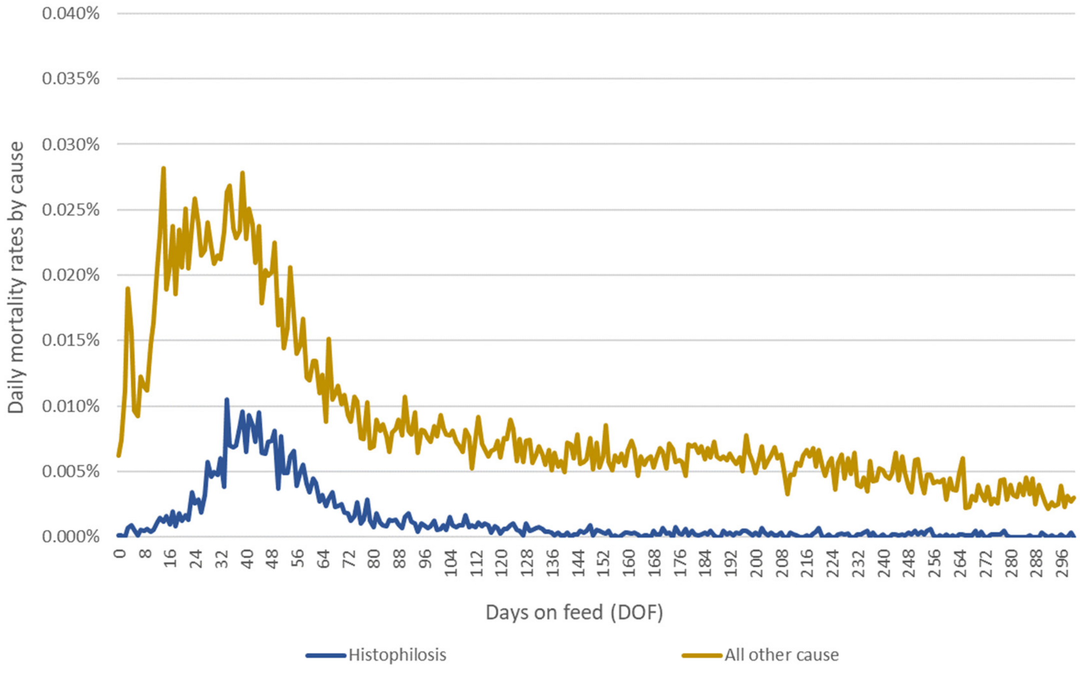

Design Concepts

Basic Principles

BRD is the most common and costly disease affecting North American beef cattle [24], and is therefore a major determinant of AMU in feedlots [6-8]. AMU exerts a selective pressure on populations of microorganisms, favoring the survival of bacteria that harbor resistance genes. In the absence of selective pressure from AMU, the related fitness costs have the potential to make resistance expression less advantageous. AMR can also disseminate directly between animals via the transfer of resistance genes and/or microorganisms with resistance genes. Antimicrobial drugs from the same class (e.g., tetracyclines) or sub-class (e.g., 15-membered ring macrolides) are assumed to be equally vulnerable to the genetic mechanism(s) conferring resistance to populations of bacteria. The model thus simulates the co-selection, co-waning and co-transmission of detectable resistance for drugs in the same class.

The relative contributions of the major drivers of AMR spread at the population level are unknown [25]; specifically, there are insufficient data to determine quantitatively how important selection pressure from AMU is for the emergence of AMR relative to contagious spread [26]. Hypotheses about how AMR in BRD-associated pathogen *M. haemolytica* emerges and spreads in the feedlot environment are consequently represented by distinct model configurations.

Emergence

The primary emergent outcomes of the model are the time-varying prevalences of detectable AMR in *M. haemolytica* to select classes of antimicrobial drugs, arising from the combination of initial conditions, temporal trends in feedlot disease, and prophylactic and therapeutic treatment selections. Related outputs emerging from the model *for each antimicrobial class* and at *each time point* included 1) the cumulative number of uses of antimicrobials belonging to that class; 2) the cumulative number of acquired resistance events due to selection and 3) the cumulative number of acquired resistance events due to transmission.

AMR acquisition and loss events are simulated stochastically in response to emergent patterns of prophylactic and therapeutic AMU. Disease occurs stochastically in the model, and therefore the number and timing of antimicrobial uses for therapy is also emergent. The transmission of AMR between calves occurs at a time-dependent stochastic rate specific to each antimicrobial class, and

its emergence depended in part on the prevalence of animals with detectable resistance available to transmit to susceptible contacts.

Resistance-linked treatment failure could not be decoupled from other-cause treatment failure in the empirical data available for parameterization, and was therefore externally specified in the calibration version of the model. In the operational version of the model, responsiveness to detectable resistance allows for increasingly complex feedback between AMU and AMR. The first-case hazard rate for BRD defaults to the equivalent of that for "no metaphylaxis" if the local population of *M. haemolytica* for a calf is resistant in that calf to the antimicrobial used for metaphylaxis. The resultant higher first-case hazard rate for BRD increases the potential for AMU-associated selection pressure.

Similarly, if the prevalence of pen-level AMR for the antimicrobial used to treat a first or second BRD relapse exceeds the baseline failure rate, then treatment failure occurs at the higher rate equivalent to the pen-level prevalence of AMR. While not all treatment failures will be due to AMR, the exact probability is unknown and as such this assumption that AMR above the baseline will result in additional treatment failure is a worst-case scenario assumption. The decreasing success of treatment can result in additional BRD cases, emergent patterns of AMU that apply selective pressure, and the associated additional potential for emergent AMR.

The number and timing of animals moving to the chronic pen are other emergent outcomes. A proportion of heavyweight calves with arthritis and/or calves experiencing a third relapse from either BRD or arthritis are deemed "chronically ill" and moved to this specialty pen.

Adaptation

No specific adaptive behaviors on the part of agents are applied in the baseline version of the model.

Objectives

No goal-seeking on the part of agents is applied in the model.

Learning

No learning on the part of agents is applied in the model.

Prediction

The prediction is that the time-varying prevalence of population-level AMR to select classes of antimicrobials will be an emergent pattern of the model, given the assumptions about on-arrival

AMR prevalence, disease incidence and timing, and AMU exposures. The extent to which AMU-linked selective acquisition of resistance *versus* contagious acquisition of resistance is responsible for emergence is likewise predicted to impact the primary emergent outcome.

Sensing

In the baseline version of the model, agents are assumed to immediately and perfectly perceive transitions in their health and AMR states that trigger treatment decisions.

Interaction

Cattle in the same pen interact with each other and can transmit AMR to the populations of *M. haemolytica* local to each animal. Transmission messages are sent from animals with detectable AMR to a randomly selected pen-mate at the calibrated contact rate. The per-day contact rate is adjusted by the calibrated stress multiplier to reflect the increased likelihood of transmission when animals are physiologically stressed. When a transmission message is received by a susceptible calf, the contact is considered to be "effective" (i.e., results in contagious transmission). Dependent on their health status, animals might be moved to the hospital or chronic sick pens, thereby modifying their contact networks.

The use of antimicrobials applies selection pressure to the *M. haemolytica* population within an animal. Resistance acquired selectively or contagiously does not wane in the model if the animal is being actively exposed (i.e., within the therapeutic interval of) to the relevant drug class. In the absence of selection pressure to the *M. haemolytica* population within an animal, the potential exists for resistance to wane at a specified rate. Immediately after waning, an animal is once again at risk of acquiring resistance if subjected to selective pressure due to AMU or if exposed via contact with pen-mates (i.e., a calf cannot become "immune" to resistance).

Stochasticity

Cattle arrive at the feedlot as part of a stochastic process, with a variable number of animals arriving and filling each pen within two days at varying intervals between October to December. The random assignment of 1) arrival weight (from a uniform distribution); 2) rate of ADG (from a normal distribution); and 3) probability of detectable resistance to each antimicrobial class (from a PERT distribution) to incoming lightweight steers at model initialization. The target market weight for healthy animals was also randomly selected from a uniform distribution at model startup. The prophylactic and therapeutic AMU protocols for a single realization of the model were randomized across a range of plausible alternatives at the start of the simulation.

The calves in the model become sick with first occurrences of select diseases at stochastic rates drawn from exponential distributions informed by the empirical data summarized in **Figure 3**. Treatment failures and associated relapses of BRD and arthritis were likewise random events that occurred with fixed probabilities. When AMR responsive mechanisms are initialized in the model, BRD treatment failures in excess of the baseline probability are responsive to emergent pen-level AMR and are therefore stochastic processes (**Table 1**). AMR acquisition and loss events were simulated stochastically per the calibrated selection and waning rates, respectively, and depended in part on emergent patterns of prophylactic and therapeutic AMU. Contagious transmission events were stochastic processes arising from both 1) the calibrated contact rates and 2) random contacts between calves in the same pen with discordant resistance status.

Collectives

The model's architecture reflects the hierarchical structure of a typical western Canadian feedlot. Agents that represent the phenotypic resistance status of respiratory bacteria to select antimicrobial classes are collected within cattle; cattle are collected into pens, which are collected into a feedlot. Animal-level delivery of *therapeutic* antimicrobials impact the resistance phenotypes nested within individual calves. Pen-level delivery of *prophylactic* and *metaphylactic* antimicrobials apply to all calves within that pen and impact the resistance phenotypes nested within those calves. Feedlot management decisions, including decisions about AMU regimens, calf allocations and re-sorting, apply to all pens in the feedlot for a single model run.

Observation

In single-run configuration, the model opens a graphical representation of the feedlot with colours and patterns that indicate the state of the pen- and calf-level agents. These depictions enable the user to visually confirm that the code results in the intended agent behaviors during the simulation. The model likewise displays dynamic graphs for single runs that update each model day and permit the real-time observation of resistance prevalence for each antimicrobial class, disease and mortality incidence, resistance acquisition incidence by route (i.e., selection or transmission), and chronic sick and hospital pen usage.

Further, detailed model results are output to a Microsoft Excel workbook at the end of the model run. These results include a complete readout of parameter settings for the unique simulation. Feedlot-level summaries of disease incidence and resistance prevalence by antimicrobial class are generated each model day, and pen-level summaries of the same are generated every model month.

A summary of each pen at its market date is recorded and includes case counts for each disease and resistance prevalence by antimicrobial drug. Finally, every chronically sick calf and its ultimate destination (high finishing weight, low finishing weight, or euthanasia) is listed.

For multi-run configurations, including calibration experiments, graphics are not displayed and the detailed file output is suppressed. Instead, a multi-run summary table with a reduced set of outputs is generated in Excel for both the pen and feedlot levels. Depending on the unit of analysis, each row in the summary table represents a single pen or feedlot from one model realization. Summaries of animal growth (e.g., mean arrival and finishing weights), AMU (e.g., counts of regimen applications, total amounts of drugs used), disease and mortality (e.g., counts of first and subsequent instances of disease), and specialty pen use (e.g., calf-days in hospital pen, chronic pen destination) are listed in this file.

Details

Initialization

The model is initialised as an empty feedlot at the beginning of a one-year cycle. Each production cycle starts on the 1st of October. A single one-year cycle is used for all scenarios examining AMR in *M. haemolytica.* The feedlot size and cattle arrival parameters are described in **Table 1**, and the simulation initialization settings are reported in **Table 3.**

Input Data and Parameters

Model inputs relevant to each of the feedlot, pens, cattle, and disease are displayed in **Table 1.** Parameter values are informed in part by the peer-reviewed literature, empirical data from private feedlot operations, market analyses, and expert opinion via consultations with feedlot veterinarians. In the absence of a relevant source, inputs deriving from simplifying assumptions are favoured for the calibration experiments (identified as “model parsimony” in **Table 1**). The inputs in **Table 1** are organized by subheading to better highlight the agent or state chart where the value is used; the condition(s) precipitating the use of particular values are likewise detailed in the table.

The antimicrobial drugs listed in **Table 2** include those available for use in the model and those to which acquired resistance might be of particular interest. Where relevant, the table notes if the drug is used for prophylaxis, metaphylaxis or therapy in the model, applications which are fully

detailed in **Figures S1-S5**. The AMU options are common in western Canadian feedlot medicine and were developed in consultation with feedlot experts that included the teams which provided the model's morbidity data. A "variation" option in the **Main agent** allows the user to select the default treatment option, or alternatively directs the model to probabilistically select a regimen from a defined list of commonly used protocols. For example, one of two antimicrobial drugs (ceftiofur and enrofloxacin) is randomly selected as the treatment option for first relapses of BRD in high risk calves under 1200 lbs (see **Figure S3**).

Peer-reviewed or other reliable data are not available to estimate the duration of selective pressure following treatment with an antimicrobial (i.e., the "therapeutic interval" in **Table 2**). Elimination half-lives were therefore used to estimate the time required for the drug's activity to be limited by its decreasing concentration [27-36]. Three half-lives (i.e., the time when 88% of the drug is expected to be eliminated from the animal) was used as a crude estimate of the therapeutic interval. For example, the interval used for tulathromycin in the baseline model (8.3 days), corresponds to the midpoint of the plausible values proposed as that drug's duration of effect (DOE) by Brault et al. [37]. These values can be modified on an accessible spreadsheet external to the model to account for alternative hypotheses or updated data as they become available.

Incidence timeseries for first treatment for BRD, arthritis and foot rot (**Figure 3**), as well as for mortality due to BRD (**Figure 4**), histophilosis and all other causes (**Figure 5**), are also loaded from an external file. These are represented as a daily time series for a single 1-year feeding cycle.

Submodels

Resistance

The emergent AMR status for the population of *M. haemolytica* in the nasopharynx of each calf is governed at the level of **resistance agent**; each resistance agent is associated with one of the 17 antimicrobials represented in the model (**Figure 6**). Antimicrobial drugs from the same class (e.g., tetracyclines) or sub-class (e.g., 15-membered ring macrolides) are assumed to be equally vulnerable to the relevant resistance mechanism. The model thus simulates the co-selection, co-waning and co-transmission of detectable resistance for drugs in the same class, with the following exception. In-feed tylosin use (see **Figure S2**) is assumed *not* to co-select for *M. haemolytica* resistance to injectable 16-membered ring macrolides in its sub-class, consistent with the finding in Zaheer et al. [38] that subtherapeutic tylosin had no effect on the prevalence of resistant *M.*

haemolytica. The probability that the population of *M. haemolytica* in each calf has detectable resistance to each antimicrobial class at feedlot arrival is derived from empirical data in the published literature [9-10, 18-22].

Figure 6. Representation of AMR status for the population of *M. haemolytica* in the nasopharynx of each calf in the agent-based model. The first branch demarcated by a "diamond" symbol indicates the possibility of two outcomes, where 1) resistance is present on arrival (default, dotted line, to a composite "resistant" state with sub-states) or 2) resistance is not present on arrival (solid line, to a "non-resistant" state) as derived from empirical data. Two of the transition arrows demarcated by "envelope" symbols depend on the receipt of a "selection" or "transmission" message. Other transition arrows demarcated by "envelope" symbols reflect "co-selection", "co-transmission" or "co-waning" messages from linked antimicrobial drugs belonging to the same class or sub-class and undergoing the same transition (see **Figure S8**). Transition arrows with envelopes within the resistant agent reflect messages related to the status of antimicrobial exposure impacting whether resistance within the bacterial population has the potential to wane.

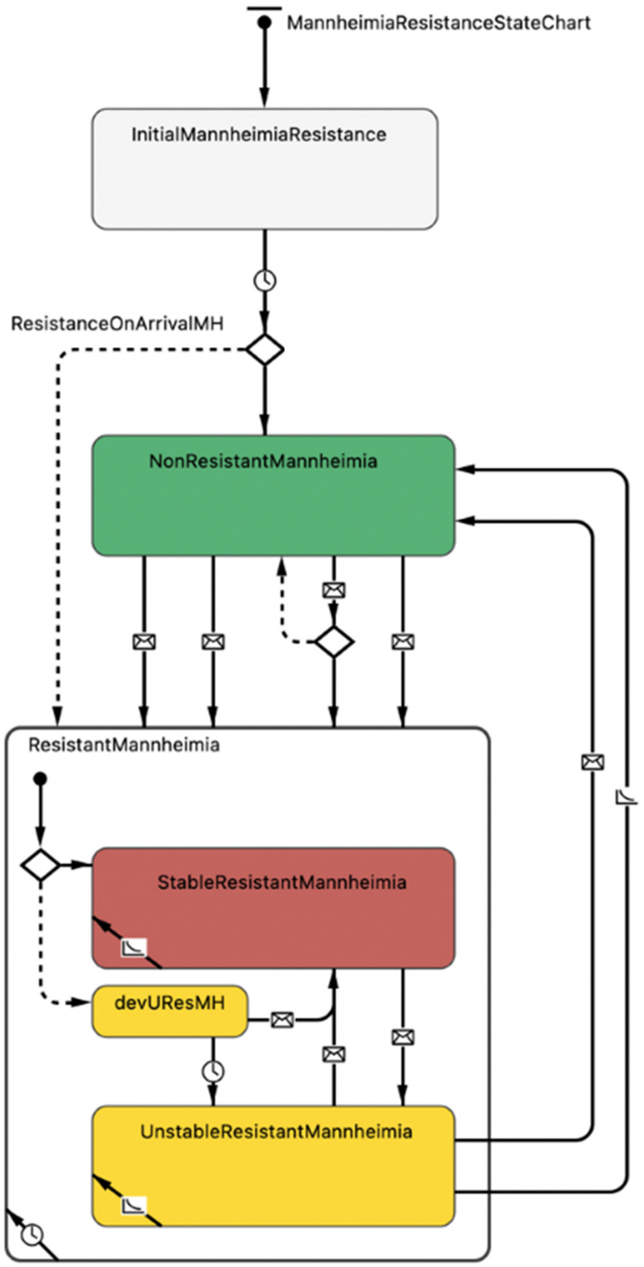

M. haemolytica populations within calves acquire detectable resistance at the calibrated "selection probability", the per-day likelihood of developing a resistant phenotype in response to antimicrobial drug exposure (**Figure 7**). The selection probability is active during therapy (i.e., the dosing period) and for the period of selective pressure following the final -- and often only -- dose (i.e., the therapeutic interval, see **Table 2**). During active exposure to the relevant drug, resistant *M. haemolytica* is considered "stable" and cannot wane. After the therapeutic interval has elapsed and in the absence of selective pressure for a particular antimicrobial, the population of *M. haemolytica* can lose its detectable resistance at the calibrated "waning rate" (i.e., is "unstably" resistant). Resistance present on arrival is assumed to be temporarily stable in a 48-hour "delay" state, such that it cannot transmit or wane *even in the absence of* selective AMU pressure. This lag is included to establish expected probabilities of population-level resistance during the period the pen is filling.

In addition to selection, a local population of *M. haemolytica* can acquire detectable resistance at the calibrated "contact rate" by way of contagious spread from a connected calf in a shared pen (see **Figure 7**). The calibrated contact rate parameter is the rate per day that an animal with detectable resistance sends a "transmission" message to a randomly selected pen-mate. When the message is received by a calf with a susceptible *M. haemolytica* population, it is assumed to be an effective (i.e., transmitting) contact. This simplifying strategy was chosen to minimize the number of messages associated with transmission and the associated computational burden. As with on-arrival resistance, detectable resistance acquired contagiously (i.e., via transmission) is subject to a 48-hour lag before it can 1) wane at the calibrated rate, given the absence of selective pressure for the relevant drug; or 2) transmit at the calibrated contact rate to unaffected pen-mates. This lag is included to permit the resistant population to establish and facilitate the estimation of calibrated parameters. Similar lags between initial colonization, bacterial reproduction, and successful transmission would be expected in a true biological infection.

Figure 7. Processes underlying the acquisition and waning of phenotypic resistance for the sentinel nasopharyngeal organisms unique to each calf. Transitions demarcated by "speech bubble" symbols are triggered by the receipt of a message from another agent in the model (i.e., the calf's home pen or a connected calf in the same pen).

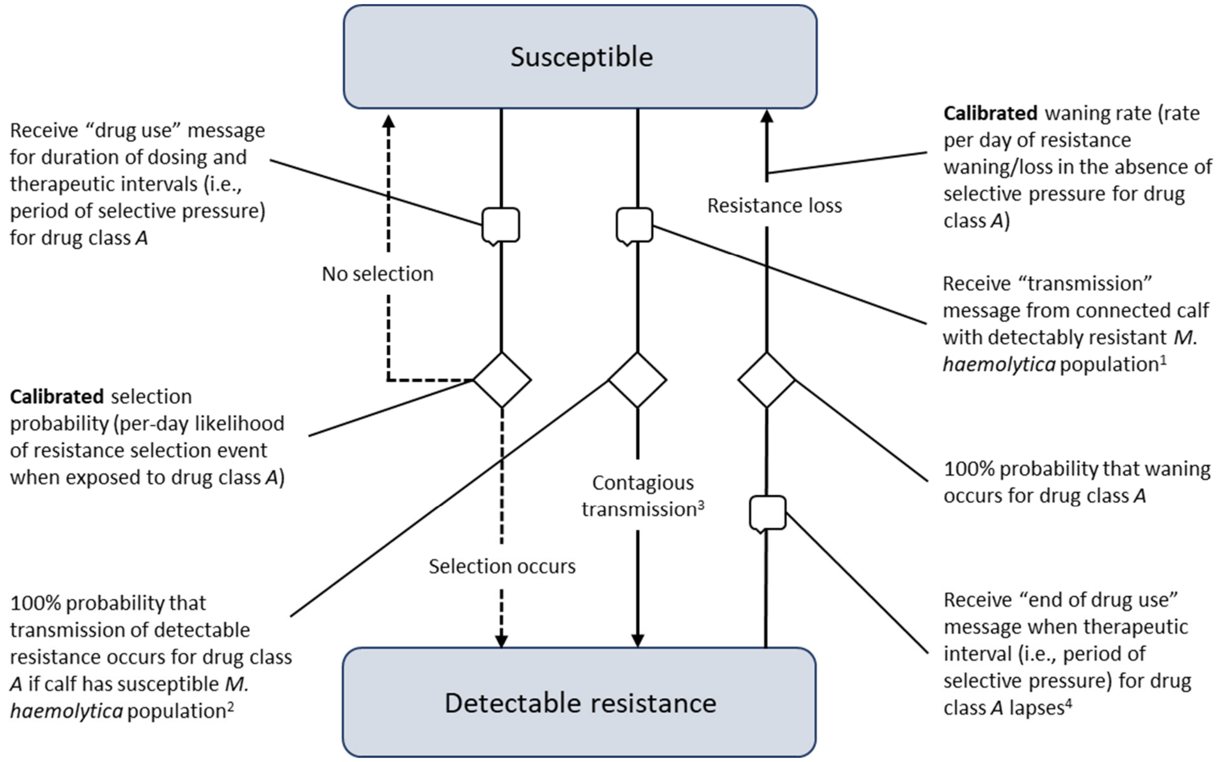

[1]Transmission messages are sent from animals with detectable resistance to a randomly-selected pen-mate at the calibrated contact rate, and the baseline contact rate is adjusted by the calibrated stress multiplier. There is a 48-hour delay before on-arrival resistance can be transmitted to pen-mates to establish the appropriate population-level resistance at simulation start.
[2]When a transmission message is received by a calf with a susceptible *M. haemolytica* population, the contact is always "effective" (i.e., results in contagious transmission).
[3]There is a 48-hour delay before contagiously-acquired resistance can wane and/or be transmitted to pen-mates, to permit the resistant population to become established in the affected animal.
[4]Resistance acquired selectively or contagiously cannot wane if the animal is being actively exposed to (i.e., within the therapeutic interval of) the drug class of relevance; further, there is a 48-hour delay before on-arrival resistance can wane to establish the appropriate population-level resistance at simulation start.

The potential for transmission over the feeding period is mediated by a calibrated "stress multiplier", a dynamic parameter responsive to the temporal distribution of BRD events at the population level. The per-day contact rate is adjusted to reflect the increased likelihood of contagious transmission when animals are physiologically stressed and shedding higher numbers of respiratory pathogens [39]. The multiplier's effect is governed by a step function derived from cumulative incidence data (see **Figure 8**) and the cluster analysis in Babcock et al. [40]. The combined impact of the calibrated stress multiplier and step function can vary from zero (i.e., no incremental effect on the per-day contact rate) to some positive increment corresponding to the

product of the stress multiplier and DOF-determined step function value, added to the per-day contact rate.

Figure 8. Cumulative incidence of first-case BRD diagnoses in high-risk calves through 120 DOF. The epidemiological curve (blue) is derived from empirical data provided by a large, private veterinary practice in western Canada. These data represent approximately 590,000 fall-placed animals over 5 years (2012-2016). A step function (black) is superimposed over the cumulative incidence curve; the red vertical lines delimit where unique regions of the curve (i.e., distinct periods of physiological stress/pathogen shedding) correspond to DOF[1]. The stress effect multiplier adjusts the baseline contact rate to account for changing transmission potential over the feeding period as determined by the step function (e.g., after 70 DOF when the step function reduces to zero, the multiplier has no impact on the baseline contact rate)[2].

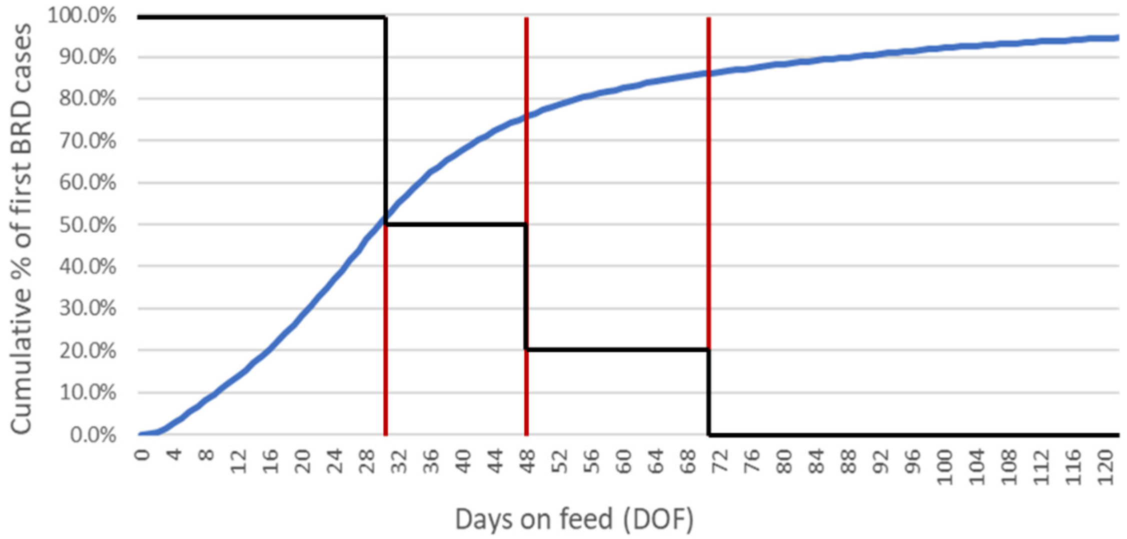

[1]By 30 DOF, 50% of the high-risk animals who will get sick have a first case of BRD; by 48 DOF, 75% will have a first case of BRD; by 70 DOF, 85% will have a first case of BRD.
[2]The impact of the calibrated stress multiplier on the contact rate in the **baseline** scenario is reduced by half (from 10 to 5) between 30 and 48 DOF, reduced to 2 between 48 and 70 DOF, and reduced to 0 after 70 DOF.

Treatment

Each **treatment agent** governs the delivery of the appropriate AMU protocol to an individual animal (**Figure S7**). For all metaphylactic and most therapeutic indications, the antimicrobial is administered in a single injectable dose. For all prophylactic (i.e., in-feed) indications and treatments for arthritis, the antimicrobial is administered in a multi-day regimen (**Figures S2, S4**). During therapy (i.e., the "dosing period") and the subsequent therapeutic interval (see **Table 2**), the selection probability is active and can lead to detectable resistance in the population of *M. haemolytica* for the treated animal. After the therapeutic interval has elapsed following a final dose in a regimen, an "end treatment" message is sent to the resistance agent for the treated calf. The message triggers the transition from "stable" to "unstable" resistance (see **Resistance agent**), such

that detectable resistance is eligible to wane. Some AMU regimens (e.g., the pulse application of chlortetracycline in feed) are represented by multiple treatment agents. Treatment agents are deleted after the withdrawal period [41] for the associated antimicrobial has expired for that animal (**Table 2**).

Model Calibration

Calibration refers to the systematic estimation of static input values that minimize the dissimilarity between the model's emergent behaviour (i.e., the time-varying prevalence of AMR across the feeding period) and the observed or empirical data that serve as calibration "targets" [42]. Automated calibration experiments using the widely implemented OptQuest global optimization routine were created in the commercial software program [43-45]. Key parameters linked to the emergence and inter-animal spread of resistance on feedlots (i.e., resistance selection probabilities, resistance waning rates, baseline contact rates, and stress multipliers) were unknown values that were expected to differ by drug class (see **Figure 7**). The unknown parameters were estimated for distinct configurations of the baseline model by calibrating the model to previously reported resistance data for each antimicrobial class.

The automated procedure used in this instance offers substantial advantages over manual calibration exercises in its optimization algorithms and capacity to manage stochastics and uncertainty in other model inputs. While the authors of this model [46-50] and others [51-54] have contributed sophisticated computational statistics and machine learning techniques that excel in supporting automated parameter estimation via sampling in higher dimensional parameter spaces, the current results are well supported by the optimization-based approach used here.

Reference Data Set

A rapid search of the literature was performed to identify relevant sources of resistance prevalence data for *M. haemolytica* isolates from healthy feedlot calves at various points across the feeding period. Studies concerning samples from primarily sick or dead cattle were excluded, given that these animals were more likely to have been treated with multiple classes or courses of antimicrobials and were not representative of the general feedlot population. Raw data from pertinent studies of western Canadian feedlot cattle [9-10, 18-22] were extracted to a spreadsheet

in MS Excel, and included the average DOF at time of sample, the total number of tested isolates, and the percentage of phenotypically resistant isolates for each antimicrobial of interest.

Data points clustered closely in time were grouped together into DOF ranges that best defined the unique phases of the feeding period. Prevalence data from isolates collected at feedlot arrival were classified as occurring at 1 DOF; the DOF for subsequent time points were selected to 1) coincide with a historical reference dataset [18] and DOF relevant to management events in the feeding period; *or* 2) reflect the midpoint of the DOF range in the reported data. The authors in [18] supplied additional data and details to permit the calculation of resistance prevalence for specific DOF (P. Morley, personal communication, 2018).

A custom longitudinal dataset with updated phenotypic resistance prevalence values was synthesized from the extracted data from all sources for *M. haemolytica* (see **Figure 9**). Because antimicrobial drugs from the same class or sub-class were assumed to be equally vulnerable to the relevant resistance mechanism (see the drug linkage matrix in **Figure S8**)), the drug with the most complete data set (i.e., one or more data points for each time point) was selected to represent all others in its class. Prevalence and exact 95% confidence intervals were estimated using an intercept-only (or null) generalized estimating equations model in SAS version 9.4 with a binary outcome, binomial distribution and logit link function for each antimicrobial class of interest at each time point (1, 13, 50, 70, 105 and 170 DOF), accounting for clustering by study with a repeated term and exchangeable covariance structure. If the model did not converge, exact confidence intervals were determined using the Clopper-Pearson estimation method. The percentage of resistant isolates at each time point was therefore an average of the raw data values available for that range, weighted by the total number of isolates tested. A prevalence estimate was unavailable for the tetracycline class at 13 DOF; in-feed chlortetracycline was not provided to the animals in the only study with data from that time point [9], and the level of detectable tetracycline resistance (<1%) was an outlier among comparable studies.

Figure 9. Percentage of *Mannheimia haemolytica* isolates with detectable phenotypic resistance to select antimicrobial classes[1] over the feeding period. The custom longitudinal dataset was synthesized from recent empirical studies of antimicrobial resistance prevalence in western Canadian feedlot cattle from mixed origins[2] and used as external targets against which to calibrate the model.

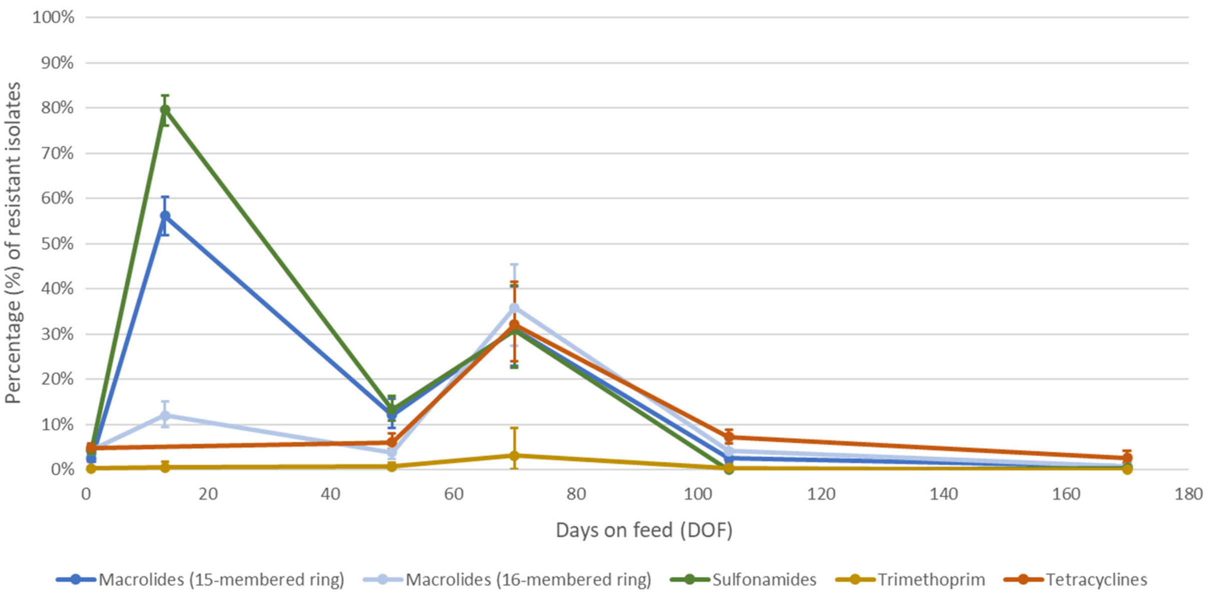

[1]Antimicrobial classes were selected for calibration if the prevalence of detectable resistance was >1% at any time point **and** the class was relevant for antimicrobial use in feedlot medicine [55]. Each class was represented by AMR to one drug (see **Table 4**) with the most complete reference data set.
[2]Percentage of resistant isolates at each time point is a weighted average of prevalence values extracted from recent studies of healthy feedlot cattle in western Canada [9-10, 18-22].

Antimicrobial classes were selected for the calibration experiments if 1) the prevalence of detectable resistance was >1% at any of the time points, *and* 2) the class was relevant to products used for feedlot medicine (e.g., tetracyclines) [55]. The 1% threshold was used to distinguish antimicrobial classes where the change in detectable resistance over time was sufficient to model an association with AMU or contagious transmission. The 15- and 16-membered ring macrolides were treated as distinct/independent sub-classes given that their reference data sets were sufficiently dissimilar. Resistance breakpoints approved by the Clinical & Laboratory Standards Institute do not exist for two of the representative drugs selected for calibration, trimethoprim and sulfadimethoxine [56]; "resistance" as defined for these drugs refers to the percentage of isolates that grow at (i.e., are not inhibited by or are not susceptible to) the only tested concentrations (2 µg/mL and 256µg/ml, respectively) on the commercially available Bovine BOPO7F AST plate (ThermoFisher Scientific™). Based on their detectable prevalence in the reference data (>1%)

(**Figure 9**), the antimicrobial classes chosen for the initial calibration experiments were macrolides (15- and 16-membered ring), sulfonamides, trimethoprim and tetracyclines.

Configurations and Settings

Three model variants with unique structural configurations were each calibrated to the reference data for *M. haemolytica* and subsequently compared. In the first "antimicrobial use only" configuration, the potential for transmission between animals was disabled and could not affect the spread of detectable resistance; selection pressure from AMU was therefore the only driver for AMR in this scenario. The selection probability and waning rate parameters were varied in this configuration to find the best fit to the reference data.

In the second "transmission only" configuration, the potential for selection due to AMU was disabled and could not affect the emergence of detectable resistance; contagious transmission was therefore the only driver for AMR in this scenario. The waning rate, contact rate and stress effect multiplier parameters were varied in this configuration to find the best fit.

In the final "both antimicrobial use and transmission" configuration, both drivers were enabled and could jointly affect the emergence of detectable resistance. All of the selection probability, waning rate, contact rate and stress effect multiplier parameters were varied in this configuration to find the best fit.

The initialization and simulation settings for each calibration are detailed in **Table 3.** Automated calibration experiments using OptQuest's optimization tool were run in AnyLogic® 8 for n=2500 iterations (model runs with unique combinations of target parameters) with n=30 realizations (model runs that explore the extent of stochastic variation within each parameter set) per iteration. The number of iterations and realizations per iteration were selected to balance the competing priorities of methodological rigour (i.e., adequate assessment of stochastic variation for a specific vector of parameters) and computational efficiency to allow adequate exploration of the parameter space.

Table 3. Simulation parameters used to perform individual calibration experiments for each of the selected antimicrobial class and configuration combinations

	**Value for model calibrations**	**Possible alternative settings**	**Implication**
Initialization parameters			
Number of pens in feedlot	30	User selection	Modifiable setting based on feedlot size or desired balance of population size and computational efficiency
Number of animals per pen	200	User selection	Modifiable setting based on pen size or desired balance of pen density and computational efficiency
Proportion of high-risk animals in simulation	100%	0-100%	Modifiable setting based on risk level of incoming animals
Proportion of steers in simulation	100%	0-100%	Modifiable setting based on sex of incoming animals
Default metaphylactic drug selection	Tulathromycin (i.e., Draxxin)	Probability table, **see Figure S1**	Probability of metaphylactic drug selection can be fixed or vary by BRD risk level per empirical data
Simulation parameters			
Number of iterations (i.e., model runs with unique combinations of target parameters)	2500	User selection	Modifiable setting based on desired balance of adequate exploration of parameter space (i.e., methodological rigour) and computational efficiency
Number of realizations per iteration (i.e., model runs with identical combinations of target parameters but different "random seeds")	30	1	Modifiable setting based on desired balance of adequate exploration of stochastic variation within parameter set and computational efficiency
Optimization (objective) **criterion**	Minimize	Not applicable	The calibrated parameters are optimized (i.e., the best fit is achieved) when the objective function returns its smallest non-negative value
Optimization (objective) **function**	Compound function comprised of two components:<br>1) **curve fitness**, which quantifies the difference between linear datasets; and<br>2) **point fitness**, which quantifies the weighted difference between particular points in the datasets (1, 13, 70 and 170 DOF)	Not applicable	Function is the sum of the 1) average absolute difference between simulated and empirical datasets; and<br>2) the exponentiated absolute difference between specific reference points with stronger empirical data.<br><br>The point fitness weighting scheme can be modified to reflect variable user confidence in the empirical data points.

Objective Function

Each calibration experiment (n=15 initial combinations of 5 antimicrobial classes by 3 configurations) searched the parameter space defined by the minimum and maximum values in **Table 4** for the set of inputs that best reproduced the reference data. The best fit was achieved when the average of the objective functions across the realizations within an iteration returned its lowest non-negative value (i.e., when the difference between the simulated and empirical data was minimized by the optimizer). The compound objective function employed in this calibration was comprised of two components: 1) the curve fitness component, and 2) the point fitness component, which assigns greater weight or importance to reference points with stronger empirical data.

The components are summed as follows to calculate the objective value:

> root.getCurveFitness() + root.getPointPrevelanceFitness(**1.0**, 1.0) +
> root.getPointPrevelanceFitness(**13.0**, 1.0) + root.getPointPrevelanceFitness(**70.0**, 1.0) +
> root.getPointPrevelanceFitness(**170.0**, 1.0)

The curve fitness component applies the difference function in AnyLogic, which returns the square root of the average of the square of difference between linearly interpolated data sets, *R* (reference/empirical) and *M* (model/simulated). The point fitness component returns $\mathbf{e}^{(|\boldsymbol{r} - \boldsymbol{m} \mid \boldsymbol{w})}$ comparing two points in the *R* and *M* datasets (values *r* and *m,* respectively) with weight *w.* The resulting fit at day 1, 13 (if available), 70 and 170 DOF is assigned additional weight (*w* = 1.0) relative to 50 or 105 DOF given the recency and relevance of the data informing reference estimates for those time points.

Table 4. Best objective and calibrated parameter values from individual calibration experiments for each of the antimicrobial class and configuration combinations

Antimicrobial class	Reference drug belonging to class[1]	Scenario	Best objective value[2]	Selection probability, if applicable	Waning rate	Contact rate, if applicable	Stress effect multiplier, if applicable
				Mean probability per day	Mean rate per day	Mean rate per day	Unitless
15-membered ring macrolides	Tulathromycin	Drug use only	0.213	0.593	0.008	--	--
		Transmission only	**0.157**	**--**	**0.173**	**0.130**	**0.438**
		Both drug use/ transmission	0.186	0.625	1.118	1.163	0.119
16-membered ring macrolides	Tilmicosin	Drug use only	0.592	0.763	0.001	--	--
		Transmission only	0.391	--	1.449	1.600	0.026
		Both drug use/ transmission	**0.382**	**1.000**	**0.825**	**0.915**	**0.026**
Sulfonamides	Sulfadimethoxine	Drug use only	1.725	0.955	0.001	--	--
		Transmission only	0.154	--	2.113	1.299	0.685
		Both drug use/ transmission	**0.153**	**0.452**	**3.393**	**1.982**	**0.741**
Trimethoprim	Trimethoprim	Drug use only	0.039	1	0.013	--	--
		Transmission only	0.018	--	0.035	0.004	3.150
		Both drug use/ transmission	**0.017**	**0.829**	**0.058**	**0.026**	**0.459**
Tetracyclines	Oxytetracycline	Drug use only	0.337	0.134	0.005	--	--
		Transmission only	**0.291**	**--**	**0.011**	**0.005**	**0.623**
		Both drug use/ transmission	0.299	0.011	0.007	0.002	0.940
Cephalosporins	Ceftiofur	Both drug use/ transmission	0.001	0.001	2.763	0.379	0.667
Fluoroquinolones	Enrofloxacin	Both drug use/ transmission	0.002	0.025	0.610	0.001	0.046
Phenicols	Florfenicol	Both drug use/ transmission	0.007	0.002	0.014	0.001	3.038
**Fixed minimum value for calibration search function**				0.001	0.001/day	0.001/day	0
**Fixed maximum value for calibration search function**				1	5/day	2/day	10

[1]The antimicrobial drug with the most complete dataset (i.e., one or more raw data points for each time point) was selected to represent the entire class.
[2]The best (i.e., smallest) objective value was an average of the objective values from each realization (n=30) in the best iteration.

Model Output and Evaluation

The objective value for each iteration is an average of the objective values from each realization in the iteration (n=30). The calibrated parameters associated with the best iteration (i.e., the iteration with the smallest objective value) for each configuration/drug class combination are reported in **Table 4.** A systematic comparison of model "fit" for different configurations was

critical for examining our hypotheses about how detectable resistance emerges and spreads in the feedlot environment. The relative fits of candidate configurations (antimicrobial use only *vs.* transmission only *vs.* both antimicrobial use and transmission) for each antimicrobial class were evaluated by dividing the absolute differences between the objective values for each pair by the average for each pair. Percentage differences in excess of 20% indicated a substantial difference in model fit per the assessment criteria [57]; a cutoff of 20% is frequently used as the change-in-estimate criterion for identifying confounders in epidemiological studies and was adopted here. The results are reported in a difference matrix in **Table 5**.

For each of the antimicrobial classes, the "transmission only" and "both" configurations offer a substantially better fit to the empirical data than the "antimicrobial use only" variation. The percentage differences >20% in almost every case, with few exceptions; differences for "both" *vs.* "drug use only" for 15-membered ring macrolides and for "both" and "transmission only" *vs.* "drug use only" for tetracyclines do not reach the 20% threshold. The differences between best objective values for the "transmission only" and "both" configurations were less than substantial (<20%) for all classes.

Based on these findings, single calibration experiments for the "both" configuration were performed to estimate unknown parameters for antimicrobial classes with prevalences of resistance that did not exceed 1% in the empirical data (**Figure S9**), but are nevertheless important in feedlot medicine and specified as treatment options in the model. Parameters for cephalosporins (ceftiofur), fluoroquinolones (enrofloxacin), and phenicols (florfenicol) are reported in **Table 4**.

Table 5. Pairwise comparisons of best objective values from candidate scenarios for each antimicrobial class. The absolute difference between two objective values is divided by the average of those values to compare the ability of candidate scenarios to reproduce the reference data. Percentage differences exceeding 20% (shaded blue) indicate a substantial difference in model fit.

**Antimicrobial class**		15-membered ring macrolides			16-membered ring macrolides			Sulfonamides			Trimethoprim			Tetracyclines		
	**Scenario**	Drug use	Transmission	Both	Drug use	Transmission	Both	Drug use	Transmission	Both	Drug use	Transmission	Both	Drug use	Transmission	Both
15-membered ring macrolides	Drug use		**30.3%**	13.5%												
	Transmission	**-30.3%**		-16.9%												
	Both	-13.5%	16.9%													
16-membered ring macrolides	Drug use					**40.9%**	**43.1%**									
	Transmission				**-40.9%**		2.3%									
	Both				**-43.1%**	-2.3%										
Sulfonamides	Drug use								**167%**	**167%**						
	Transmission							**-167%**		0.6%						
	Both							**-167%**	-0.6%							
Trimethoprim	Drug use											**72.4%**	**78.6%**			
	Transmission										**-72.4%**		5.6%			
	Both										**-78.6%**	-5.6%				
Tetracyclines	Drug use														14.6%	11.9%
	Transmission													-14.6%		-2.7%
	Both													-11.9%	2.7%	

References

1. Grimm V, Berger U, Bastiansen F, Eliassen S, Ginot V, Giske J, et al. A standard protocol for describing individual-based and agent-based models. *Ecological Modelling.* (2006) 198:115-126.
2. Grimm V, Railsback SF, Vincenot CE, Berger U, Gallagher C, DeAngelis DL, et al. The ODD protocol for describing agent-based and other simulation models: a second update to improve clarity, replication and structural realism. *Journal of Artificial Societies and Social Simulation.* (2020) 23:7.
3. Taylor JD, Fulton RW, Lehenbauer TW, Step DL, Confer AW. The epidemiology of bovine respiratory disease: what is the evidence for predisposing factors? *Canadian Veterinary Journal.* (2010) 51;1095-1102.
4. Confer AW. Update on bacterial pathogenesis in BRD. *Animal Health Research Reviews.* (2009) 10:145-148.
5. Klima CL, Alexander TW, Hendrick S, McAllister TA. Characterization of *Mannheimia haemolytica* isolated from feedlot cattle that were healthy or treated for bovine respiratory disease. *Canadian Journal of Veterinary Research.* (2014) 78:38-45.
6. Brault SA, Hannon SJ, Gow SP, Warr BN, Withell J, Song J, Williams CM, Otto SJG, Booker CW, Morley PS. Antimicrobial use on 36 beef feedlots in western Canada: 2008-2012. *Frontiers in Veterinary Science.* (2019) 329.
7. Canadian Feedlot AMU/AMR Surveillance Program. Injectable Antimicrobial Use (AMU) in Canadian Feedlot Cattle 2019-2022 (Veterinarians). Available from: https://cfaasp.ca/resources/cfaasp-resources/Injectable-Antimicrobial-Use-AMU-In-2022-in-Canadian-Feedlot-Cattle-2019-2022-Veterinarians. Last accessed February 6, 2024.
8. Canadian Feedlot AMU/AMR Surveillance Program. In-Feed Antimicrobial Use (AMU) in Canadian Feedlot Cattle 2019-2022 (Veterinarians). Available from: https://cfaasp.ca/resources/cfaasp-resources/In-Feed-Antimicrobial-Use-AMU-In-2022-in-Canadian-Feedlot-Cattle-2019-2022-Veterinarians. Last accessed June 5, 2024.
9. Abi Younes J, Ramsay DE, Lacoste S, Deschner D, Hill JE, Campbell J, et al. Changes in the phenotypic susceptibility of *Mannheimia haemolytica* isolates to macrolide antimicrobials during the early feeding period following metaphylactic tulathromycin use in western Canadian feedlot calves. *Canadian Veterinary Journal.* (2022) 63:920-928.
10. Wennekamp TR, Waldner CL, Windeyer MC, Larson K, Trokhymchuk A, Campbell JR. Antimicrobial resistance in bovine respiratory disease: auction market- and ranch-raised calves. *Canadian Veterinary Journal.* (2022) 63:47-54.
11. O'Connor AM, Hu D, Totton SC, Scott N, Winder CB, Wang B, et al. A systematic review and network meta-analysis of injectable antibiotic options for control of bovine respiratory disease in the first 45 days post arrival at the feedlot. *Animal Health Research Reviews.* (2019) 20:163-181.
12. Alberta Cattle Feeders' Association. What goes on in a feedlot? Feedlot 101. Available from: https://cattlefeeders.ca/feedlot-101/. Last accessed February 7, 2024.
13. CanFax. CanFax 2020 Annual Report: CanFax Trends West Summary. Calgary, AB.
14. Zinn RA, Barreras A, Owens FN, Plascencia A. Performance by feedlot steers and heifers: daily gain, mature body weight, dry matter intake, and dietary energetics. *Journal of Animal Science* (2008) 86:2680-2689.
15. Erickson GE, Bremer VR, Klopfenstein TJ, Smith DR, Hanford KJ, Peterson RE, et al. Relationship between morbidity and performance in feedlot cattle. (2011). *Nebraska Beef Cattle Reports.* 608. Available from: https://digitalcommons.unl.edu/cgi/animalscinbcr/608.
16. Kruse GT, Randle RR, Hostetler DE, Tibbetts GK, Griffin DD, Hanford KJ, et al. The effect of lameness on average daily gain in feedlot steers. (2013). *Nebraska Beef Cattle Reports.* 731. Available from: https://digitalcommons.unl.edu/cgi/animalscinbcr/731.
17. Agriculture and Agri-Food Canada. Red meat and livestock slaughter and carcass weights. Available from: https://agriculture.canada.ca/en/sector/animal-industry/red-meat-and-livestock-market-information/slaughter-and-carcass-weights. Accessed February 7, 2024.

18. Noyes NR, Benedict KM, Gow SP, Booker CW, Hannon SJ, McAllister TA, et al. *Mannheimia haemolytica* in feedlot cattle: prevalence of recovery and associations with antimicrobial use, resistance, and health outcomes. *Journal of Veterinary Internal Medicine.* (2015) 29:705-713.
19. Erickson NEN, Ngeleka MG, Lubbers BV, Trokhymchuk A. Changes in the rates of field isolation and antimicrobial susceptibility of bacterial pathogens collected from fall-placed feedlot steers between arrival at the feedlot and 90 to 120 days on feed. *The Bovine Practitioner.* (2017) 51:165-173.
20. Timsit E, Hallewell J, Booker C, Tison N, Amat S, Alexander TW. Prevalence and antimicrobial susceptibility of *Mannheimia haemolytica*, *Pasteurella multocida,* and *Histophilus somni* isolated from the lower respiratory tract of healthy feedlot cattle and those diagnosed with bovine respiratory disease. *Veterinary Microbiology.* (2017) 208:118-125.
21. Andrés-Lasheras S, Ha R, Zaheer R, Lee C, Booker CW, Dorin C, et al. Prevalence and risk factors associated with antimicrobial resistance in bacteria related to bovine respiratory disease – a broad cross-sectional study of beef cattle at entry into Canadian feedlots. *Frontiers in Veterinary Science.* (2021) 8:710.
22. Gow S, Bergen R, Booker C, Butters A, Dorin C, Dimmers G, et al. National surveillance of antimicrobial use and antimicrobial resistance in Canadian feedlots. *American Association of Bovine Practitioners Conference Proceedings.* (2021) 54:34-41. Available from: https://bovine-ojs-tamu.tdl.org/AABP/article/view/8291.
23. Cortes JA, Hendrick S, Janzen E, Pajor EA, Orsel K. Economic impact of digital dermatitis, foot rot, and bovine respiratory disease in feedlot cattle. *Translational Animal Science* (2021) 5.
24. Hilton WM. BRD in 2014: where have we been, where we are now, and where do we want to go? *Animal Health Research Reviews.* 2014;15(2).
25. Knight GM, Davies NG, Colijn C, Coll F, Donker T, Gifford DR, et al. Mathematical modelling for antibiotic resistance control policy: do we know enough? *BMC Infectious Diseases.* (2019) 19:1011.
26. Food and Agriculture Organization of the United Nations. Drivers, dynamics and epidemiology of antimicrobial resistance in animal production. 2016. Available from: https://www.fao.org/3/i6209e/i6209e.pdf.
27. Drugs.com. Zactran Injectable Solution (Canada). Available from: https://www.drugs.com/vet/zactran-injectable-solution-can.html. Last accessed February 7, 2024.
28. Drugs.com. Zuprevo (Canada). Available from: https://www.drugs.com/vet/zuprevo-can.html. Last accessed February 7, 2024.
29. Modric S, Webb AI, Derendorf H. Pharmacokinetics and pharmacodynamics of tilmicosin in sheep and cattle. *Journal of Veterinary Pharmacology and Therapeutics.* (1998) 21:444-452.
30. Kaartinen L, Gips M, Laurila T, Härtel H, Soback S, Pyörälä S. Pharmacokinetics of sulphadoxine and trimethoprim and tissue irritation caused by two sulphadoxine-trimethoprim containing products after subcutaneous administration in pre-ruminant calves. *Veterinary Research.* (2000) 31:517-526.
31. The United States Pharmacopeial Corporation. Penicillin G (Veterinary – Systemic). 2007. Available from: https://cdn.ymaws.com/www.aavpt.org/resource/resmgr/imported/penicillinG.pdf. Last accessed February 7, 2024.
32. Reinbold JB, Coetzee JF, Gehring R, Havel JA, Hollis LC, Olson KC, et al. Plasma pharmacokinetics of oral chlortetracycline in group fed, ruminating, Holstein steers in a feedlot setting. *Journal of Veterinary Pharmacology and Therapeutics.* (2010) 33:76-83.
33. Lacroix MZ, Gayrard-Troy VV, Picard-Hagen N, Toutain PL. Comparative bioavailability between two routes of administration of florfenicol and flunixin in cattle. *Revue de Médecine Vétérinaire, Ecole Nationale Vétérinaire de Toulouse.* (2011) 162:321-324.
34. Papich MG. Saunders Handbook of Veterinary Drugs: small and large animal, 4th edition. (2016) St. Louis, Missouri: Elsevier.
35. Trouchon T, Lefebvre S. A review of enrofloxacin for veterinary use. *Open Journal of Veterinary Medicine.* (2016) 6:40-58.

36. Foster DM, Jacob ME, Farmer KA, Callahan BJ, Theriot CM, Kathariou S, et al. Ceftiofur formulation differentially affects the intestinal drug concentration, resistance of fecal *Escherichia coli,* and the microbiome of steers. *PLoS One.* (2019) 14:e0223378.
37. Brault SA, Hannon SJ, Gow SP, Otto SJG, Booker CW, Morley PS. Calculation of antimicrobial use indicators in beef feedlots-effects of choice of metric and standardized values. *Frontiers in Veterinary Science.* (2019) 6.
38. Zaheer R, Cook SR, Klima CL, Stanford K, Alexander T, Topp E, et al. Effect of subtherapeutic vs. therapeutic administration of macrolides on antimicrobial resistance in *Mannheimia haemolytica* and enterococci isolated from beef cattle. *Frontiers in Microbiology.* (2013) 4:133.
39. Callan RJ, Garry FB. Biosecurity and bovine respiratory disease. *Veterinary Clinics of North America: Food Animal Practice.* (2002) 18:57-77.
40. Babcock AH, Renter DG, White BJ, Dubnicka SR, Scott HM. Temporal distributions of respiratory disease events within cohorts of feedlot cattle and associations with cattle health and performance indices. *Preventive Veterinary Medicine.* (2010) 97:198-219.
41. Compendium of Veterinary Products, 2021. Available from: https://bayerall.cvpservice.com/. Last accessed February 7, 2024.
42. Briggs AH, Weinstein MC, Fenwick EAL, Karnon J, Sculpher MJ, Paltiel AD. Model parameter and uncertainty analysis: a report of the ISPOR-SMDM Modeling Good Research Practices Task Force Working Group-6. *Value in Health.* (2012) 15:835-842.
43. Kleijnen JPC, Wan J. Optimization of simulated systems: OptQuest and alternatives. *Simulation Modelling Practice and Theory.* (2007) 15:354-362.
44. Laguna M. OptQuest of Complex Systems. Boulder, CO: OptTek Systems, Inc (2011).
45. OptTek Systems, Inc. OptQuest. (2022). Available from: https://www.opttek.com/products/optquest/. Last accessed February 7, 2024.
46. Osgood N, Liu J. Combining Markov chain Monte Carlo approaches and dynamic modeling. (2015) In: H. Rahmandad, editor, Analytical methods for dynamic modelers. MIT Press, Cambridge, MA. p. 125-170.
47. Osgood N, Liu J, Dueck S. Combining MCMC and Compartmental Modeling to Enhance Understanding of Chlamydia Control in Saskatchewan. (2014) In: 42nd Annual Meeting of the Statistical Society of Canada, Toronto, ON.
48. Safarishahrbijari A, Teyhouee A, Waldner C, Liu J, Osgood ND. Predictive accuracy of particle filtering in dynamic models supporting outbreak projections. *BMC Infectious Diseases* 17:648.
49. Li X, Keeler B, Zahan R, Duan L, Safarishahrbijari A, Goertzen J, et al. Illuminating the Hidden Elements and Future Evolution of Opioid Abuse Using Dynamic Modeling, Big Data and Particle Markov Chain Monte Carlo. (2018). In: 11th International Conference on Social, Cultural, and Behavioral Modeling (SBP-BRiMS), Washington, DC. p 101-110.
50. Duan L, Osgood N. GPU Accelerated PMCMC Algorithm with System Dynamics Modelling. (2021). In: International Conference on Social Computing, Behavioral-Cultural Modeling and Prediction and Behavior Representation in Modeling and Simulation. p 101-110.
51. Camanes G, Joly A, Fourichon C, Ben Romdhane R, Ezanno P. Control measures to prevent the increase of paratuberculosis prevalence in dairy cattle herds: an individual-based modelling approach. *Veterinary Research.* (2018) 49:60.
52. Lamperti F, Roventini A, Sani A. Agent-based model calibration using machine learning surrogates. *Journal of Economic Dynamics and Control.* (2018) 90:366-389.
53. Smith NR, Trauer JM, Gambhir M, Richards JS, Maude RJ, Keith JM, Flegg JA. Agent-based models of malaria transmission: a systematic review. *Malaria Journal.* (2018) 17:299.
54. Platt D. A comparison of economic agent-based model calibration methods. *Journal of Economic Dynamics and Control* (2020) 113:103859.
55. Canadian Feedlot AMU/AMR Surveillance Program. Antimicrobial and Antibiotic Backgrounder. Available from: https://cfaasp.ca/resources/cfaasp-resources/antimicrobial-and-antibiotic-backgrounder. Last accessed February 7, 2024.
56. Clinical & Laboratory Standards Institute. Performance Standards for Antimicrobial Disk and Dilution Susceptibility Tests for Bacteria Isolated from Animals. (2020) CLSI Guideline VET01S. 5th edition. Wayne, PA.

57. Dohoo IR, Martin W, Stryhn HE. Veterinary epidemiologic research. (2003) Charlottetown, PEI: University of Prince Edward Island.
58. Hannon SJ, Brault SA, Otto SJG, Morley PS, McAllister TA, Booker CW, et al. Feedlot cattle antimicrobial use surveillance network: a Canadian journey. *Frontiers in Veterinary Science.* (2020) 20:e596042.
59. Apley MD. Diagnosis and therapy of feedlot lameness. *American Association of Bovine Practitioners Conference Proceedings.* (2020) 53:54-59. Available from: https://bovine-ojs-tamu.tdl.org/AABP/article/view/7968.

Supplementary Figures and Tables

Figure S1. Probability of metaphylactic drug choice based on level of BRD risk at feedlot entry[1]. Protocol by risk group[2] can be probabilistically selected at model initialization and applies to all animals of that type in the feedlot for a unique model run.

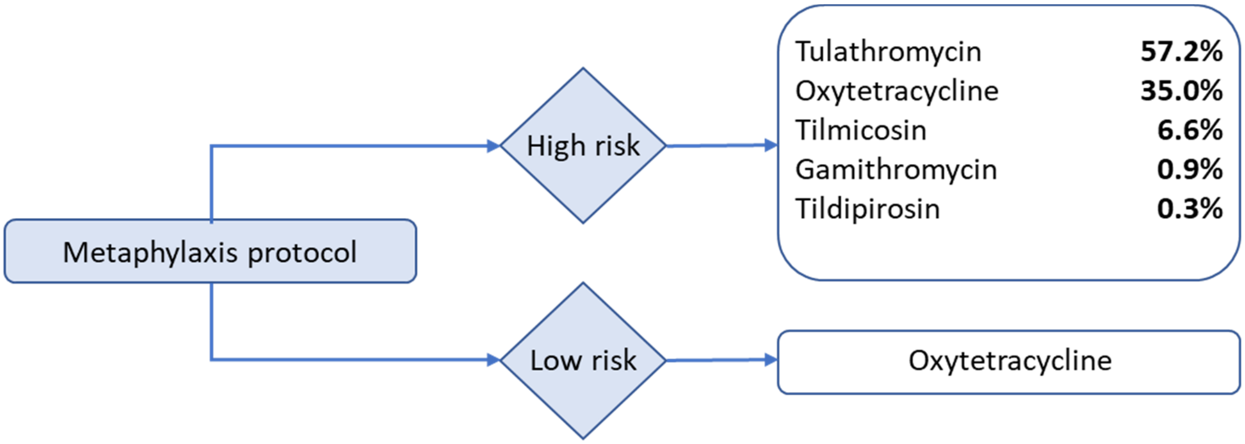

[1]Probabilities were derived from a sub-analysis of antimicrobial use trends on western Canadian feedlots [6]. Historical use data were assembled from the literature to correspond with the timing of antimicrobial resistance data used in the model calibration experiments, and are not an endorsement of any particular regimen. Gamithromycin is no longer available in Canada.

[2]BRD risk is determined by animal weight at feedlot arrival [12-13]; in future experiments with the model, the infrastructure exists to incorporate a mixture of risks based on factors including animal sex, origin, and vaccination status.

Figure S2. Probability and duration of prophylactic (i.e., in-feed) protocols for the prevention of histophilosis and liver abscesses[1]. Protocol for each indication can be probabilistically selected at model initialization and applies to all pens in the feedlot for a unique model run.

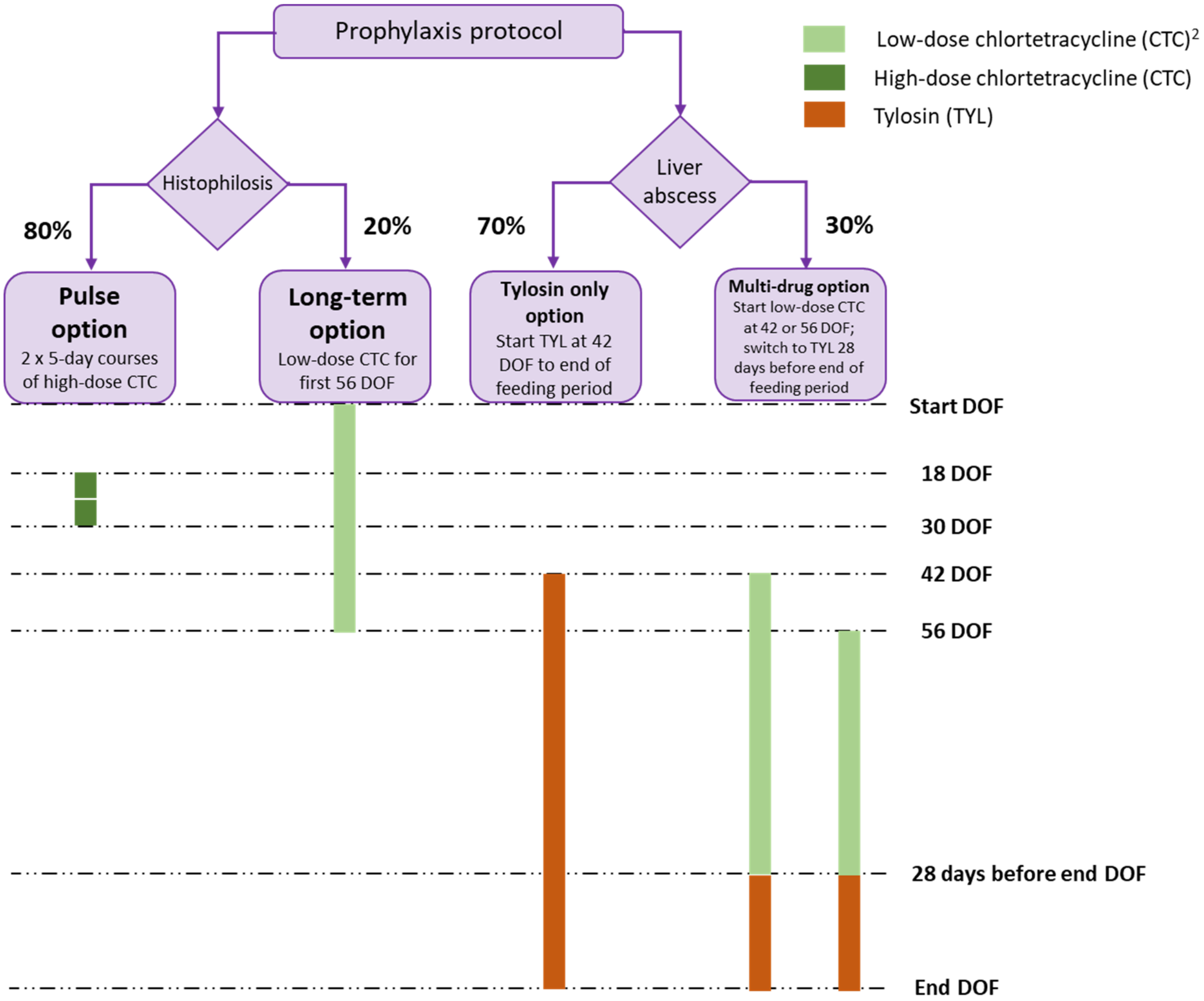

[1]Probabilities were estimated from sub-analyses of antimicrobial use trends on western Canadian feedlots [6], antimicrobial use data collected by the Canadian Integrated Program for Antimicrobial Resistance Surveillance [referenced in Hannon et al. [58]], and a series of expert interviews with feedlot veterinarians. Historical use data were assembled from the literature to correspond with the timing of antimicrobial resistance data used in the model calibration experiments, and are not an endorsement of any particular regimen.

[2]Chlortetracycline (CTC) are administered prophylactically at both "low" and "high" dosages (indicated above with the light and dark green bars, respectively). When the "low dose" of CTC is used, the calibrated "selection probability" for tetracyclines is adjusted by a multiplier (0.2) that reflects the average concentration of that regimen relative to the "high dose" regimen.

Figure S3. Probability of therapeutic drug choice for first and subsequent BRD diagnoses based on 1) level of BRD risk at feedlot entry[1] and 2) animal weight at time of infection. Protocol by risk[2] and weight group can be probabilistically selected at model initialization and applies to all animals in the feedlot for a unique model run.

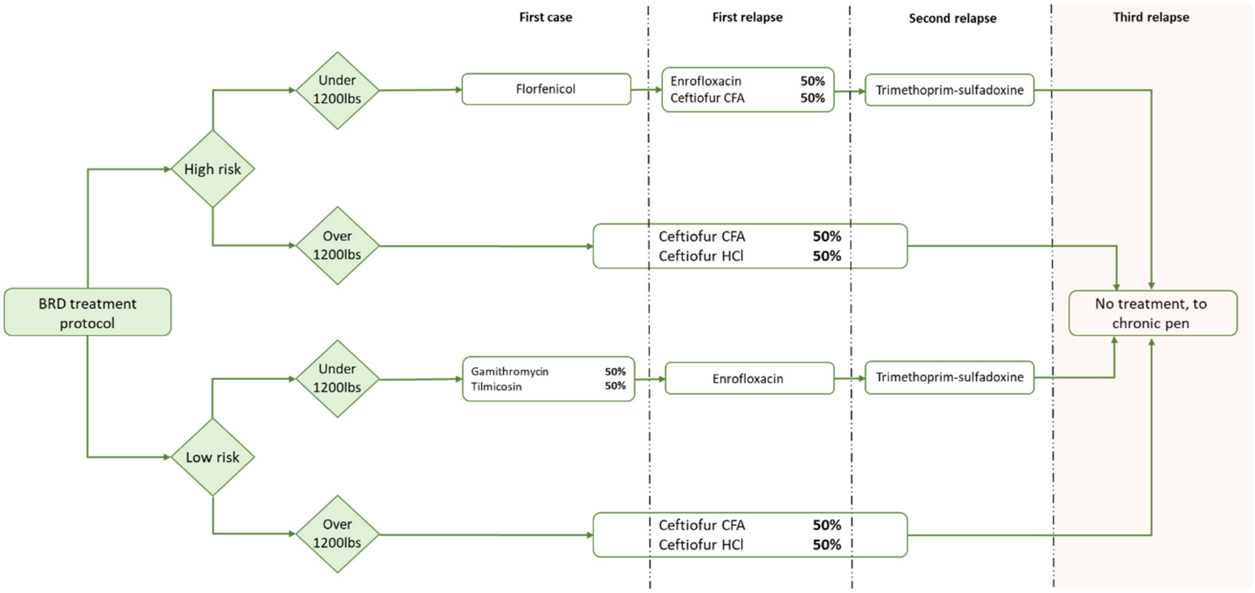

[1]BRD risk is determined by animal weight at feedlot arrival [12-13]; in future experiments with the model, the infrastructure exists to incorporate a mixture of risks based on factors including animal sex, origin, and vaccination status.
[2]BRD treatment protocols were developed following a series of expert interviews with feedlot veterinarians. Historical use data were assembled from the literature to correspond with the timing of antimicrobial resistance data used in the model calibration experiments, and are not an endorsement of any particular regimen. Gamithromycin is no longer available in Canada.

Figure S4. Probability of therapeutic drug choice and location of therapy for first and subsequent arthritis diagnoses based on animal weight at time of diagnosis[1]. Protocol by weight group can be probabilistically selected at model initialization and applies to all animals in the feedlot for a unique model run.

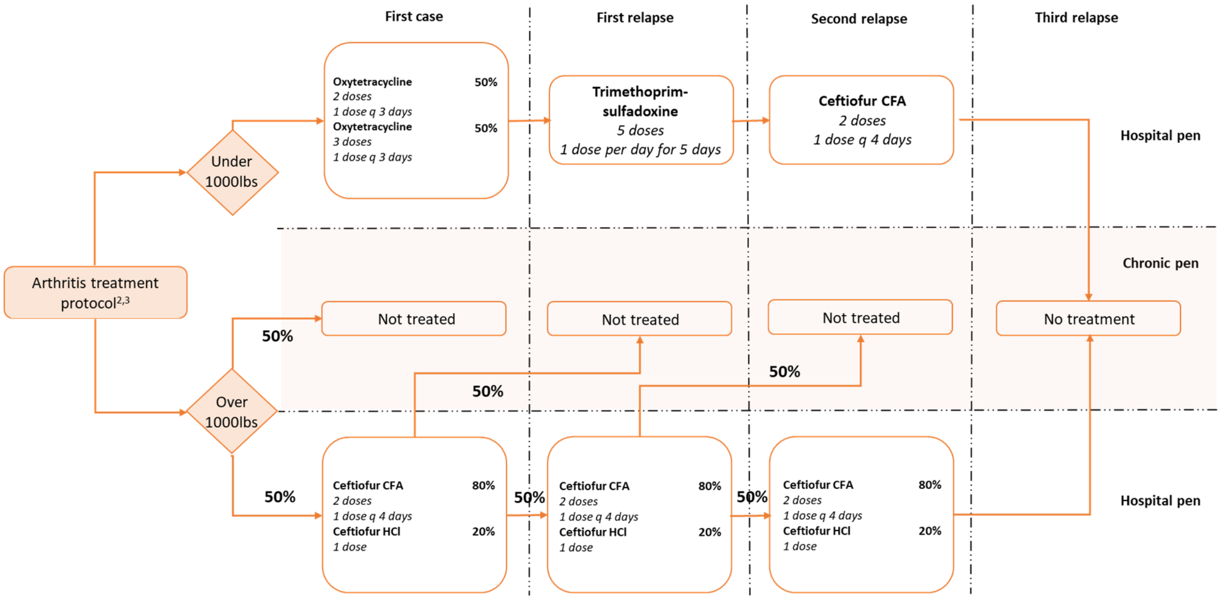

"q" = "every" in the above figure

[1]Arthritis treatment protocols were developed following a series of expert interviews with feedlot veterinarians. Historical use data were assembled from the literature to correspond with the timing of antimicrobial resistance data used in the model calibration experiments, and are not an endorsement of any particular regimen. Gamithromycin is no longer available in Canada.

[2]Animals that are probabilistically selected for arthritis treatment are relocated to the hospital pen and administered antimicrobials as part of multi-day regimens rather than single exposures; calves in the hospital pen are temporarily assigned an ADG equal to zero.

[3]Ceftiofur is not expected to be effective against uncomplicated *Mycoplasma bovis*-associated arthritis [59]. However, arthritis diagnosed late in the feeding period in fall-placed calves can be confounded by other infectious agents, and some cases are likely sequelae of chronic and unresponsive foot rot.

Figure S5. **Probability of therapeutic drug choice for foot rot diagnosis based on animal weight at time of infection[1].** Protocol by weight group can be probabilistically selected at model initialization and applies to all animals in the feedlot for a unique model run.

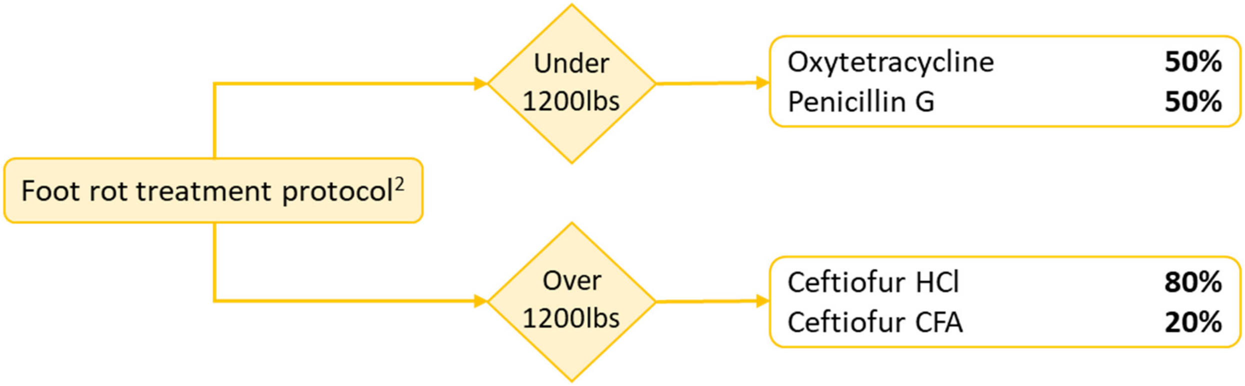

[1]Foot rot treatment protocols were developed following a series of expert interviews with feedlot veterinarians. Historical use data were assembled from the literature to correspond with the timing of antimicrobial resistance data used in the model calibration experiments, and are not an endorsement of any particular regimen.

[2]An "outbreak protocol" is triggered if 10% or more of the animals in a shared pen are diagnosed with foot rot in a single feeding period; in this scenario, a 7-day course of high-dose chlortetracycline (CTC) is administered at the pen-level (i.e., in-feed) to control the outbreak/prevent additional cases.

Figure S6. State charts for location (L) and mortality (R) in the Cattle agent. (L): Calves being treated for arthritis are moved to the hospital pen for the duration of their treatment; calves that are not expected to respond to further treatment are moved to the chronic pen. Transition arrows demarcated by “envelope” symbols depend on the receipt of a “move to pen” message. **(R):** Calves can die from BRD, histophilosis or other causes in the model. Transition arrows demarcated by “graph” symbols are those governed by daily mortality rates derived from empirical data. Living calves periodically update their hazard rates according to the days on feed (DOF) and their condition.

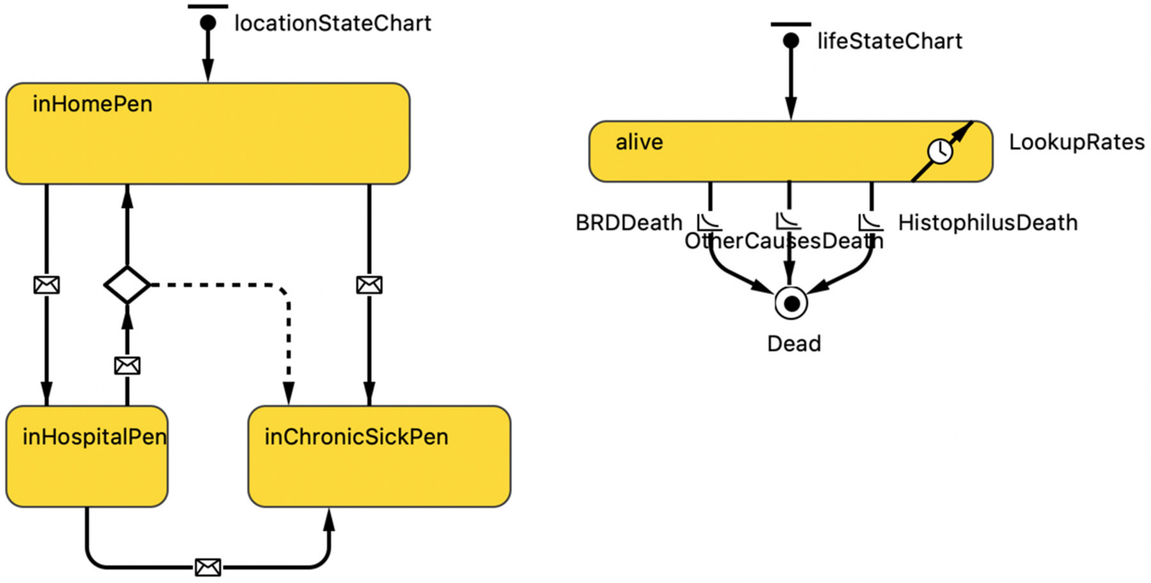

Figure S7. State chart for Treatment agent. The treatment agent administers a specific antimicrobial to a particular animal as outlined in the AMU protocols (**Figures S1-S5**). Treatments transition to the withdrawal state in one of two ways: 1) a prescribed amount of time has elapsed (arrow demarcated by a "clock" symbol), or 2) the animal reaches a threshold weight (arrow demarcated by a "question mark" symbol).

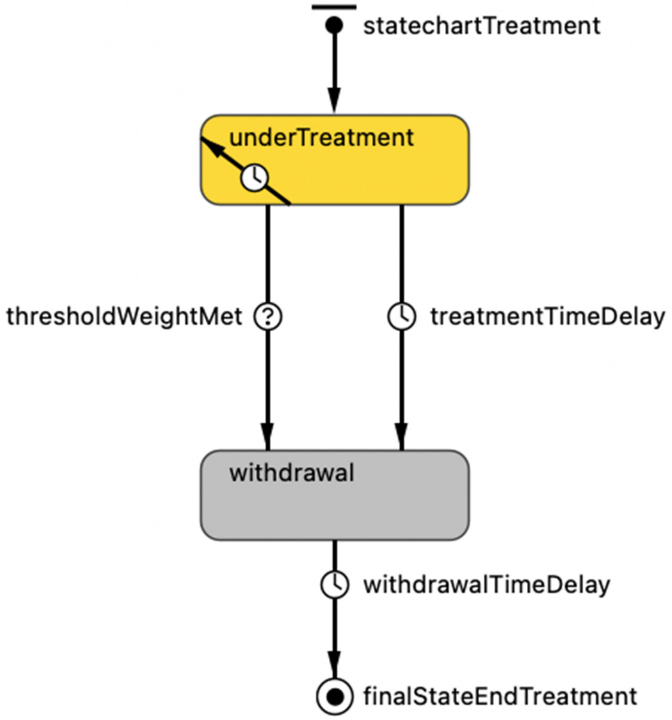

Figure S8. Matrix describing the linkages between drugs belonging to the same antimicrobial class or sub-class. Drugs from the same class were assumed to be equally vulnerable to the relevant resistance mechanism; co-selection, co-waning and co-transmission of detectable resistance was thus observed for fully linked (=1) drugs (**Figure 7**). Drugs from different classes were assumed to be selected and transmitted independently of each other.

**Antimicrobial drug (active ingredient)**	Ceftiofur CFA	Ceftiofur HCl	Enrofloxacin	Tulathromycin	Gamithromycin	Tilmicosin	Tildipirosin	Tylosin	Sulfadoxine	Trimethoprim	Ampicillin	Penicillin	Spectinomycin	Florfenicol	Florfenicol/ flunixin	Oxytetracycline	Chlortetracycline (high dose)	Chlortetracycline (low dose)
Ceftiofur CFA	**1.00**	**1.00**	0.00	0.00	0.00	0.00	0.00	0.00	0.00	0.00	0.00	0.00	0.00	0.00	0.00	0.00	0.00	0.00
Ceftiofur HCl	**1.00**	**1.00**	0.00	0.00	0.00	0.00	0.00	0.00	0.00	0.00	0.00	0.00	0.00	0.00	0.00	0.00	0.00	0.00
Enrofloxacin	0.00	0.00	**1.00**	0.00	0.00	0.00	0.00	0.00	0.00	0.00	0.00	0.00	0.00	0.00	0.00	0.00	0.00	0.00
Tulathromycin[1]	0.00	0.00	0.00	**1.00**	**1.00**	0.00	0.00	0.00	0.00	0.00	0.00	0.00	0.00	0.00	0.00	0.00	0.00	0.00
Gamithromycin[1]	0.00	0.00	0.00	**1.00**	**1.00**	0.00	0.00	0.00	0.00	0.00	0.00	0.00	0.00	0.00	0.00	0.00	0.00	0.00
Tilmicosin[1]	0.00	0.00	0.00	0.00	0.00	**1.00**	**1.00**	0.00	0.00	0.00	0.00	0.00	0.00	0.00	0.00	0.00	0.00	0.00
Tildipirosin[1]	0.00	0.00	0.00	0.00	0.00	**1.00**	**1.00**	0.00	0.00	0.00	0.00	0.00	0.00	0.00	0.00	0.00	0.00	0.00
Tylosin (in-feed)[2]	0.00	0.00	0.00	0.00	0.00	0.00	0.00	**1.00**	0.00	0.00	0.00	0.00	0.00	0.00	0.00	0.00	0.00	0.00
Sulfadoxine[3]	0.00	0.00	0.00	0.00	0.00	0.00	0.00	0.00	**1.00**	0.00	0.00	0.00	0.00	0.00	0.00	0.00	0.00	0.00
Trimethoprim[3]	0.00	0.00	0.00	0.00	0.00	0.00	0.00	0.00	0.00	**1.00**	0.00	0.00	0.00	0.00	0.00	0.00	0.00	0.00
Ampicillin	0.00	0.00	0.00	0.00	0.00	0.00	0.00	0.00	0.00	0.00	**1.00**	**1.00**	0.00	0.00	0.00	0.00	0.00	0.00
Penicillin G	0.00	0.00	0.00	0.00	0.00	0.00	0.00	0.00	0.00	0.00	**1.00**	**1.00**	0.00	0.00	0.00	0.00	0.00	0.00
Spectinomycin	0.00	0.00	0.00	0.00	0.00	0.00	0.00	0.00	0.00	0.00	0.00	0.00	**1.00**	0.00	0.00	0.00	0.00	0.00
Florfenicol	0.00	0.00	0.00	0.00	0.00	0.00	0.00	0.00	0.00	0.00	0.00	0.00	0.00	**1.00**	**1.00**	0.00	0.00	0.00
Florfenicol/flunixin	0.00	0.00	0.00	0.00	0.00	0.00	0.00	0.00	0.00	0.00	0.00	0.00	0.00	**1.00**	**1.00**	0.00	0.00	0.00
Oxytetracycline	0.00	0.00	0.00	0.00	0.00	0.00	0.00	0.00	0.00	0.00	0.00	0.00	0.00	0.00	0.00	**1.00**	**1.00**	**1.00**
Chlortetracycline (high dose)[4]	0.00	0.00	0.00	0.00	0.00	0.00	0.00	0.00	0.00	0.00	0.00	0.00	0.00	0.00	0.00	**1.00**	**1.00**	**1.00**
Chlortetracycline (low dose)[4]	0.00	0.00	0.00	0.00	0.00	0.00	0.00	0.00	0.00	0.00	0.00	0.00	0.00	0.00	0.00	**1.00**	**1.00**	**1.00**

[1]15-membered ring macrolides tulathromycin and gamithromycin were independent from 16-membered ring macrolides tilmicosin and tildipirosin (=0) in the initial calibration experiments.
[2]Prophylactic (i.e., in-feed) tylosin use does not co-select for resistance to injectable 16-membered ring macrolides in its sub-class (=0), consistent with the observation that "the in-feed levels of tylosin [have] no effect on the prevalence of *M. haemolytica"* [38].
[3]The use of potentiated sulfonamide Trivetrin (see **Table 2**) in the model triggered the individual "selection probabilities" per day for both trimethoprim and sulfonamides, but resistances to these classes are independent and do not co-select, co-wane or co-transmit (=0).
[4]Chlortetracycline at both the "high" (i.e., therapeutic) and "low" (i.e., prophylactic) dosages were fully linked to each other and to oxytetracycline (=1). When the "low dose" of chlortetracycline was used, the calibrated "selection probability" for tetracyclines was adjusted by a multiplier (0.2) that reflected the average concentration of that regimen relative to the "high dose" regimen. The multiplier was estimated from AMU data collected by the Canadian Integrated Program for Antimicrobial Resistance Surveillance [referenced in Hannon et al. [58]] and a series of expert interviews with feedlot veterinarians.

Figure S9. Percentage of *Mannheimia haemolytica* isolates with detectable phenotypic resistance to antimicrobial classes of interest *not selected for calibration*[1] over the feeding period. The custom longitudinal dataset was synthesized from recent empirical studies of antimicrobial resistance prevalence in western Canadian feedlot cattle from mixed origins[2].

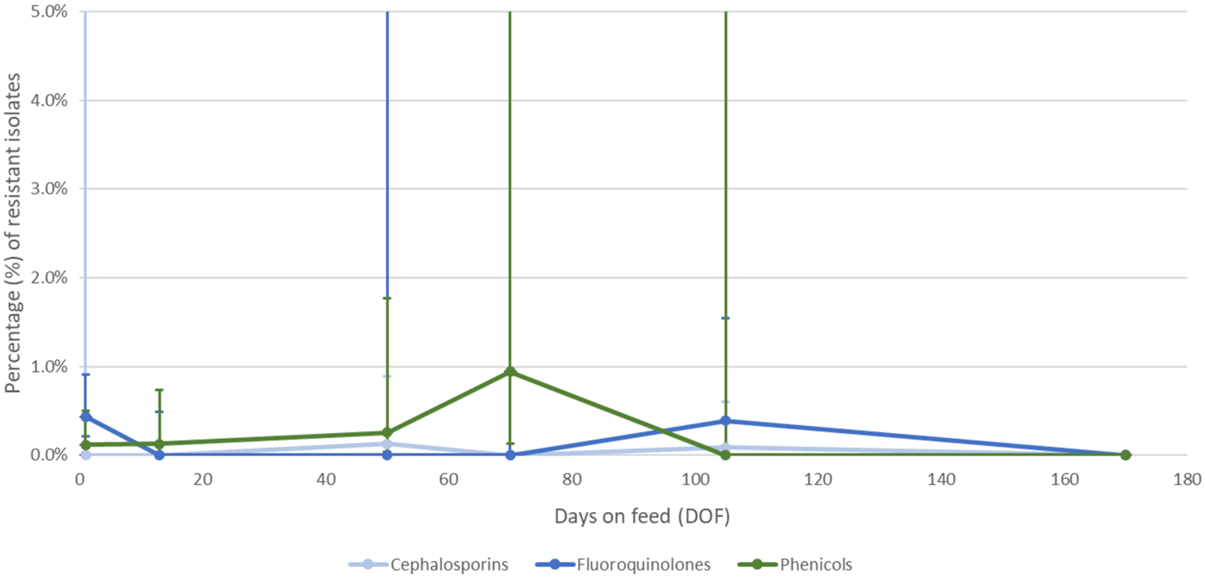

[1]Antimicrobial classes were selected for calibration if the prevalence of detectable resistance was >1% at any time point **and** the class was relevant for antimicrobial use in feedlot medicine [55]. Each class was represented by AMR to one drug (see **Table 4**) with the most complete reference data set.

[2]Percentage of resistant isolates at each time point is a weighted average of prevalence values extracted from recent studies of healthy feedlot cattle in western Canada [9-10, 18-22].
