# Supplementary material for: Contagious acquisition of antimicrobial resistance is critical for explaining emergence in western Canadian feedlots—insights from an agent-based modelling tool
Source: Front Vet Sci. 2025 Jan 10;11:1466986. doi: 10.3389/fvets.2024.1466986 (PMC11758982; doi:10.3389/fvets.2024.1466986)
Supplement: Supplementary file 2 [file Data_Sheet_2.pdf]

Supplementary figures and tables

Contagious acquisition of antimicrobial resistance is critical for explaining emergence in western Canadian feedlots: Insights from an agent-based modelling tool

Dana Ramsay, Department of Large Animal Clinical Sciences, Western College of Veterinary Medicine, University of Saskatchewan, Saskatoon, SK, Canada

Wade McDonald, Department of Computer Science, University of Saskatchewan, Saskatoon, SK, Canada

Michelle Thompson, Department of Large Animal Clinical Sciences, Western College of Veterinary Medicine, University of Saskatchewan, Saskatoon, SK, Canada

Nathan Erickson, Department of Large Animal Clinical Sciences, Western College of Veterinary Medicine, University of Saskatchewan, Saskatoon, SK, Canada

Sheryl Gow, Canadian Integrated Program for Antimicrobial Resistance Surveillance, Public Health Agency of Canada, Saskatoon, SK, Canada

Nathaniel Osgood, Department of Computer Science, University of Saskatchewan, Saskatoon, SK, Canada

Cheryl Waldner, Department of Large Animal Clinical Sciences, Western College of Veterinary Medicine, University of Saskatchewan, Saskatoon, SK, Canada (cheryl.waldner@usask.ca)

Figure S1. Process of calf management for each home pen in the agent-based model. Transition arrows demarcated by "clock" symbols are processes linked to a particular length of time (e.g., the number of days on a certain feed type). Transition arrows demarcated by "question mark" symbols are processes triggered when a specific condition is met (e.g., the emptying of occupied pens when the average target weight is reached). The branch demarcated by a "diamond" symbol indicates the possibility of two outcomes, where 1) no metaphylaxis (default, dotted line) or 2) injectable metaphylaxis (solid line) as assigned at the feedlot level at model initialization.

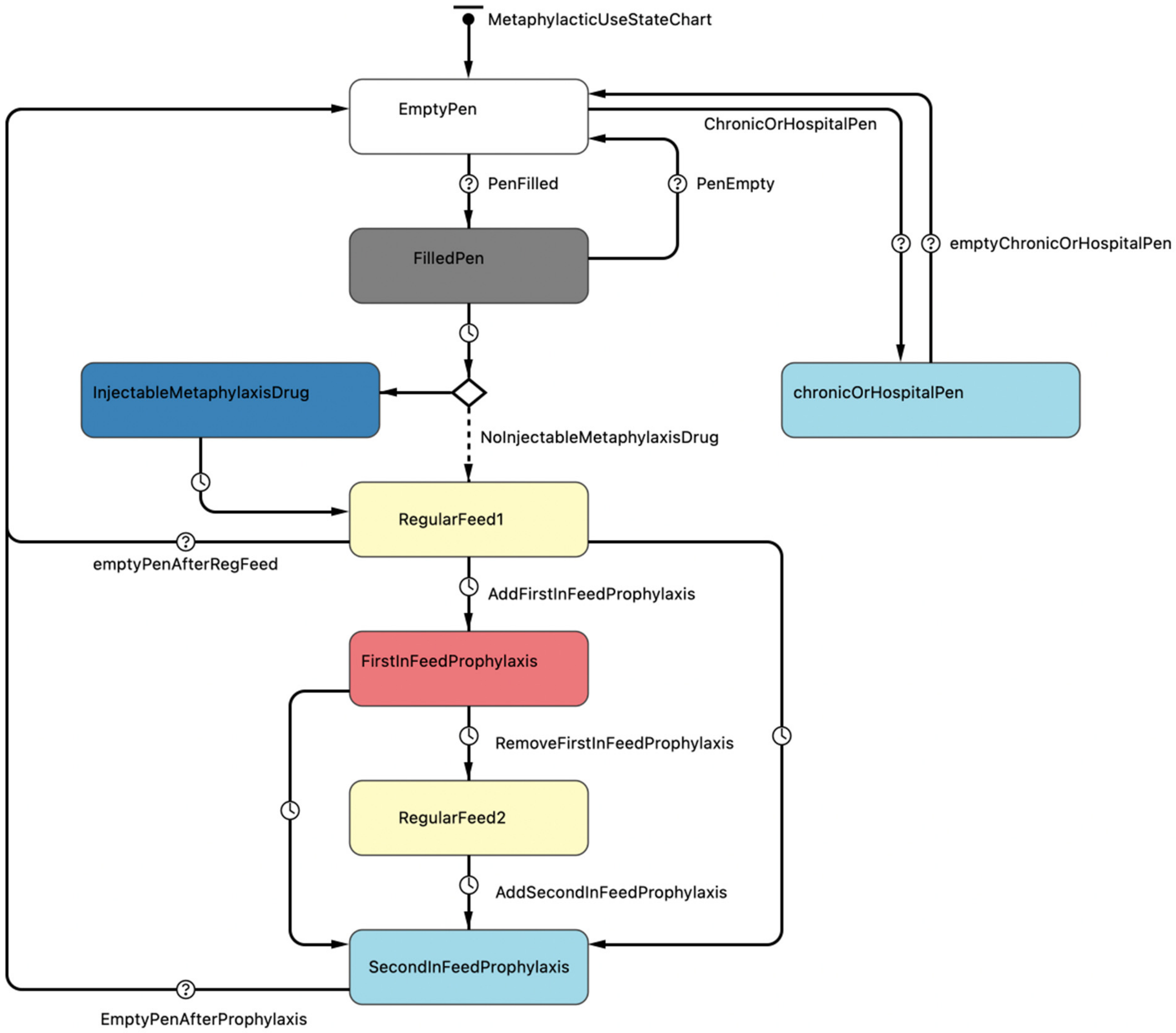

Figure S2. Representation of animal health status for each calf in the agent-based model. The "disease" states are mutually exclusive, and an animal can only receive treatment for one disease at any given time. Transition arrows demarcated by "graph" symbols are those governed by daily hazard rates derived from empirical data. Transition arrows demarcated by "envelope" symbols depend on the receipt of an "end treatment" message. The length of treatment refers to the dosing interval (for multi-day regimens) and therapeutic interval (estimated period of selective pressure following dosing interval). Branches demarcated by "diamond" symbols indicate the possibility of two outcomes, including 1) treatment success and return to healthy state (default) or 2) baseline (fixed probability for BRD, arthritis) or resistance-linked (BRD only, when enabled) treatment failure and return to disease state (termed a "relapse" for the purposes of this description).

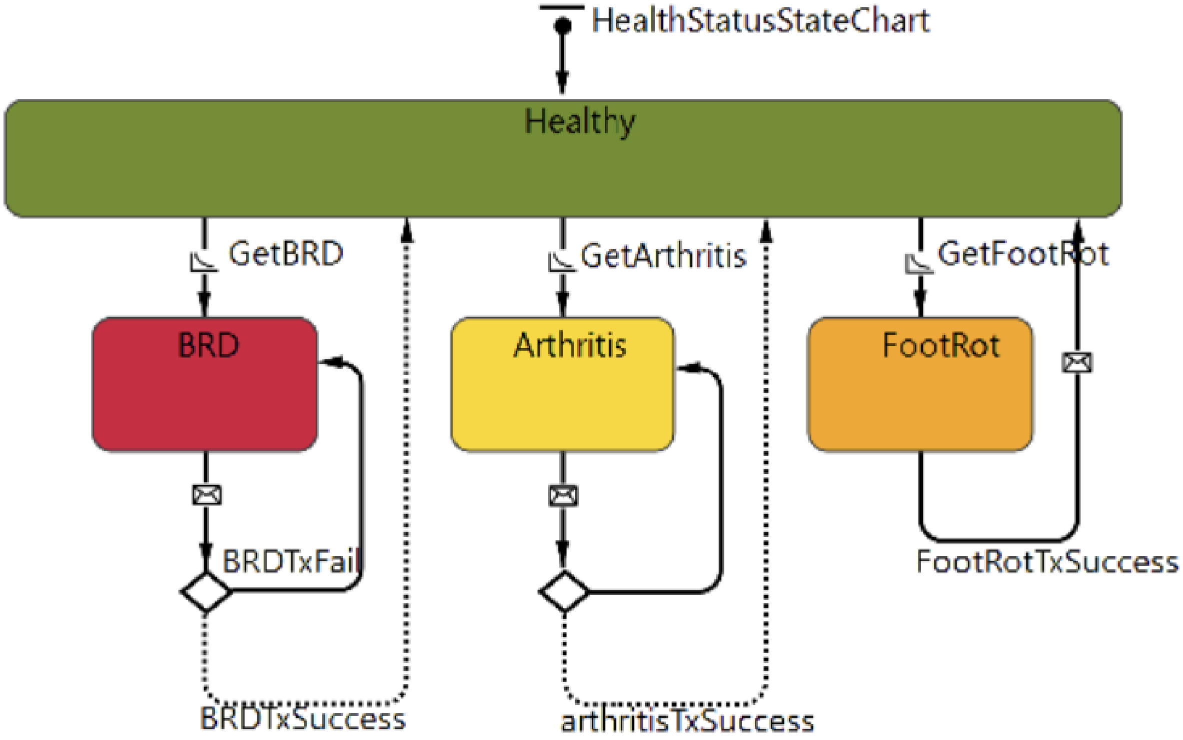

Figure S3. Probability of metaphylactic drug choice based on level of BRD risk at feedlot entry[1]. Protocol by risk group[2] was probabilistically selected at model initialization for calibration and Monte-Carlo experiments and applied to all animals of that type in the feedlot for a unique model run.

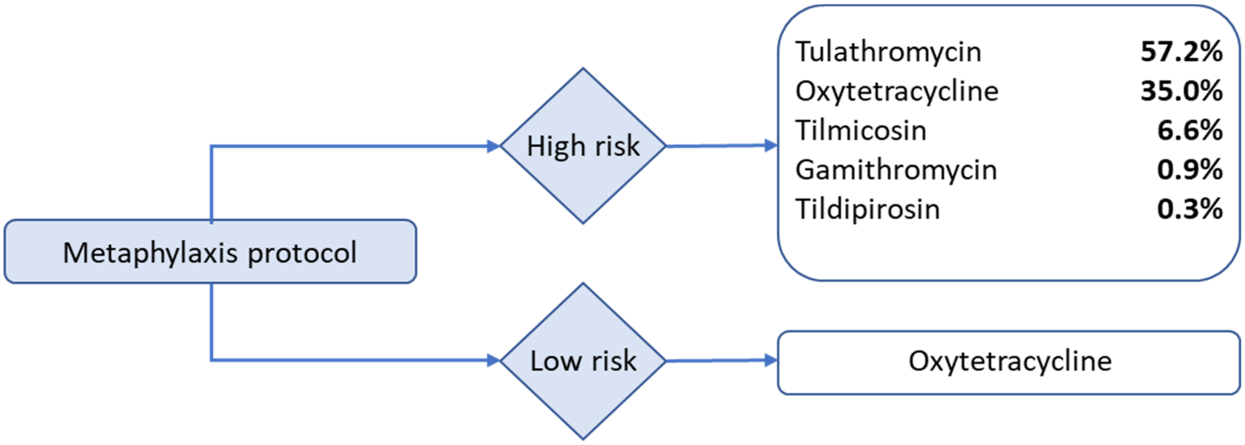

[1]Probabilities were derived from a sub-analysis of antimicrobial use trends on western Canadian feedlots (6). Historical use data were assembled from the literature to correspond with the timing of antimicrobial resistance data used in the model calibration experiments, and are not an endorsement of any particular regimen.

[2]BRD risk was determined by animal weight at feedlot arrival (31-32); in future experiments with the model, the infrastructure exists to incorporate a mixture of risks based on factors including animal sex, origin, and vaccination status.

Figure S4. Probability and duration of prophylactic (i.e., in-feed) protocols for the prevention of histophilosis and liver abscesses[1]. Protocol for each indication was probabilistically selected at model initialization for calibration and Monte-Carlo experiments and applied to all pens in the feedlot for a unique model run.

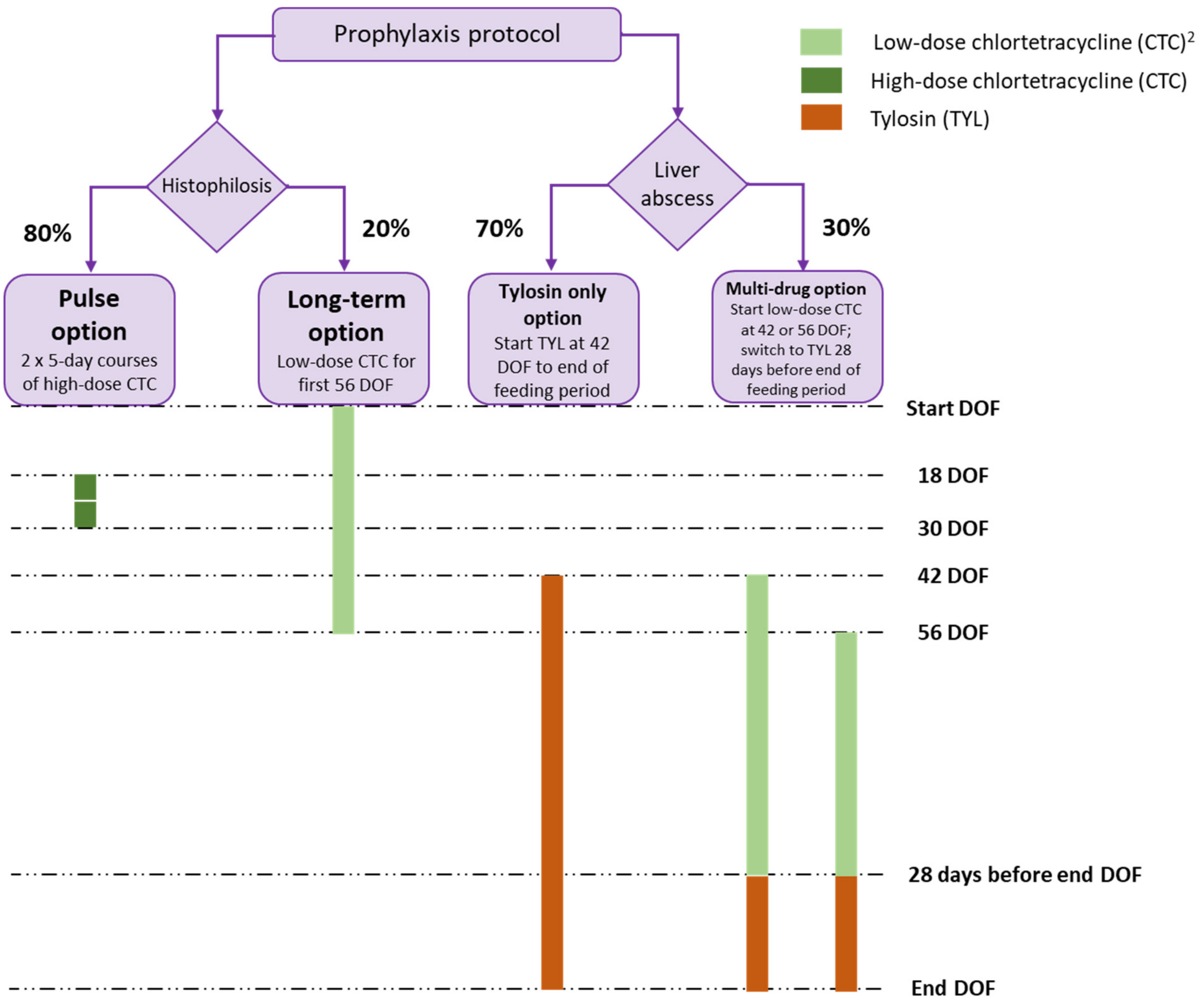

[1]Probabilities were estimated from sub-analyses of antimicrobial use trends on western Canadian feedlots (6), antimicrobial use data collected by the Canadian Integrated Program for Antimicrobial Resistance Surveillance (referenced in Hannon et al. (99), and a series of expert interviews with feedlot veterinarians. Historical use data were assembled from the literature to correspond with the timing of antimicrobial resistance data used in the model calibration experiments, and are not an endorsement of any particular regimen.

[2]Chlortetracycline (CTC) was administered prophylactically at both "low" and "high" dosages (indicated above with the light and dark green bars, respectively). When the "low dose" of CTC was used, the calibrated "selection probability" for tetracyclines was adjusted by a multiplier (0.2) that reflected the average concentration of that regimen relative to the "high dose" regimen.

Figure S5. Probability of therapeutic drug choice for first and subsequent BRD diagnoses based on 1) level of BRD risk at feedlot entry[1] and 2) animal weight at time of infection. Protocol by risk[2] and weight group was probabilistically selected at model initialization for calibration and Monte-Carlo experiments and applied to all animals in the feedlot for a unique model run.

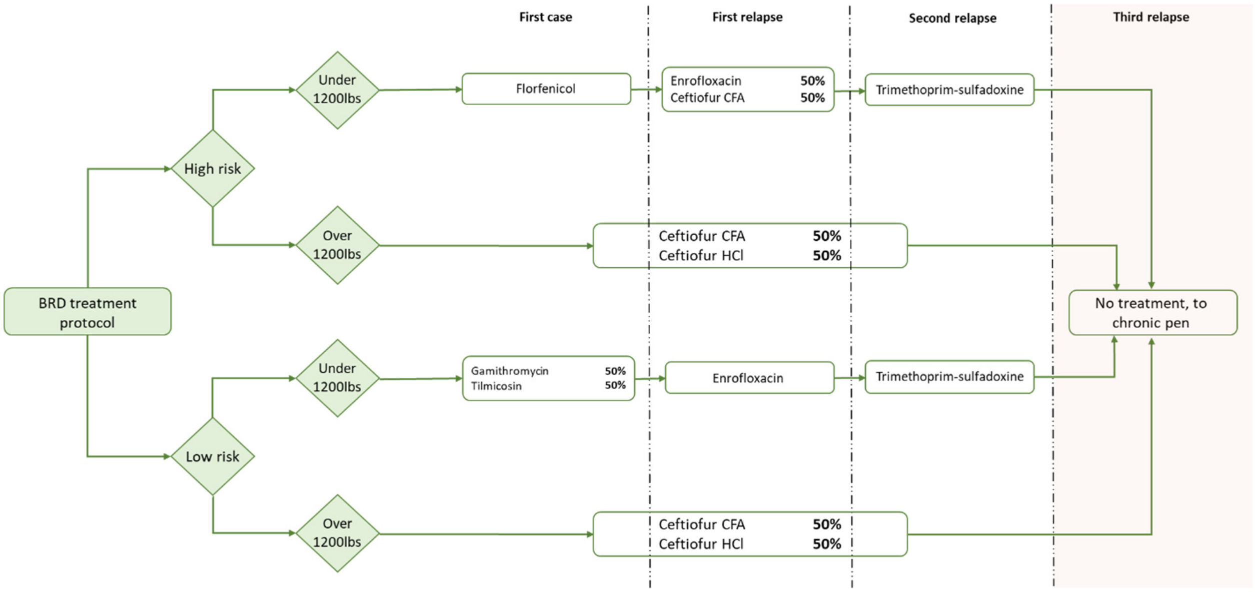

[1]BRD risk was determined by animal weight at feedlot arrival (31-32); in future experiments with the model, the infrastructure exists to incorporate a mixture of risks based on factors including animal sex, origin, and vaccination status.

[2]BRD treatment protocols were developed following a series of expert interviews with feedlot veterinarians. Historical use data were assembled to correspond with the timing of antimicrobial resistance data used in the model calibration experiments, and are not an endorsement of any particular regimen. Gamithromycin is no longer available in Canada.

Figure S6. Probability of therapeutic drug choice and location of therapy for first and subsequent arthritis diagnoses based on animal weight at time of diagnosis[1]. Protocol by weight group was probabilistically selected at model initialization for calibration and Monte-Carlo experiments and applied to all animals in the feedlot for a unique model run.

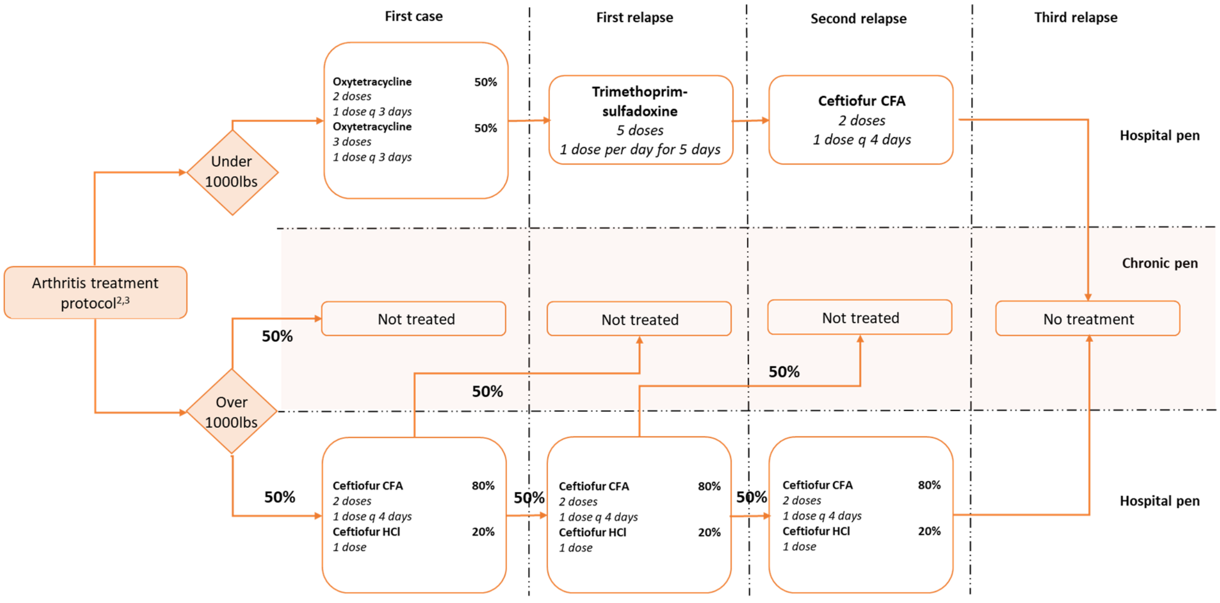

"q" = "every" in the above figure

[1]Arthritis treatment protocols were developed following a series of expert interviews with feedlot veterinarians. Historical use data were assembled to correspond with the timing of antimicrobial resistance data used in the model calibration experiments, and are not an endorsement of any particular regimen. Gamithromycin is no longer available in Canada.

[2]Animals that were probabilistically selected for arthritis treatment were relocated to the hospital pen and administered antimicrobials as part of multi-day regimens rather than single exposures; calves in the hospital pen were temporarily assigned an ADG equal to zero.

[3]Ceftiofur is not expected to be effective against uncomplicated *Mycoplasma bovis*-associated arthritis (100). However, arthritis diagnosed late in the feeding period in fall-placed calves can be confounded by other infectious agents, and some cases are likely sequelae of chronic and unresponsive foot rot.

Figure S7. **Probability of therapeutic drug choice for foot rot diagnosis based on animal weight at time of infection[1].** Protocol by weight group was probabilistically selected at model initialization for calibration and Monte-Carlo experiments and applied to all animals in the feedlot for a unique model run.

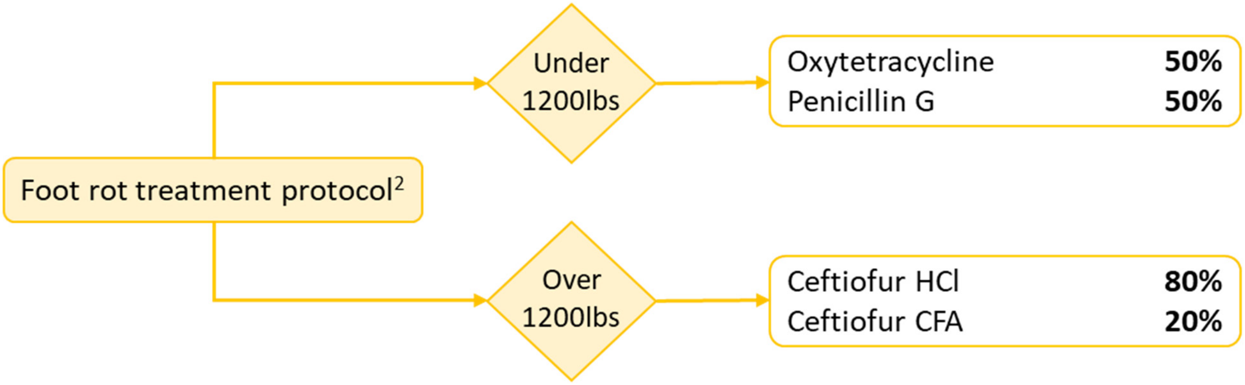

[1]Foot rot treatment protocols were developed following a series of expert interviews with feedlot veterinarians. Historical use data were assembled to correspond with the timing of antimicrobial resistance data used in the model calibration experiments, and are not an endorsement of any particular regimen.
[2]An "outbreak protocol" was triggered if 10% or more of the animals in a shared pen were diagnosed with foot rot in a single feeding period; in this scenario, a 7-day course of high-dose chlortetracycline (CTC) was administered at the pen-level (i.e., in-feed) to control the outbreak/prevent additional cases.

Figure S8. State charts for location (L) and mortality (R) in the Cattle agent. (L): Calves treated for arthritis were moved to the hospital pen for the duration of their treatment; calves that were not expected to respond to further treatment were moved to the chronic pen. Transition arrows demarcated by “envelope” symbols depend on the receipt of a “move to pen” message. **(R):** Calves could die from BRD, histophilosis or other causes in the model. Transition arrows demarcated by “graph” symbols are those governed by daily mortality rates derived from empirical data. Living calves periodically update their hazard rates according to the DOF and their condition.

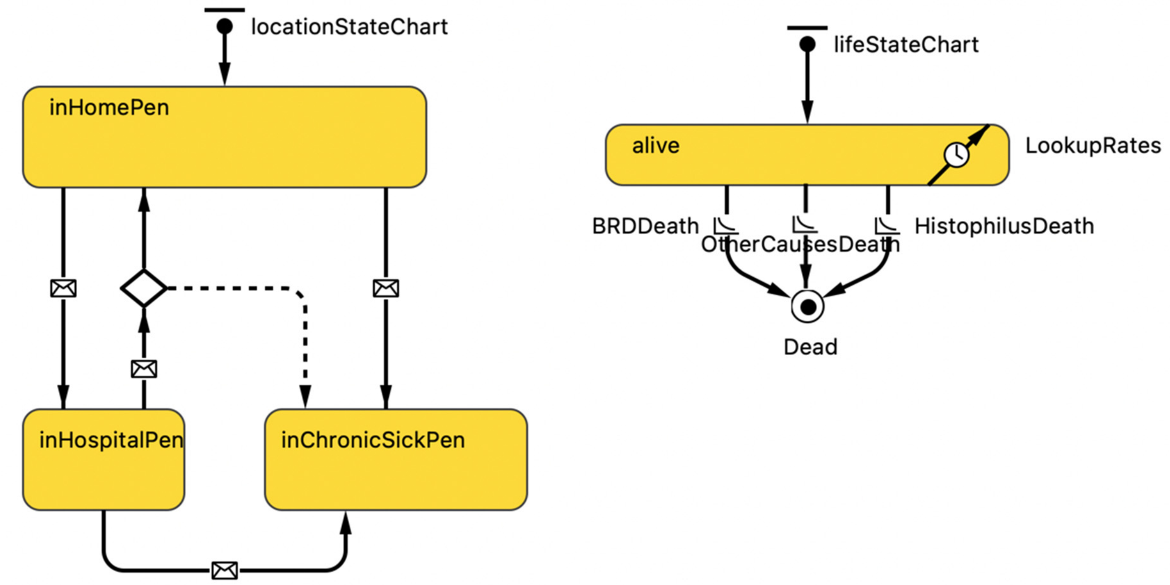

Figure S9. State chart for Treatment agent. The treatment agent administered a specific antimicrobial to a particular animal as outlined in the AMU protocols (**Figures S3-S7**). Treatments transitioned to the withdrawal state in one of two ways: 1) a prescribed amount of time had lapsed (arrow demarcated by a "clock" symbol), or 2) the animal reached a threshold weight (arrow demarcated by a "question mark" symbol).

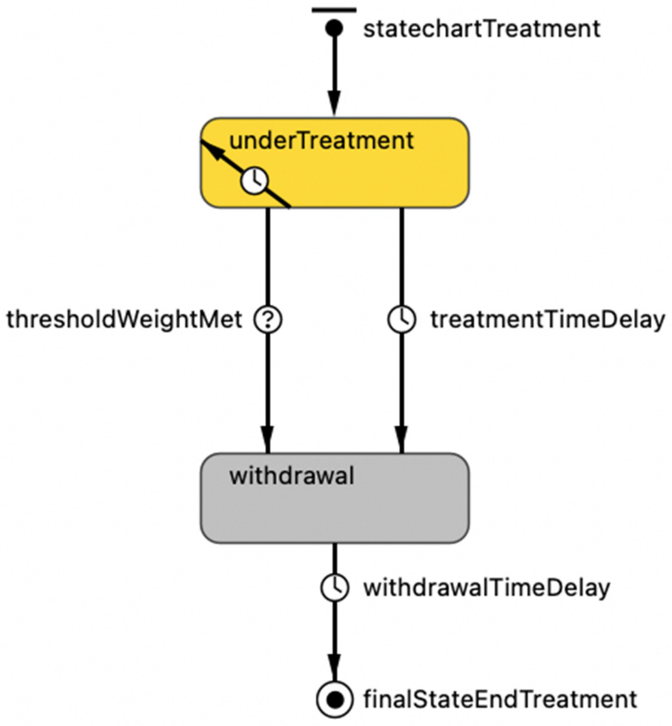

Table S1. Simulation parameters used to perform individual calibration experiments for each of the selected antimicrobial class and configuration combinations

	Value for model calibrations	Possible alternative settings	Implication
Initialization parameters			
Number of pens in feedlot	30	User selection<br><br>Number of pens increased to 50 in Monte Carlo experiments	Modifiable setting based on feedlot size or desired balance of population size and computational efficiency
Number of animals per pen	200	User selection	Modifiable setting based on pen size or desired balance of pen density and computational efficiency
Proportion of high-risk animals in simulation	100%	0-100%	Modifiable setting based on risk level of incoming animals
Proportion of steers in simulation	100%	0-100%	Modifiable setting based on sex of incoming animals
Default metaphylactic drug selection	Tulathromycin (i.e., Draxxin)	Probability table, **see Figure S3**	Probability of metaphylactic drug selection can be fixed or vary by BRD risk level per empirical data
Simulation parameters			
Number of iterations (i.e., model runs with unique combinations of target parameters)	2500	User selection<br><br>Number of iterations increased to 5000 in Monte Carlo experiments	Modifiable setting based on desired balance of adequate exploration of parameter space (i.e., methodological rigour) and computational efficiency
Number of realizations per iteration (i.e., model runs with identical combinations of target parameters but different "random seeds")	30	1	Modifiable setting based on desired balance of adequate exploration of stochastic variation within parameter set and computational efficiency
Optimization (objective) **criterion**	Minimize	Not applicable	The calibrated parameters are optimized (i.e., the best fit is achieved) when the objective function returns its smallest non-negative value
Optimization (objective) **function**	Compound function comprised of two components:<br>1) **curve fitness**, which quantifies the difference between linear datasets; and<br>2) **point fitness**, which quantifies the weighted difference between particular points in the datasets (1, 13, 70 and 170 DOF)	Not applicable	Function is the sum of the 1) average absolute difference between simulated and empirical datasets; and<br>2) the exponentiated absolute difference between specific reference points with stronger empirical data.<br><br>The point fitness weighting scheme can be modified to reflect variable user confidence in the empirical data points.

Table S2. Sensitivity of the prevalences (percentages) of detectable resistance at the *pen* level to select changes in model assumptions (i.e., scenarios) for each configuration/drug class combination. Medians and 95% prediction intervals summarize the range of likely outcomes derived from the repeated random sampling of model inputs across 5000 Monte Carlo simulations. Time points were selected to highlight the impact of model assumptions at varying levels of confidence in the empirical data (high confidence and low confidence for 13/70 and 50 DOF, respectively).

			Pen level					
			13 DOF[2]		50 DOF		70 DOF	
**Antimicrobial class**	**Configuration**	**Scenario[1]**	**Median %**	**95% PI**	**Median %**	**95% PI**	**Median %**	**95% PI**
**15-membered ring macrolides**	Drug use only	BL	56.3	(49.5, 63.0)	30.3	(24.0, 36.9)	21.6	(16.1, 27.6)
		BL, half TI	52.8	(45.7, 59.6)	28.3	(22.2, 34.7)	20.2	(14.7, 26.0)
		BL, dbl TI	60.0	(53.3, 66.8)	34.5	(28.0, 41.2)	24.6	(18.8, 30.8)
		AMR-R	56.3	(49.5, 63.0)	30.3	(24.0, 36.7)	21.6	(16.0, 27.5)
		Met-V	52.5	(0.5, 62.3)	26.6	(0, 35.9)	18.4	(0, 26.8)
	Transmission only	BL	61.1	(0, 76.0)	51.5	(38.2, 60.3)	31.0	(16.3, 42.0)
		BL, min step	61.0	(0, 76.0)	4.6	(0, 12.1)	0.5	(0, 6.6)
		BL, max step	61.1	(0, 76.0)	75.3	(65.6, 81.9)	75.3	(66.7, 81.9)
	Both	BL	57.1	(48.0, 65.8)	34.2	(23.7, 44.2)	21.9	(9.6, 34.0)
		BL, half TI	56.0	(46.5, 65.0)	34.2	(23.7, 44.2)	21.9	(9.6, 34.0)
		BL, dbl TI	100	(100, 100)	34.2	(23.7, 44.3)	21.9	(9.7, 33.8)
		AMR-R	57.1	(48.0, 65.8)	34.2	(23.7, 44.2)	22.0	(9.6, 34.0)
		Met-V	54.0	(0, 64.8)	34.0	(0, 44.2)	21.8	(0, 33.9)
		BL, min step	57.3	(48.0, 65.8)	7.6	(0, 18.9)	3.5	(0, 16.3)
		BL, max step	57.1	(48.0, 65.8)	55.8	(46.2, 64.8)	55.8	(46.2, 64.8)
**16-membered ring macrolides**	Drug use only	BL	4.0	(1.5, 7.5)	4.0	(1.5, 7.0)	3.6	(1.5, 6.7)
		BL, half TI	4.0	(1.5, 7.5)	4.0	(1.5, 7.1)	3.6	(1.5, 6.7)
		BL, dbl TI	4.0	(1.5, 7.5)	4.0	(1.5, 7.0)	3.6	(1.5, 6.6)
		AMR-R	4.0	(1.5, 7.5)	4.0	(1.5, 7.0)	3.6	(1.5, 6.7)
		Met-V	4.5	(1.5, 77.3)	4.0	(1.5, 71.9)	3.7	(1.5, 69.2)
	Transmission only	BL	13.1	(0, 30.2)	16.8	(0, 29.6)	11.7	(0, 25.4)
		BL, min step	13.1	(0, 30.5)	7.7	(0, 21.3)	5.1	(0, 20.0)
		BL, max step	13.1	(0, 30.2)	25.8	(0, 38.4)	26.0	(0, 38.6)
	Both	BL	12.1	(0, 29.0)	17.5	(0, 30.3)	12.6	(0, 25.9)
		BL, half TI	12.1	(0, 29.0)	17.4	(0, 30.2)	12.6	(0, 26.0)
		BL, dbl TI	12.1	(0, 29.0)	17.4	(0, 30.3)	12.6	(0, 25.9)
		AMR-R	12.1	(0, 29.0)	17.4	(0, 30.3)	12.6	(0, 25.9)
		Met-V	13.0	(0, 35.0)	17.6	(0, 30.3)	12.6	(0, 26.0)
		BL, min step	12.1	(0, 29.0)	8.6	(0, 21.8)	6.1	(0, 20.7)
		BL, max step	12.1	(0, 29.0)	26.0	(0, 38.7)	26.4	(0, 38.9)

			Pen level					
			13 DOF[2]		50 DOF		70 DOF	
**Sulfonamides**	Drug use only	BL	4.0	(1.5, 7.5)	4.5	(2.0, 7.7)	4.6	(2.0, 7.7)
		BL, half TI	4.0	(1.5, 7.5)	4.5	(2.0, 7.7)	4.6	(2.0, 7.8)
		BL, dbl TI	4.0	(1.5, 7.5)	4.1	(1.5, 7.6)	4.5	(2.0, 7.6)
		AMR-R	4.0	(1.5, 7.5)	4.5	(2.0, 7.7)	4.5	(2.0, 7.7)
		Met-V	4.5	(1.5, 7.5)	4.6	(2.0, 8.2)	5.0	(2.0, 8.5)
	Transmission only	BL	79.0	(72.5, 85.0)	48.5	(39.6, 57.1)	30.8	(18.8, 42.2)
		BL, min step	79.0	(72.5, 85.0)	0	(0, 1.5)	0	(0, 0)
		BL, max step	79.0	(72.5, 85.0)	79.2	(72.4, 85.3)	79.2	(72.4, 85.3)
	Both	BL	79.5	(72.9, 85.5)	47.2	(38.4, 55.8)	30.5	(18.4, 41.9)
		BL, half TI	79.5	(72.9, 85.5)	47.2	(38.4, 55.8)	30.4	(18.3, 41.8)
		BL, dbl TI	79.5	(72.9, 85.5)	47.2	(38.4, 55.8)	30.7	(18.5, 42.1)
		AMR-R	79.5	(72.9, 85.5)	47.2	(38.4, 55.9)	30.5	(18.4, 41.9)
		Met-V	79.5	(72.9, 85.5)	47.2	(38.4, 56.1)	30.6	(18.5, 42.0)
		BL, min step	79.5	(72.9, 85.5)	0	(0, 2.5)	0	(0, 1.5)
		BL, max step	79.5	(72.9, 85.5)	79.5	(72.9, 85.5)	79.5	(72.8, 85.4)
**Trimethoprim**	Drug use only	BL	0	(0, 1.0)	0.5	(0, 1.5)	0.5	(0, 2.0)
		BL, half TI	0	(0, 1.0)	0.5	(0, 2.0)	0.5	(0, 2.0)
		BL, dbl TI	0	(0, 1.0)	0	(0, 1.5)	0.5	(0, 1.5)
		AMR-R	0	(0, 1.0)	0.5	(0, 1.5)	0.5	(0, 1.6)
		Met-V	0	(0, 1.5)	0.5	(0, 2.6)	0.5	(0, 2.6)
	Transmission only	BL	0	(0, 4.5)	0	(0, 21.8)	0	(0, 17.8)
		BL, min step	0	(0, 4.5)	0	(0, 4.1)	0	(0, 2.5)
		BL, max step	0	(0, 4.5)	0	(0, 41.8)	0	(0, 64.6)
	Both	BL	0	(0, 4.5)	0.5	(0, 20.1)	0.5	(0, 15.9)
		BL, half TI	0	(0, 4.5)	1.0	(0, 20.2)	0.5	(0, 16.1)
		BL, dbl TI	0	(0, 4.5)	0.5	(0, 19.8)	0.5	(0, 15.7)
		AMR-R	0	(0, 4.5)	0.5	(0, 20.0)	0.5	(0, 15.8)
		Met-V	0	(0, 4.5)	1.0	(0, 20.5)	1.0	(0, 16.2)
		BL, min step	0	(0, 4.5)	0.5	(0, 4.7)	0	(0, 3.1)
		BL, max step	0	(0, 4.5)	1.0	(0, 37.6)	4.6	(0, 55.6)
**Tetracyclines**	Drug use only	BL	---	---	21.4	(4.0, 28.4)	16.0	(3.1, 22.2)
		BL, half TI	---	---	20.8	(4.0, 27.8)	15.7	(3.1, 21.8)
		BL, dbl TI	---	---	22.2	(3.6, 29.3)	16.7	(3.5, 23.0)
		AMR-R	---	---	19.8	(4.0, 26.8)	13.7	(3.0, 19.7)
		Met-V	---	---	20.9	(4.1, 29.6)	14.6	(3.5, 21.6)
	Transmission only	BL	---	---	28.0	(8.6, 44.5)	24.0	(7.6, 37.6)
		BL, min step	---	---	9.1	(2.0, 18.5)	6.2	(1.0, 13.7)

			Pen level					
			13 DOF[2]		50 DOF		70 DOF	
		BL, max step	---	---	44.7	(16.8, 62.1)	61.0	(36.5, 72.2)
	Both	BL	---	---	19.7	(7.0, 42.7)	17.3	(5.7, 40.8)
		BL, half TI	---	---	19.3	(6.6, 42.4)	16.8	(5.6, 39.8)
		BL, dbl TI	---	---	20.6	(7.5, 43.2)	18.2	(6.1, 42.7)
		AMR-R	---	---	19.7	(7.0, 42.6)	17.3	(5.6, 40.6)
		Met-V	---	---	20.7	(7.6, 42.6)	18.1	(6.2, 40.5)
		BL, min step	---	---	9.1	(3.0, 21.4)	6.6	(2.0, 18.4)
		BL, max step	---	---	29.3	(11.1, 56.8)	41.8	(18.7, 68.2)

[1]Abbreviations used to describe the scenario under investigation:
BL = baseline (i.e., calibration) version;
AMR-R = AMR-responsive version, where metaphylactic and therapeutic success is impacted by detectable AMR in individual calves;
Met-V = metaphylaxis variation version, where the choice of metaphylactic drug is varied per the probabilities in **Figure S3;**
half TI = variation of BL version where the therapeutic intervals (TI) were reduced by half;
dbl TI = variation of the BL version where the therapeutic intervals (TI) were doubled;
min step = variation of the BL version where the stress multiplier's impact was *minimized* by constraining the full strength of the step function (=10) to only 21 DOF;
max step = variation of the BL version where the stress multiplier's impact was *maximized* by maintaining the full strength of the step function (=10) through to 70 DOF (see **Figure 5** footnote).
[2]Simulated prevalences of resistance at 13 DOF are not reported for tetracycline class antimicrobials, as reference data for calibration was unavailable for that class/time point.

Table S3. Sensitivity of the prevalences (percentages) of detectable resistance at the *feedlot* level to select changes in model assumptions (i.e., scenarios) for each configuration/drug class combination. Medians and 95% prediction intervals summarize the range of likely outcomes derived from the repeated random sampling of model inputs across 5000 Monte Carlo simulations. Time points were selected to highlight the impact of model assumptions at varying levels of confidence in the empirical data (high confidence and low confidence for 13/70 and 50 DOF, respectively).

			Feedlot level					
			13 DOF[2]		50 DOF		70 DOF	
Antimicrobial class	Configuration	Scenario[1]	Median %	95% PI	Median %	95% PI	Median %	95% PI
15-membered ring macrolides	Drug use only	BL	56.3	(55.3, 57.3)	30.3	(29.4, 31.2)	21.6	(20.8, 22.5)
		BL, half TI	52.7	(51.7, 53.7)	28.3	(27.5, 29.3)	20.3	(19.5, 21.1)
		BL, dbl TI	60.1	(59.1, 61.0)	34.5	(33.6, 35.5)	24.7	(23.8, 25.6)
		AMR-R	56.3	(55.3, 57.3)	30.3	(29.3, 31.2)	21.6	(20.8, 22.5)
		Met-V	55.7	(1.8, 57.1)	29.7	(0.9, 31.1)	21.1	(0.6, 22.4)
	Transmission only	BL	56.3	(51.1, 60.7)	50.6	(47.7, 52.6)	30.5	(28.4, 32.4)
		BL, min step	56.2	(51.0, 60.6)	5.0	(4.1, 5.9)	1.4	(0.9, 2.0)
		BL, max step	56.3	(51.2, 60.8)	74.0	(70.3, 76.0)	74.4	(70.9, 76.1)
	Both	BL	57.1	(55.8, 58.4)	34.1	(32.6, 35.6)	21.9	(20.2, 23.6)
		BL, half TI	55.9	(54.6, 57.2)	34.1	(32.6, 35.6)	21.9	(20.2, 23.7)
		BL, dbl TI	100	(100, 100)	34.1	(32.7, 35.6)	21.9	(20.2, 23.7)
		AMR-R	57.1	(55.9, 58.4)	34.1	(32.6, 35.6)	21.9	(20.2, 23.7)
		Met-V	56.3	(36.6, 58.3)	33.5	(29.6, 35.4)	21.4	(18.7, 23.5)
		BL, min step	57.1	(55.8, 58.4)	7.9	(6.5, 9.4)	4.7	(3.4, 6.1)
		BL, max step	57.1	(55.9, 58.4)	55.8	(54.4, 57.1)	55.8	(54.4, 57.1)
16-membered ring macrolides	Drug use only	BL	4.2	(3.8, 4.6)	3.9	(3.5, 4.3)	3.8	(3.4, 4.2)
		BL, half TI	4.2	(3.8, 4.7)	3.9	(3.5, 4.3)	3.8	(3.4, 4.2)
		BL, dbl TI	4.2	(3.8, 4.6)	3.9	(3.5, 4.3)	3.8	(3.4, 4.2)
		AMR-R	4.2	(3.8, 4.7)	3.9	(3.5, 4.3)	3.8	(3.4, 4.2)
		Met-V	4.3	(3.8, 76.2)	4.0	(3.6, 70.7)	3.8	(3.4, 68.0)
	Transmission only	BL	13.4	(11.0, 15.9)	15.8	(13.6, 18.1)	11.5	(9.4, 13.6)
		BL, min step	13.3	(11.0, 15.8)	8.2	(6.4, 10.0)	6.2	(4.5, 8.0)
		BL, max step	13.3	(10.9, 15.8)	23.7	(20.6, 26.4)	24.1	(21.1, 26.8)
	Both	BL	12.6	(10.3, 14.9)	16.3	(14.0, 18.7)	12.1	(10.0, 14.3)
		BL, half TI	12.6	(10.3, 14.9)	16.4	(14.0, 18.6)	12.1	(10.0, 14.2)
		BL, dbl TI	12.6	(10.3, 14.9)	16.4	(14.0, 18.7)	12.1	(10.0, 14.2)
		AMR-R	12.6	(10.4, 14.9)	16.4	(14.0, 18.7)	12.1	(10.0, 14.2)
		Met-V	12.7	(10.4, 32.7)	16.5	(14.0, 19.5)	12.2	(10.0, 14.6)
		BL, min step	12.6	(10.4, 15.0)	8.7	(6.9, 10.6)	6.8	(5.0, 8.7)

			Feedlot level					
			13 DOF[2]		50 DOF		70 DOF	
		BL, max step	12.6	(10.3, 15.0)	23.9	(20.9, 26.7)	24.5	(21.5, 27.1)
**Sulfonamides**	Drug use only	BL	4.3	(3.9, 4.7)	4.5	(4.0, 5.0)	4.6	(4.2, 5.1)
		BL, half TI	4.3	(3.9, 4.8)	4.6	(4.2, 5.1)	4.6	(4.2, 5.1)
		BL, dbl TI	4.3	(3.9, 4.7)	4.4	(3.8, 5.0)	4.5	(4.0, 5.0)
		AMR-R	4.3	(3.9, 4.7)	4.5	(4.0, 5.0)	4.6	(4.2, 5.0)
		Met-V	4.3	(3.9, 4.8)	4.7	(4.1, 5.8)	4.8	(4.2, 5.9)
	Transmission only	BL	79.0	(77.4, 80.0)	48.4	(47.0, 49.6)	30.7	(29.0, 32.5)
		BL, min step	79.0	(77.3, 80.0)	0.1	(0.01, 0.3)	0	(0, 0.03)
		BL, max step	79.0	(77.4, 80.0)	79.1	(77.7, 80.0)	79.1	(77.9, 80.0)
	Both	BL	79.4	(77.7, 80.3)	47.2	(45.8, 48.5)	30.4	(28.7, 32.1)
		BL, half TI	79.4	(77.7, 80.3)	47.1	(45.8, 48.4)	30.3	(28.6, 32.0)
		BL, dbl TI	79.4	(77.7, 80.3)	47.2	(45.8, 48.4)	30.6	(28.8, 32.3)
		AMR-R	79.4	(77.7, 80.3)	47.2	(45.9, 48.4)	30.4	(28.6, 32.1)
		Met-V	79.4	(77.8, 80.3)	47.2	(45.9, 48.5)	30.5	(28.7, 32.2)
		BL, min step	79.4	(77.7, 80.3)	0.2	(0.05, 0.5)	0.07	(0, 0.3)
		BL, max step	79.4	(77.8, 80.3)	79.4	(78.3, 80.3)	79.4	(78.4, 80.3)
**Trimethoprim**	Drug use only	BL	0.3	(0.2, 0.4)	0.5	(0.3, 0.7)	0.6	(0.4, 0.7)
		BL, half TI	0.3	(0.2, 0.4)	0.6	(0.4, 0.7)	0.6	(0.4, 0.7)
		BL, dbl TI	0.3	(0.2, 0.4)	0.3	(0.1, 0.7)	0.5	(0.3, 0.7)
		AMR-R	0.3	(0.2, 0.4)	0.5	(0.3, 0.7)	0.5	(0.4, 0.7)
		Met-V	0.3	(0.2, 0.6)	0.6	(0.3, 1.5)	0.6	(0.4, 1.5)
	Transmission only	BL	0.7	(0.4, 1.1)	3.3	(1.7, 5.2)	2.7	(1.4, 4.3)
		BL, min step	0.7	(0.4, 1.1)	0.6	(0.3, 1.0)	0.3	(0.1, 0.6)
		BL, max step	0.7	(0.4, 1.1)	7.1	(3.9, 10.9)	14.7	(8.8, 21.6)
	Both	BL	0.7	(0.4, 1.1)	3.4	(1.8, 5.4)	2.8	(1.6, 4.4)
		BL, half TI	0.7	(0.4, 1.1)	3.6	(2.0, 5.4)	2.9	(1.7, 4.4)
		BL, dbl TI	0.7	(0.4, 1.1)	3.2	(1.5, 5.2)	2.7	(1.4, 4.3)
		AMR-R	0.7	(0.4, 1.1)	3.3	(1.8, 5.3)	2.8	(1.6, 4.3)
		Met-V	0.7	(0.4, 1.2)	3.7	(1.9, 6.6)	3.2	(1.7, 5.5)
		BL, min step	0.7	(0.4, 1.1)	0.8	(0.5, 1.3)	0.6	(0.3, 0.8)
		BL, max step	0.7	(0.4, 1.1)	7.0	(3.8, 10.7)	14.8	(8.9, 21.1)
**Tetracyclines**	Drug use only	BL	---	---	21.8	(5.4, 24.4)	16.5	(4.5, 18.5)
		BL, half TI	---	---	21.4	(5.5, 23.8)	16.1	(4.5, 18.1)
		BL, dbl TI	---	---	22.8	(5.4, 25.2)	17.2	(4.6, 19.1)
		AMR-R	---	---	20.3	(5.4, 23.0)	14.2	(4.3, 16.2)
		Met-V	---	---	20.6	(5.5, 26.8)	14.5	(4.4, 18.9)
		BL	---	---	27.6	(25.0, 30.2)	23.6	(21.5, 25.8)

			Feedlot level					
			13 DOF[2]		50 DOF		70 DOF	
	Transmission only	BL, min step	---	---	9.4	(8.3, 10.7)	6.5	(5.6, 7.5)
		BL, max step	---	---	43.3	(39.9, 46.6)	59.5	(56.8, 61.9)
	Both	BL	---	---	18.8	(16.8, 32.5)	16.2	(14.4, 31.5)
		BL, half TI	---	---	18.4	(16.3, 32.4)	15.9	(14.1, 30.9)
		BL, dbl TI	---	---	19.7	(17.5, 32.6)	16.9	(15.0, 32.9)
		AMR-R	---	---	18.8	(16.7, 32.4)	16.3	(14.4, 31.4)
		Met-V	---	---	19.9	(16.9, 32.4)	17.1	(14.6, 31.4)
		BL, min step	---	---	8.9	(7.8, 15.6)	6.5	(5.7, 13.3)
		BL, max step	---	---	27.6	(24.9, 44.8)	39.1	(35.9, 59.0)

[1]Abbreviations used to describe the scenario under investigation:
BL = baseline (i.e., calibration) version;
AMR-R = AMR-responsive version, where metaphylactic and therapeutic success is impacted by detectable AMR in individual calves;
Met-V = metaphylaxis variation version, where the choice of metaphylactic drug is varied per the probabilities in **Figure S3;**
half TI = variation of BL version where the therapeutic intervals (TI) were reduced by half;
dbl TI = variation of the BL version where the therapeutic intervals (TI) were doubled;
min step = variation of the BL version where the stress multiplier's impact was *minimized* by constraining the full strength of the step function (=10) to only 21 DOF;
max step = variation of the BL version where the stress multiplier's impact was *maximized* by maintaining the full strength of the step function (=10) through to 70 DOF (see **Figure 5** footnote).
[2]Simulated prevalences of resistance at 13 DOF are not reported for tetracycline class antimicrobials, as reference data for calibration was unavailable for that class/time point.

Table S4. Impact of AMR responsiveness on select *feedlot-* and *pen-level* outputs for 15-membered ring macrolides in an extreme macrolide use scenario. Medians and 95% prediction intervals are reported for 1) the prevalence of resistance and 2) the cumulative number of drug uses derived from the repeated random sampling of model inputs across 5000 Monte Carlo simulations. Time points were selected to highlight the impact of model assumptions at varying levels of confidence in the empirical data (high confidence and low confidence for 13/70 and 50 DOF, respectively).

		**13 DOF**				**50 DOF**				**70 DOF**			
	**Scenario[1]**	**Median %**	**95% PI**	**Median uses**	**95% PI**	**Median %**	**95% PI**	**Median uses**	**95% PI**	**Median %**	**95% PI**	**Median uses**	**95% PI**
Feedlot level													
Drug use only	BL	56.3	(55.3, 57.3)	9600	(9598, 9600)	30.3	(29.4, 31.2)	9600	(9598, 9600)	21.6	(20.8, 22.5)	9600	(9598, 9600)
	BL, mac15X	56.6	(55.7, 57.6)	9731	(9707, 9757)	32.3	(31.3, 33.2)	10545	(10468, 10625)	23.4	(22.6, 24.3)	10680	(10598, 10765)
	AMR-R, mac15X	56.6	(55.6, 57.6)	9754	(9727, 9783)	32.4	(31.4, 33.3)	10767	(10676, 10862)	23.5	(22.6, 24.4)	10912	(10813, 11014)
Both	BL	57.1	(55.8, 58.4)	9600	(9598, 9600)	34.1	(32.7, 35.6)	9600	(9598, 9600)	21.9	(20.1, 23.7)	9600	(9598, 9600)
	BL, mac15X	59.0	(57.7, 60.3)	9731	(9707, 9757)	38.6	(37.1, 40.1)	10546	(10467, 10763)	25.5	(23.7, 27.3)	10680	(10597, 10762)
	AMR-R, mac15X	59.6	(58.2, 60.8)	9775	(9744, 9807)	39.9	(38.4, 41.5)	10968	(10857, 11081)	26.0	(24.2, 27.8)	11130	(11015, 11249)
Pen level[2]													
Drug use only	BL	56.3	(49.5, 63.0)	--	--	30.3	(24.0, 36.9)	--	--	21.6	(16.1, 27.6)	--	
	BL, mac15X	56.5	(49.5, 63.5)	--	--	32.2	(25.9, 38.9)	--	--	23.4	(17.7, 29.4)	--	
	AMR-R, mac15X	56.5	(49.7, 63.5)	--	--	32.3	(26.0, 39.0)	--	--	23.5	(17.8, 29.6)	--	
Both	BL	57.1	(48.0, 65.8)	--	--	34.2	(23.7, 44.2)	--	--	21.9	(9.6, 34.0)	--	
	BL, mac15X	59.0	(49.7, 67.8)	--	--	38.6	(27.9, 49.0)	--	--	25.5	(13.0, 37.8)	--	
	AMR-R, mac15X	59.5	(50.3, 68.3)	--	--	40.0	(29.2, 50.3)	--	--	26.0	(13.5, 38.3)	--	

[1]Abbreviations used to describe the scenario under investigation:
BL = baseline (i.e., calibration) version;
Mac15X = variation of BL version where calves receive 15-membered ring macrolides for metaphylaxis *and* all BRD treatments under modified treatment algorithm;
AMR-R = AMR-responsive version, where metaphylactic and therapeutic success is impacted by detectable AMR in individual calves.
[2]Cumulative number of uses by antimicrobial class is not reported by the model at the *pen* level.

Table S5. Impact of AMR responsiveness on BRD incidence at the *feedlot* level in an extreme macrolide use scenario. Medians and 95% prediction intervals are reported for the cumulative number of first and subsequent (i.e., treatment failure/relapse) BRD cases at the end of the feeding period derived from the repeated random sampling of model inputs across 5000 Monte Carlo simulations.

		Median number of BRD cases				
**Configuration**	**Scenario[1]**	**First cases (95% PI)**	**First relapses (95% PI)**	**Second relapses (95% PI)**	**Third relapses (95% PI) (chronic cases)**	**Total relapses (95% PI)**
Drug use only	BL	848 (794, 904)	301 (269, 335)	106 (82, 126)	37 (26, 50)	444 (389, 501)
	BL, mac15X	848 (795, 906)	299 (266, 333)	106 (86, 127)	37 (25, 50)	441 (388, 497)
	AMR-R, mac15X	917 (860, 974)	406 (367, 446)	176 (150, 203)	73 (57, 91)	655 (587, 730)
Both	BL	848 (795, 901)	301 (268, 335)	106 (86, 127)	38 (26, 50)	445 (390, 501)
	BL, mac15X	849 (795, 903)	299 (265, 333)	106 (86, 125)	37 (26, 49)	442 (388, 496)
	AMR-R, mac15X	931 (874, 987)	514 (469, 559)	274 (240, 310)	144 (119, 170)	932 (838, 1028)

[1]Abbreviations used to describe the scenario under investigation:
BL = baseline (i.e., calibration) version;
Mac15X = variation of BL version where calves receive 15-membered ring macrolides for metaphylaxis *and* all BRD treatments under modified treatment algorithm;
AMR-R = AMR-responsive version, where metaphylactic and therapeutic success is impacted by detectable AMR in individual calves.
